# Kinetic mechanism of ENPP1 ATPase: Implications for aberrant calcification disorders and enzyme replacement therapy

**DOI:** 10.1016/j.jbc.2025.110558

**Published:** 2025-08-05

**Authors:** Marisa M. Michalchik, Tony Potchernikov, Ethan R. Lester, Demetrios T. Braddock, Wenxiang Cao, Enrique M. De La Cruz

**Affiliations:** 1 Department of Molecular Biophysics and Biochemistry, Yale University, New Haven, Connecticut, USA; 2 Department of Pathology, Yale University School of Medicine, New Haven, Connecticut, USA

**Keywords:** ENPP1, ATPase, calcification, GACI, kinetics, biomineralization, enzymology

## Abstract

Ectonucleotide pyrophosphatase phosphodiesterase 1 (ENPP1) is a transmembrane glycoprotein enzyme with an extracellular catalytic domain that hydrolyzes ATP into AMP and pyrophosphate (PP_*i*_). The ENPP1 ATPase is the major source of extracellular PP_*i*_, a critical physiological regulator of calcium phosphate crystal formation and biomineralization. ENPP1 deficiency lowers systemic PP_*i*_ levels and induces life-threatening arterial calcifications. Enzyme replacement therapy with a soluble ENPP1 biologic restores plasma PP_*i*_ and eliminates calcification and associated mortality in ongoing clinical trials in patients with ENPP1 deficiency. Despite the significant role of ENPP1 in inhibiting vascular calcification and regulating mammalian biomineralization *via* extracellular PP_*i*_ levels, little is known about the molecular mechanism of PP_*i*_ liberation by ENPP1. Here, we provide a kinetic analysis of the ENPP1 catalytic ATPase cycle. Our analysis shows that ATP cleavage, PP_*i*_ release, and hydrolysis of the covalent AMP-ENPP1 intermediate are rapid (>1000 s^−1^) and that AMP product release is slow and rate-limiting. The steady-state Michaelis constant of ATP substrate (*K*_M,T_) is comparable to physiological serum ATP levels of ∼100 nM, rendering ENPP1 activity sensitive to small changes in serum ATP. AMP binds strongly, with an affinity comparable to *K*_M,T_, such that ENPP1 is subject to intrinsic regulatory feedback by AMP under physiological concentrations of ∼100 nM. This product inhibition can attenuate ENPP1 during periods of high PP_*i*_ liberation, maintaining relatively constant plasma PP_*i*_ levels. The quantitative parameters of the ENPP1 ATPase cycle provided here allow for predictable outcomes of ENPP1 enzyme replacement therapy and provide plausible expectations for other PP_*i*_-linked calcification disorders.

Ectonucleotide pyrophosphatase phosphodiesterase (ENPP) enzymes are a subfamily of the alkaline phosphatase (AP) superfamily, consisting of seven extracellular, transmembrane (ENPP1, ENPP3-5, and ENPP7), GPI-anchored (ENPP6), or secreted (ENPP2) glycoproteins (1). They share a conserved catalytic phosphodiesterase domain with an active site architecture consisting of two zinc ions coordinated by seven histidine and aspartate residues and a catalytic threonine (serine in ENPP6) nucleophile (1). ENPPs hydrolyze phosphodiester bonds of various extracellular nucleotide and/or lipid substrates, often degrading or producing important signaling molecules, making their enzymatic activities important regulators of numerous critical physiological processes (1).

ENPP1 hydrolyzes ATP at the α-phosphate, generating PP_*i*_ and AMP (2). The ENPP1 ATPase is the sole source of extracellular PP_*i*_, the major physiological inhibitor of calcium-phosphate (Ca-P_*i*_) crystal formation and growth. As anticipated for a role in biomineralization, ENPP1 is expressed on the surface of mineralizing cell types like osteoblasts, where it plays a critical regulatory role in bone growth and remodeling. It is also found on the surface of vascular smooth muscle cells, inhibiting vascular calcification.

ENPP1 deficiency lowers systemic PP_*i*_ levels, resulting in severe arterial calcification and vascular intimal proliferation which induce life threatening vascular stenoses in generalized arterial calcification of infancy type 1 (GACI type 1; (3, 4)). Approximately 50% of infants with GACI type 1 (bi-allelic ENPP1 deficiency) will succumb to disease by 6 months of age (5), and almost invariably those who survive will develop a severe phosphate wasting rickets known as autosomal recessive hypophosphatemic rickets type 2 (ARHR2) (6, 7). Similarly, deficiency of the ATP exporter ABCC6 reduces plasma ATP levels, leading to arterial and cardiac calcifications in infants with GACI type 2 and strokes and cerebral calcifications in children with pseudoxanthoma elasticum ((3, 4)). Infants with GACI type 2 (bi-allelic ABCC6 deficiency) are less likely to die in infancy but are at risk for repeated strokes and cardiac calcification as children and progressive blindness due to retinal calcifications as adults (5, 8).

Enzyme replacement therapy (ERT) with an ENPP1 biologic restores systemic PP_*i*_ levels and eliminates the calcification phenotype and mortality in a mouse model of GACI (*enpp1*^*asj/asj*^) (9). Ongoing ERT clinical trials in adults with ENPP1 (ARHR2) and ABCC6 deficiency (pseudoxanthoma elasticum) demonstrate that subcutaneous ENPP1 administration restores physiological PP_*i*_ levels in these patients, with a dose-response unique to the underlying genetic deficiency (https://investors.inozyme.com/events-presentations).

Despite the demonstrated importance of ENPP1 in biomineralization, knowledge of the molecular mechanism of PP_*i*_ liberation by ENPP1 is lacking. A catalytic mechanism has been proposed from active site similarity to AP and structural studies of ENPP enzymes (10, 11), but little or no information regarding the biochemical rate and equilibrium constants defining the catalytic reaction pathway are known. Accordingly, physiological models of dynamic PP_*i*_ liberation and overall ENPP1 function, including during ERT for GACI, are poorly developed and cannot be evaluated.

Here, we use transient kinetic and quantitative equilibrium analyses to determine the kinetic mechanism of PP_*i*_ liberation by ENPP1 from ATP. Our results indicate that ENPP1 is tailored to rapidly and efficiently bind ATP and liberate PP_*i*_. ENPP1 also binds AMP rapidly and with high affinity, which allows AMP product to serve as an intrinsic regulatory feedback inhibitor of ENPP1 activity, inhibiting ENPP1 during periods of high ATPase activity and maintaining stable PP_*i*_ levels. This quantitative and mechanistic knowledge provides a biochemical framework for developing predictable outcomes of ERT with ENPP1- and ABCC6-deficient patients and generating testable predictions for other PP_*i*_-linked calcification disorders.

## Results

### Steady-state ATP hydrolysis by ENPP1

**Figure 1:**
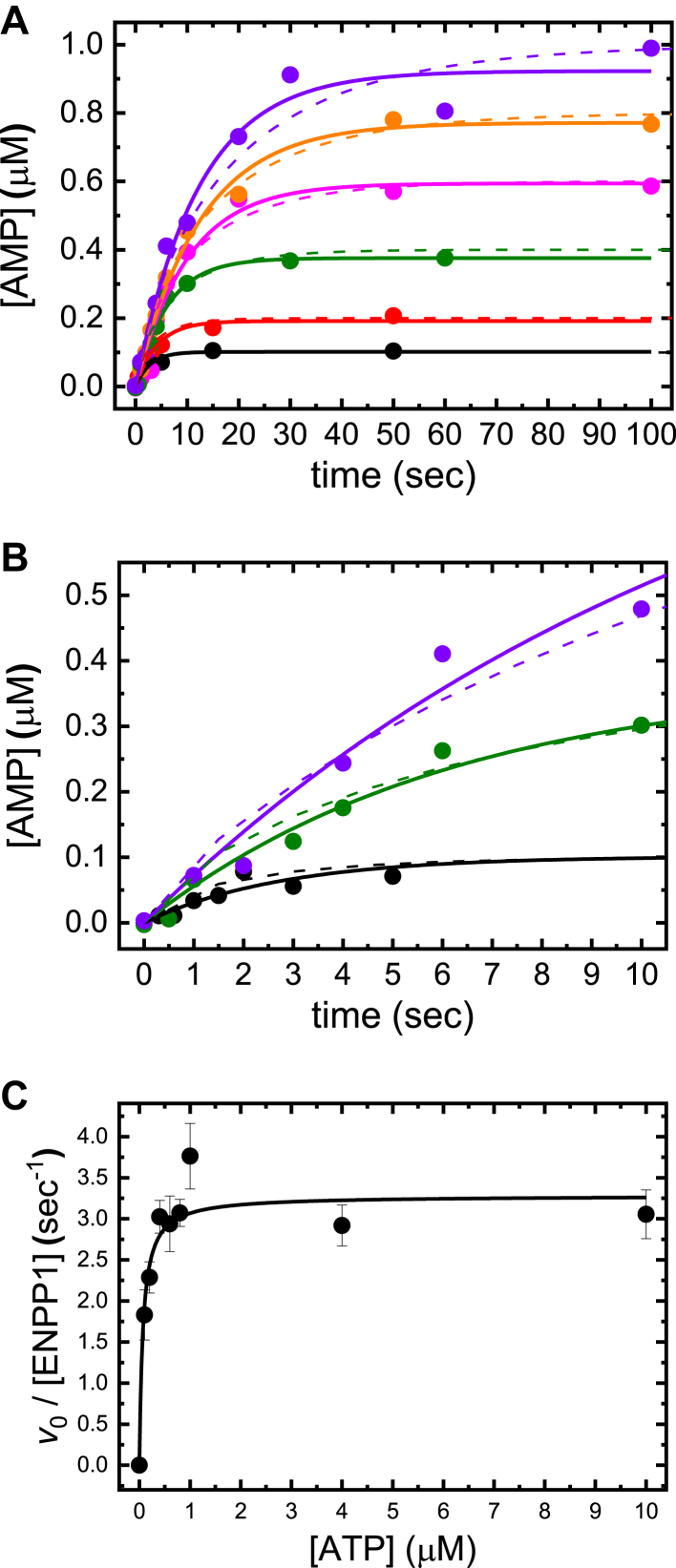
**Steady-state catalysis of ATP hydrolysis by ENPP1**. *A*, time courses of AMP liberation after mixing ENPP1 (20 nM) with a range of [ATP] (0.1–1 μM; containing trace amounts of [α-^32^P]-ATP). The *solid lines* through the data points represent the best fit to Equation 11. *Dashed lines* in *panels A* and *B* represent kinetic simulations of Figure 2 using KinTek Explorer Chemical Kinetics Software and the rate and equilibrium constants defined in Table 2. *B*, zoomed in view of the initial 10 s of AMP product liberation in panel (*A*). Only 0.1, 0.4, and 1 μM time traces are shown for ease of viewing. *C*, [ATP]-dependence of the initial rate of product liberation (*v*_0_; Equation 11), obtained from fits in *panel A*, per enzyme active site. The *solid line* through the data represents the best fit to the Briggs-Haldane equation (Eq. 12), yielding a *K*_M,T_ value of 70 ± 23 nM and a *k*_cat,T_ value of 3.3 ± 0.2 s^−1^. ENPP1, ectonucleotide pyrophosphatase phosphodiesterase 1.

Time courses of steady-state ENPP1-catalyzed AMP and PP_*i*_ liberation from the hydrolysis of ATP are nonlinear over the concentration range and time scales examined (Fig. 1*A*). The entire time courses were analyzed following methods developed for determining the initial velocity (*v*_0_) from nonlinear steady-state enzyme kinetic time courses (12). The *v*_0_ depends hyperbolically on the initial [ATP], yielding a Michaelis constant for ATP substrate (*K*_M,T_) of 70 ± 23 nM and a turnover rate (*k*_cat,T_) of 3.3 ± 0.2 s^−1^ (Fig. 1*C*; Table 1). The *k*_cat,T_ value is consistent with previous determinations obtained under similar solution conditions; the previous *K*_M,T_ estimate of <2 μM is also consistent (9). 2′/3′-O-(N-Methyl-anthraniloyl)-adenosine-5′-triphosphate (mant-ATP) has *K*_M,mT_ of < 1 μM and *k*_cat__,__mT_ of 3.0 ± 0.3 s^−1^ (Fig. S1; Table 1), consistent with the mant fluorophore having little to no effect on the rate of steady-state catalysis.

**Table 1:** Rate and equilibrium constants of the ENPP1 ATPase cycle

Parameter	Value	Experiment
*k*_cat,T_	3.3 ± 0.2 s^−1^ 3.0 ± 0.3 s^−1^ 4.0 ± 0.3 s^−1^	Steady-state ATPase ([ENPP1] = 20 nM; Fig. 1) Steady-state mantATPase ([ENPP1] = 0.1 μM; Fig. S1) Steady-state ATPase ([ENPP1] = 4 μM; Fig. 7)
*K*_M,T_	70 ± 23 nM	Steady-state ATPase (Fig. 1)
*K*_T,c_	>>10 μM	ATP association (Fig. 4)
*k*_+__T_	>>1000 s^−1^	ATP association (Fig. 4)
$\frac{k_{+T}}{K_{T,c}}$	97 ± 9 μM^−1^ s^−1^	ATP association (Fig. 4)
*k*_−cleavage_, *k*_+__PP*i*_, *k*_−hydrolysis_	∼0 s^−1^	Single turnover ATPase – quenched flow (Fig. 7, *A*–*C*)
*k*_+__cleavage_, *k*_−PP*i*_, *k*_+__hydrolysis_	≥1000 s^−1^	Single turnover ATPase – quenched flow (Fig. 7, *A*–*C*)
*K*_PP*i*_	>> 100 μM	Steady-state ATPase with PP_*i*_ inhibition (Fig. S4)
*K*_AMP′ =_$\frac{k_{-{\text{AMP}}^{\prime }}}{k_{+{\text{AMP}}^{\prime }}}$	∼1	Irreversible AMP dissociation (Fig. 6*A*; using values from steady-state ATPase, Fig. 1)
*k*_−AMP′_	∼4 s^−1^	Irreversible AMP dissociation (Fig. 6*A*; using values from steady-state ATPase, Fig. 1)
*k*_+__AMP′_	∼4 s^−1^	Irreversible AMP dissociation (Fig. 6*A*; using values from steady-state ATPase, Fig. 1)
*k*_−AMP_	∼8 s^−1^	Irreversible AMP dissociation (Fig. 6*A*; using values from steady-state ATPase, Fig. 1)
*k*_+__AMP_	∼565 s^−1^	AMP association (Fig. 5)
*K*_AMP_ = $\frac{k_{-\text{AMP}}}{k_{+\text{AMP}}}$	∼0.014 μM	Calculated from *k*_−AMP_ and *k*_+__AMP_
*K*_AMP,c_	3 ± 3 μM	AMP association (Fig. 5)
$\frac{k_{+\text{AMP}}}{K_{\text{AMP},c}}$	126 ± 23 s^−1^	AMP association (Fig. 5)
*K*_AMP, overall_ = $K_{M,c}\frac{K_{\text{AMP}}K_{{\text{AMP}}^{\prime }}}{K_{{\text{AMP}}^{\prime }}+1}$	∼0.021 μM	Calculated from individual binding affinities
*k*_cat,mT_	3.0 ± 0.3 s^−1^	Steady-state mant–ATPase ([ENPP1] = 10 nM; Fig. S1)
*K*_M,mT_	<1 μM	Steady-state mant–ATPase (Fig. S1)
$\frac{k_{+\text{mT}}}{K_{\text{mT},c}}$	58 ± 2 μM^−1^ s^−1^	mant-ATP association (Fig. S2)
*K*_mAMP,c_	5 ± 5 μM	mant-AMP association (Fig. S2)
$\frac{k_{+\text{mAMP}}}{K_{\text{mAMP},c}}$	78 ± 8 s^−1^	mant-AMP association (Fig. S2)
$\frac{k_{+\text{methT}}}{K_{\text{methT},c}}$	141 ± 22 μM^−1^ s^−1^	α,β-methylene ATP association (Fig. 4)
*k*_−methT_	121 ± 16 s^−1^ 174 ± 10 s^−1^	α,β-methylene ATP association (Fig. 4) Irreversible α,β,methylene ATP dissociation (Fig. 4*D*)
*K*_methT_	∼1 μM	α,β-methylene ATP association (Fig. 4) Irreversible α,β-methylene ATP dissociation (Fig. 4*D*)

Conditions: 20 mM Tris–HCl (pH 7.4), 154 mM NaCl, 0.014 mM ZnCl_2_, 1 mM MgCl_2_, 1 mM CaCl_2_, and 4.5 mM KCl. Uncertainties reported here originate from standard errors in fits, propagated using Equation 14.

**Table 2:** ENPP1 ATPase cycle reaction constants used for kinetic simulations

Parameter	Value
$\frac{k_{+T}}{K_{T,c}}$ as *k*_+__T_	97 μM^−1^ s^−1^
*k*_−T_	88 s^−1^
*k*_+__cleavage_	1000 s^−1^
*k*_−cleavage_	0.1 s^−1^
*k*_−PP*i*_	2560 s^−1^
*k*_+__PP*i*_	0.1 s^−1^
*k*_+__hydrolysis_	2570 s^−1^
*k*_−hydrolysis_	0.1 s^−1^
*k*_−AMP′_	4 s^−1^
*k*_+__AMP′_	4 s^−1^
*k*_−AMP_	8 s^−1^
$\frac{k_{+\text{AMP}}}{K_{\text{AMP},c}}$ as *k*_+__AMP_	192 μM^−1^ s^−1^

### ENPP1 ATPase reaction cycle

**Figure 2:**
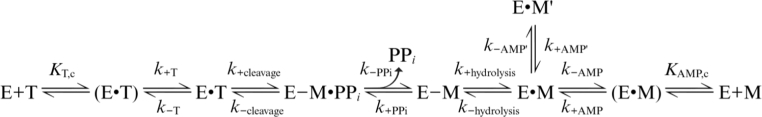
**The ENPP1 ATPase cycle**. Equilibrium constants (*K*_i_) are defined by the ratio of rate constants (*k*_*+*__*i*_/*k*_−__*i*_), with *k*_*+*_ corresponding to progression through the cycle from *left* to *right*, except for reversible AMP and PP_*i*_ product release and binding. Covalent intermediates are indicated by a *dash* (−) and noncovalent binding is indicated by a *dot* (∙). The parentheses around (E∙T) and (E∙M) indicate that these exist in rapid equilibrium with free ENPP1 (E) and ligand (T or M). We show all biochemical transitions from E∙T to E∙M are rapid and completed within ∼15 msec of mixing, allowing initial ATP cleavage (*K*_cleavage_), PP_*i*_ release (*K*_PP*i*_), and covalent intermediate hydrolysis (*K*_hydrolysis_) to be modeled as a single, rapid transition (*K*_internal_ = *K*_cleavage_*K*_PP*i*_*K*_hydrolysis_). PP_*i*_ remains in solution after release from ENPP1 but is omitted from the downstream AMP-bound states for visual simplicity. ENPP1, ectonucleotide pyrophosphatase phosphodiesterase 1.

The experiments and analysis carried out in this study demonstrate that the minimum catalytic ENPP1 ATPase cycle is defined by (at least) eight reversible biochemical transitions (Fig. 2): ATP (T) initially binds (*K*_T,c_) ENPP1 in a low-affinity collision complex ((E∙T)) before rapidly isomerizing to a high-affinity ATP-bound state (E∙T). ENPP1-bound ATP is cleaved (*K*_cleavage_) *via* nucleophilic attack by the catalytic threonine (T256), forming the covalent, nucleotidylated ENPP1-AMP intermediate and noncovalently bound PP_*i*_ (E−M∙PP_*i*_). PP_*i*_ product is subsequently released (*K*_PP*i*_) and the covalent intermediate (E−M) is hydrolyzed (*K*_hydrolysis_), yielding ENPP1 with noncovalently bound AMP (E∙M). This complex can then either dissociate bound AMP following a two-step mechanism (similar to ATP binding) to regenerate free ENPP1 or isomerize (*K*_AMP′_) to an off-pathway AMP-bound state (E∙M′). This scheme is proposed based on the conserved catalytic mechanism of the AP super family and modified based on our experimental results (13).

The rate and equilibrium constants of the biochemical transitions defining the ENPP1 catalytic ATPase cycle outlined in Figure 2 were determined from a series of rapid mixing (stopped- and quenched-flow) experiments (see Experimental procedures). The equations used for analyzing experimental data and extracting the microscopic reaction rate and equilibrium constants are derived and presented in the supplementary information (SI). In the main text, we include only the equations used for data analysis.

### (mant-)AMP does not dissociate from ENPP1 under single turnover conditions

**Figure 3:**
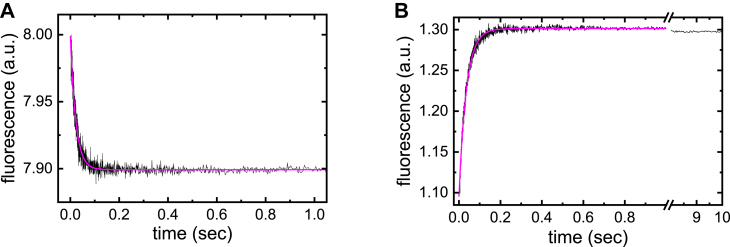
**(mant-)AMP product does not dissociate from ENPP1 under single turnover conditions**. *A*, time course of ENPP1 intrinsic tryptophan fluorescence change after mixing ENPP1 (0.3 μM) with ATP (0.05 μM) under single-turnover conditions ([ENPP1] > [ATP]). A fluorescence decay associated with ATP binding is observed, but a subsequent fluorescence enhancement corresponding to AMP product release was not observed over the time of data collection (2 s), which was much longer than the 0.3 s turnover time calculated from *k*_cat,T_. The *smooth line* through the data represents the best fit to a single exponential, with an observed rate constant (*λ*_obs_; this is a composite of fundamental rate constants yielding an observed relaxation (14)) of 44.8 ± 0.7 s^−1^. *B*, time course of mant fluorescence change after mixing ENPP1 (0.3 μM) with mant-ATP (0.05 μM) under single turnover conditions ([ENPP1] >> [mant-ATP]). As seen in panel *A*, a fluorescence increase associated with mant-ATP binding is observed, but a subsequent fluorescence decay corresponding to mant-AMP product release was not observed over the time of data collection (10 s). The *smooth line* through the data represents the best fit to a single exponential with an *λ*_obs_ of 28.1 ± 0.2 s^−1^. mant-ATP, 2'/3′-O-(N-Methyl-anthraniloyl)-adenosine-5′-triphosphate.

**Figure 4:**
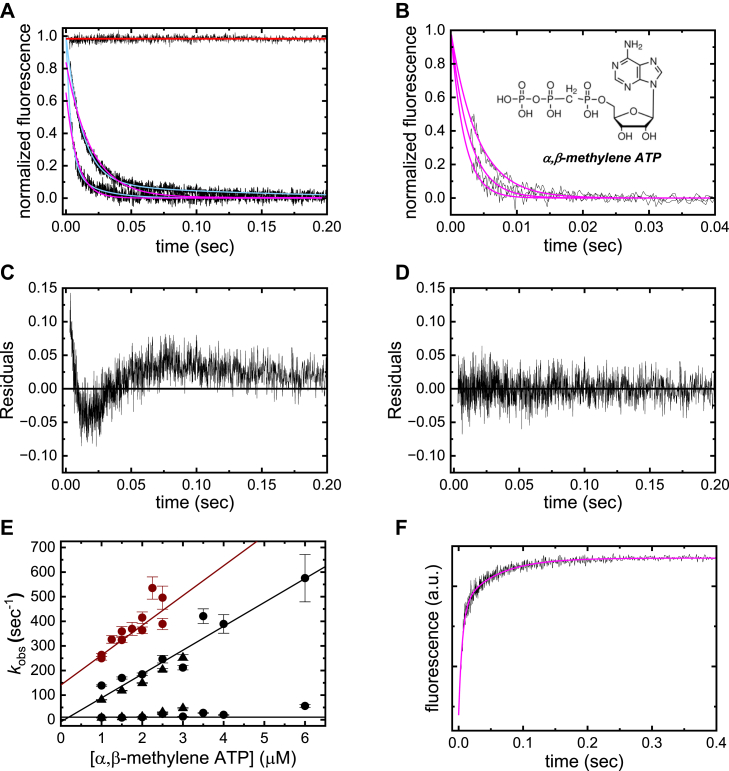
**ATP and α,β-methylene ATP binding to ENPP1**. *A*, representative time courses of ENPP1 intrinsic tryptophan fluorescence quenching after mixing ENPP1 (0.2 μM) with ATP (0–6 μM; *right* to *left*: 0, 1, and 3 μM shown). Smooth lines through the data represent the best fits to a double exponential (*light blue*) and a single exponential (*magenta*). *B*, representative time courses of ENPP1 intrinsic tryptophan fluorescence quenching after mixing ENPP1 (0.2 μM) with α,β-methylene ATP (0–2.5 μM; right to left: 1, 1.5, and 2.5 μM shown). *Smooth lines* through the data represent the best fits to a single exponential. *C*, residuals of the best fit of the 1 μM trace in panel A to a single exponential. *D*, residuals of the best fit of the 1 μM trace in *panelA* to a double exponential. *E*, [ATP]-dependence of *λ*_obs,T,fast_ and *λ*_obs,T,slow_ from double exponential fits (*black* symbols; *circles* and *triangles* differentiate points from two individual data sets acquired on two different days). The value of *λ*_obs,T,fast_ depends linearly on the [ATP], yielding a slope (*k*_+__T_/*K*_T,c_) of 97 ± 9 μM^−1^ s^−1^. The slow phase has a variable amplitude ranging from 8 to 17% of the total fluorescence, with a mean *λ*_obs,T,slow_ value of 10 ± 1 s^−1^ that depends weakly on the [ATP] over the range examined. [α,β-methylene ATP]-dependence of the observed binding rate constant (*λ*_obs_) obtained from single exponential fits (*maroon circles*). The value of *λ*_obs_ depends linearly on the [α,β-methylene ATP], yielding a slope (*k*_+__methT_/*K*_methT,c_) of 141 ± 22 μM^−1^ s^−1^ and an intercept value (*k*_–__methT_) of 121 ± 16 s^−1^. *F*, time course of mant-AMP fluorescence enhancement after mixing a preequilibrated solution of ENPP1 (0.1 μM) and α,β-methylene ATP (20 μM) with excess competing mant-AMP (1 mM). The *smooth line* through the data is the best fit to a double exponential with *λ*_obs,fast_ and *λ*_obs,slow_ of 174 ± 10 s^−1^ (proxy for α,β-methylene ATP dissociation) and 18 ± 0.5 s^−1^ (slow phase of mant-AMP binding). Time courses shown in panel *A*, *B*, and *D* are the average of at least three individual time courses. ENPP1, ectonucleotide pyrophosphatase phosphodiesterase 1; mant-AMP, 2'/3′-O-(N-Methyl-anthraniloyl)-adenosine-5′-monophosphate.

ATP binding quenches the intrinsic tryptophan fluorescence of ENPP1 (Figs. 3*A* and 4*A*). After rapidly mixing ENPP1 with ATP under single turnover conditions ([ENPP1] = 0.3 μM >> [ATP] = 0.05 μM) so that ENPP1 goes through its catalytic cycle only once, time courses of intrinsic fluorescence quenching follow a single exponential decay (Fig. 3*A*). The observed rate constant of the exponential decay (*λ*_obs_) is a function of fundamental rate constants for binding, catalysis, and conformational changes resulting in an observed behavior (14). Notably, this decay was not followed by an increase in fluorescence associated with product release and reformation of free ENPP1 over the 2 s time course examined. This time interval is significantly longer than the 0.3 s turnover time calculated from *k*_cat,T_, suggesting AMP product remains bound to ENPP1 after catalysis under these conditions and binds ENPP1 with high affinity (*K*_AMP_ < 0.3 μM).

mant-ATP binding to ENPP1, measured by intrinsic Förster resonance energy transfer (iFRET) from ENPP1 tryptophan residues to the fluorescent mant moiety, is associated with a fluorescence enhancement (Fig. 3*B*; Fig. S2), as with other ATPases (15, 16, 17, 18). Time courses of fluorescence enhancement after mixing ENPP1 with mant-ATP under single-turnover conditions ([ENPP1] = 0.3 μM >> [mant-ATP] = 0.05 μM) also follow a single exponential and show no subsequent fluorescence decay associated with 2′/3′-O-(N-Methyl-anthraniloyl)-adenosine-5′-monophosphate (mant-AMP) product release and reformation of free ENPP1 (Fig. 3*B*), suggesting a similarly high binding affinity for mant-AMP product (*K*_mAMP_ < 0.3 μM).

The observed rate constants (*λ*_obs_) of mant-labeled and unlabeled nucleotide binding to 0.3 μM ENPP1 differ by < 2-fold, consistent with the fluorophore modestly interfering with nucleotide (ATP and AMP) binding (Fig. 3).

### ATP (substrate) binding to ENPP1

Time courses of ENPP1 (0.1 or 0.2 μM) intrinsic fluorescence quenching after rapidly mixing with ATP (1–6 μM) fit best to double exponentials (Fig. 4*A*), as indicated from the randomly distributed residuals of the best fit (Fig. 4*D*). The residuals of the best single exponential fit are not random, but display “ringing”, indicative of an additional relaxation (Fig. 4*C*). Time courses of nonhydrolyzable (Fig. S3) analogue α,β-methylene ATP follow single exponentials (Fig. 4*B*), indicating the slow phase arises from a post hydrolysis transition. The fast phase is associated with ATP binding and formation of the E⋅T state (Fig. 2).

Although the fast phase observed rate constant (*λ*_obs,T,fast_) depends linearly on [ATP], we infer the formation of an initial, spectroscopically silent ATP-bound collision complex ((E∙T), Fig. 2) in rapid equilibrium with free ENPP1 based on the mechanism of AMP binding (presented below) and substrate binding to other AP family enzymes (19). Accordingly, *λ*_obs,T,fast_ is predicted to depend hyperbolically on [ATP] (Eq. S11; (14)) with a half-way point equal to the affinity for the initial collision complex, as observed for two-step nucleotide binding mechanisms identified in other ATPases (20, 21, 22, 23). The linear [ATP]-dependence over the 0 to 6 μM [ATP] range evaluated (Fig. 4*E*), indicates the affinity of the initial collision complex is weaker than the highest [ATP] evaluated (dissociation binding constant *K*_T,c_ >> 10 μM). Rapid ATP binding (∼600 s^−1^ at 6 μM ATP; Fig. 4*E*) precluded evaluation at ATP concentrations > 6 μM where hyperbolic behavior could be detected, as we are limited by the ∼3 millisecond dead time of the stopped-flow.

Since [ATP] << *K*_T,c_ in these experiments, *λ*_obs,T,fast_ depends linearly on [ATP] at low concentrations according to Equation 1 & S11′:

(1) $$\lambda_{\text{obs},T,\text{fast}}=\frac{k_{+T}}{K_{T,c}}\left[T\right]+k_{+{\text{AMP}}^{\prime }}+k_{-\text{AMP}}$$

The slope of the best fit of the data to a linear function yields a second-order ATP association rate constant ($\frac{k_{+T}}{K_{T,c}}$) of 97 ± 9 μM^−1^ s^−1^ (Fig. 4*E*). Based on this slope value and *K*_T__,__c_ >> 10 μM, *k*_+__T_ has a value of >1000 s^−1^ (Table 1). According to Equation 1, the extrapolated intercept value should be ≈ *k*_+__AMP′_ + *k*_−AMP_ (≈12 s^−1^, discussed below; Table 1) and is indistinguishable from this value given the uncertainty (Fig. 4*E*). Below, we show that formation of the covalent intermediate, subsequent PP_*i*_ release, and hydrolysis are very rapid (>1000 s^−1^), Therefore, the slow phase reflects biochemical transitions associated with the AMP-bound state (discussed below).

Time courses of ENPP1 (0.2 μM) intrinsic fluorescence quenching after rapidly mixing with α,β-methylene ATP (1–2.5 μM) fit to single exponentials (Fig. 4*B*) with an *λ*_obs_ that depends linearly on the [α,β-methylene ATP] over the range examined (Fig. 4*E*). The best fit to a linear function yields a slope (*k*_+__methT_/*K*_methT__,__c_) of 141 ± 22 μM^−1^ s^−1^, consistent with that of ATP binding (*k*_+__T_/*K*_T__,__c_ = 97 ± 9 μM^−1^ s^−1^), and an intercept value (*k*_–__methT_) of 121 ± 16 s^−1^. Using these values, the calculated binding affinity for the nonhydrolyzable α,β-methylene ATP analogue (*K*_methT_) is 0.9 ± 0.2 μM.

Irreversible dissociation of α,β-methylene ATP was measured by competitive displacement with mant-AMP (Fig. 4*F*). Time courses of mant-AMP fluorescence enhancement after mixing a preequilibrated solution of ENPP1 (0.1 μM) and α,β-methylene ATP (20 μM) with excess mant-AMP (1 mM) follow double exponentials (Fig. 4*F*), with the fast phase reflecting dissociation of α,β-methylene ATP and the slow phase reflecting the second phase of mant-AMP binding (Fig. S2). The value of *λ*_obs,fast_ (*k*_−methT_) is 174 ± 10 s^−1^, roughly consistent with that obtained from binding kinetics and a *K*_methT_ of ∼ 1 μM. We assume that ATP also has a binding affinity (*K*_T_) of ∼ 1 μM.

mant-ATP binding to ENPP1 exhibits similar behaviors as ATP. Time courses of mant fluorescence enhancement follow double exponentials (Fig. S2*A*) with a fast phase observed rate constant (*λ*_obs,mT,fast_) that depends linearly on the [mant-ATP], yielding a second order mant-ATP association rate constant ($\frac{k_{+\text{mT}}}{K_{\text{mT},c}}$) of 58 ± 2 μM^−1^ s^−1^ and an intercept (*k*_+__mAMP′_ + *k*_−mAMP_) of 7 ± 3 s^−1^ (Fig. S2*B*). The observed rate constant of the slow phase (*λ*_obs,mT,slow_) depends weakly on the [mant-ATP] with an average value of 7 ± 0.5 s^−1^ (Fig. S2*B*). These values differ by < 2-fold from those of unlabeled ATP, consistent with the mant fluorophore modestly interfering with nucleotide (ATP and AMP) binding (15, 16, 17).

### AMP (product) binding to ENPP1

**Figure 5:**
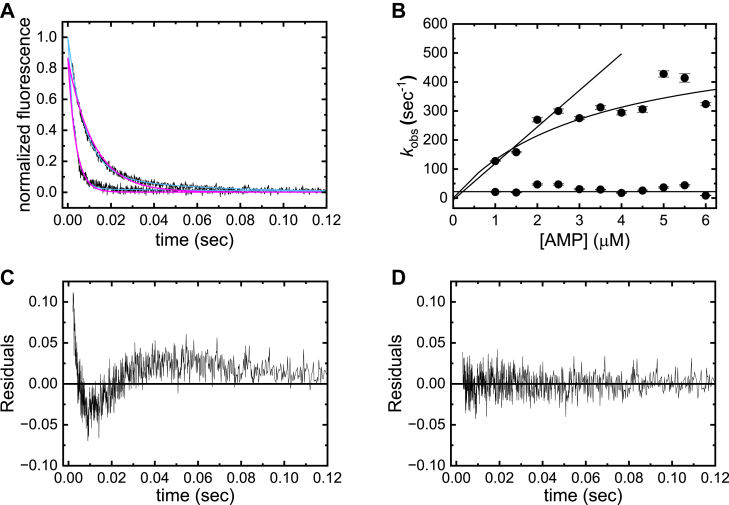
**AMP binding to ENPP1**. *A*, representative time courses of ENPP1 intrinsic tryptophan fluorescence decay after mixing ENPP1 (0.1 μM) with AMP (1–6 μM; 1 and 6 μM shown). *Smooth lines* through the data represent the best fits to a double exponential (*light blue*) and single exponential (*magenta*) functions. *B*, [AMP]-dependence of *λ*_obs,AMP,fast_ and *λ*_obs,AMP,slow_ from double exponential fits in *panelA*. *λ*_obs,AMP,fast_ varies hyperbolically with [AMP], yielding a maximum *λ*_obs,AMP,fast_ (*k*_+__AMP′_ +*k*_−AMP_ + *k*_+__AMP_) of 577 ± 102 s^−1^, a midpoint at [ATP] (*K*_AMP,c_) of 3 ± 3 μM, and an extrapolated intercept value that is indistinguishable from the slow phase relaxation. Fitting the first four data points to a linear function yields a slope ($\frac{k_{+AMP}}{K_{AMP,c}}$) of 126 ± 23 μM^−1^ s^−1^ and an intercept indistinguishable from the origin. *C*, residuals of the best fit of the 1 μM AMP trace to a single exponential. *D*, residuals of the best fit of the 1 μM AMP trace to a double exponential. ENPP1, ectonucleotide pyrophosphatase phosphodiesterase 1.

Time courses of ENPP1 (0.1 μM) intrinsic tryptophan fluorescence quenching after rapidly mixing with AMP (1–6 μM) fit to both single and double exponentials reasonably well (Fig. 5*A*). However, as observed for ATP binding, residuals of the best fits to a single exponential display a “ringing” characteristic of a poor fit of the data (Fig. 5*C*) and fitting to a double exponential yields a significantly better fit with randomly distributed residuals (Fig. 5*D*).

As with ATP, the fast phase monitors the formation of the ENPP1-bound AMP state (E∙M, Fig. 2) from a spectroscopically silent collision complex ((E∙M), Fig. 2). The fast observed rate constant (*λ*_obs,AMP,fast_) depends hyperbolically on [AMP] (Fig. 5*B*), and as defined by the microscopic rate constants according to (Equations 2 and S21):

(2) $$\lambda_{\text{obs},\text{AMP},\text{fast}}=k_{+A{\text{MP}}^{\prime }}+k_{-\text{AMP}}+\frac{k_{+\text{AMP}}\left[M\right]}{K_{{\text{AMP},}_{c}}+\left[M\right]}$$

The best fit of the data to a rectangular hyperbola yields a maximum *λ*_obs,AMP,fast_ value (*k*_+__AMP′_ + *k*_−AMP_ + *k*_+__AMP_) of 577 ± 102 s^−1^ and a midpoint (*K*_AMP,c_) of 3 ± 3 μM (Fig. 5*B*). The intercept value should be ∼*k*_+__AMP′_ + *k*_−AMP_, but is indistinguishable from the origin due to uncertainty (Fig. 5*B*), indicating the off-pathway isomerization (*k*_+__AMP′_) and AMP release (*k*_−AMP_) are slow, as observed with ATP binding. The best fit of a linear function to the first four data points yields a second-order AMP association rate constant ($\frac{k_{+\text{AMP}}}{K_{\text{AMP},c}}$) of 126 ± 23 μM^−1^ s^−1^ from the slope, indicating that *k*_+__AMP_ is rapid and dominates the maximum observed rate constant of AMP binding.

As with ATP binding, the slow phase monitors the off-pathway isomerization (*K*_AMP′_) of ENPP1 with noncovalently bound AMP (Fig. 2). The value of the slow observed rate constant (*λ*_obs,AMP,slow_) has an average value of 22 ± 3 s^−1^ and depends weakly on the [AMP] over the range examined. An off-pathway E.M′ state is necessary to account for the observed slow phases of nucleotide binding. The hyperbolic [AMP]-dependence of the fast phase indicates AMP binding follows a two-step mechanism, but these two states alone cannot account for the experimental data. The slow AMP binding phase indicates that an additional E.M state exists after two-step binding. Thus, three ENPP1∙AMP states exist. Numerous alternate schemes were considered and evaluated using KinTek Explorer Chemical Kinetics Software, but after an exhaustive attempt, only an off-pathway model, as shown in Figure 2, was consistent with all experimental data. We exclude a mechanism in which the slow phase represents the binding of a second nucleotide because the fluorescence change is independent of the [AMP].

mant-AMP binds ENPP1 following a similar mechanism as unlabeled AMP. Time courses of fluorescence enhancement follow double exponentials (Fig. S2*C*) with a fast phase observed rate constant (*λ*_obs,mAMP,fast_) that depends hyperbolically on the [mant-AMP], yielding a maximum value (*λ*_obs,mAMP,fast_ = *k*_+__mAMP′_ + *k*_−mAMP_ + *k*_+__mAMP_) of 595 ± 253 s^−1^, a midpoint (*K*_mAMP,c_) of 5 ± 5 μM, and an intercept (*k*_+__mAMP′_ + *k*_−mAMP_) indistinguishable from the origin (Fig. S2*D*). Fitting the first three data points to a linear function yields a second-order mant-AMP association rate constant ($\frac{k_{+\text{mAMP}}}{K_{\text{mAMP},c}}$) of 78 ± 8 μM^−1^ s^−1^ from the slope. The observed rate constant of the slow phase (*λ*_obs,mAMP,slow_) depends weakly on the [mant-AMP] with an average value of 9 ± 0.2 s^−1^ (Fig. S2*D*). These values differ by ≤ 2-fold from those of unlabeled AMP, consistent with the mant fluorophore modestly interfering with nucleotide (AMP) binding.

### Irreversible dissociation of AMP and mant-AMP from ENPP1

**Figure 6:**
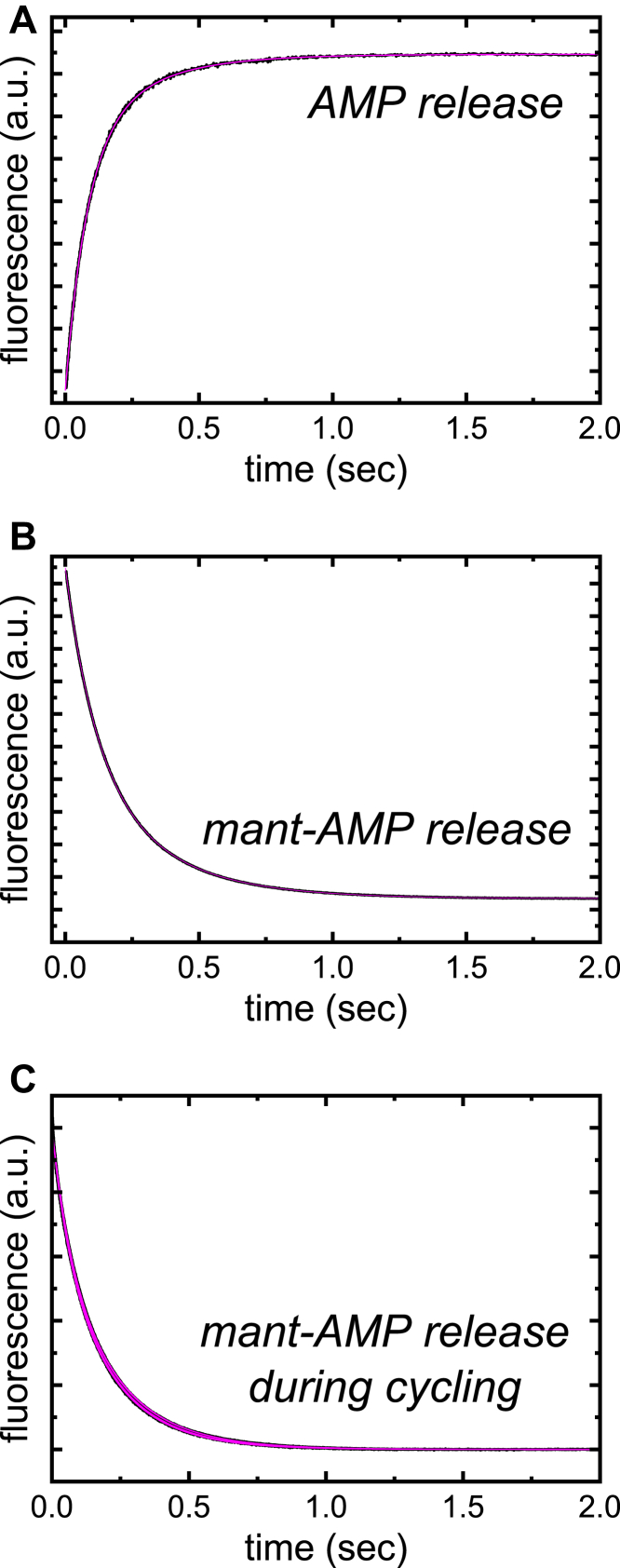
**Irreversible dissociation (competitive displacement) of AMP and mant-AMP from ENPP1**. *A*, time course of mant-AMP fluorescence enhancement after mixing a preequilibrated solution of ENPP1 (0.2 μM) and AMP (1 μM) with excess competing mant-AMP (60 μM). The *smooth line* through the data is the best fit to a double exponential with *λ*_obs,AMPdiss, fast_ and *λ*_obs,AMPdiss, slow_ of 12 ± 0.1 s^−1^ and 4 ± 0.04 s^−1^. Data shown is the average of seven individual time courses. *B*, time course of mant-AMP fluorescence decay after mixing a preequilibrated solution of ENPP1 (0.2 μM) and mant-AMP (2 μM) with excess competing AMP (50 μM). The *smooth line* through the data is the best fit to a double exponential with *λ*_obs,mAMPdiss, fast_ and *λ*_obs,mAMPdiss, slow_ of 9 ± 0.01 s^−1^ and 3 ± 0.004 s^−1^. Data shown are the average of six individual time courses. *C*, time courses of mant fluorescence decay after initially mixing ENPP1 (0.5 μM) with mant-ATP (15 μM) and then rapidly (within 0.028–0.5 s) mixing with excess competing AMP (1 mM). The *smooth lines* through the data are the best fits to a double exponential function with *λ*_obs,mAMPdiss, fast_ values ranging from 14 to 18 s^−1^ with a mean of 16 ± 0.4 s^−1^, and *λ*_obs,mAMPdiss, slow_ values ranging from 4.8 to 5.3 s^−1^ with a mean of 5.1 ± 0.02 s^−1^. All concentrations of competing nucleotide used in these experiments were saturating. ENPP1, ectonucleotide pyrophosphatase phosphodiesterase 1; mant-ATP, 2′/3′-O-(N-Methyl-anthraniloyl)-adenosine-5′-triphosphate; mant-AMP, 2′/3′-O-(N-Methyl-anthraniloyl)-adenosine-5′-monophosphate.

Time courses of fluorescence enhancement as mant-AMP competitively displaces unlabeled AMP following rapid mixing of a preequilibrated solution of ENPP1 (0.2 μM) and AMP (1 μM) with excess (60 μM) mant-AMP (Fig. 6*A*) fit well to double exponentials with observed rate constants of 12 ± 0.1 s^−1^ (*λ*_obs,AMPdiss,__fast_) and 4 ± 0.04 s^−1^ (*λ*_obs,AMPdiss,__slow_). The fast and slow observed rate constants represent the dissociation of AMP from both the E∙M and E∙M′ states and depend on the microscopic rate constants as follows (Equations 3 and 4; Equations S33 and S35):

(3) $$k_{+A{MP}^{\prime }}+k_{-A{MP}^{\prime }}+k_{-AMP}\;>\;\lambda_{obs,AMPdiss,fast}=12\;{\text{s}}^{-1}\;>\left\{ \begin{array}{c} k_{+{AMP}^{\prime }}+k_{-AMP},\text{if}\;k_{-AMP}>k_{-A{MP}^{\prime }}\\ k_{+A{MP}^{\prime }}+k_{-{AMP}^{\prime }},\text{if}\;k_{-AMP}<k_{-A{MP}^{\prime }} \end{array}\right.$$

(4) $$\left\{ \begin{array}{c} \frac{k_{-A{MP}^{\prime }}k_{-AMP}}{k_{+{AMP}^{\prime }}+k_{-AMP^{\prime }}},\text{if}\;k_{-AMP}<k_{-{AMP}^{\prime }}\\ \frac{k_{-A{MP}^{\prime }}k_{-AMP}}{k_{+A{MP}^{\prime }}+k_{-AMP}},\text{if}\;k_{-AMP}>k_{-A{MP}^{\prime }} \end{array}\right.\;>\;{\;\lambda }_{obs,AMPdiss,slow}=4\;{\text{s}}^{-1}\;>\;\frac{k_{-A{MP}^{\prime }}k_{-AMP}}{k_{+{AMP}^{\prime }}+k_{-A{MP}^{\prime }}+k_{-AMP}}$$

The complexity of the reaction scheme does not allow a unique solution for each rate constant to be determined from a single experiment, but constraints can be placed using measured parameters from multiple experiments: Equation 3 puts an upper limit of 12 s^−1^ on both *k*_−AMP_ and *k*_−AMP′_, and Equation 4 puts a lower limit of 4 s^−1^ on both *k*_−AMP_ and *k*_−AMP′_. With these constraints, Equation 3 then puts an upper limit of 8 s^−1^ on *k*_+__AMP′_.

The steady-state cycling parameters *K*_M,T_ and *k*_cat,T_ (Fig. 1) allow for additional constraints to be placed when they are expressed in terms of the microscopic rate constants outlined in Figure 2. *K*_M,T_ (Equations 5 and S42) is given by:

(5) $$K_{M,T}=\frac{k_{-A{MP}^{'}}k_{-AMP}K_{T,c}}{k_{+T}\left(k_{-A{MP}^{'}}+k_{+{AMP}^{'}}\right)+k_{-{AMP}^{'}}k_{-AMP}}=\frac{k_{-A{MP}^{'}}k_{-AMP}\frac{K_{T,c}}{k_{+T}}}{k_{-A{MP}^{'}}+k_{+{AMP}^{'}}+\frac{k_{-{AMP}^{'}}k_{-AMP}}{k_{+T}}}=\frac{k_{-AMP}\frac{K_{T,c}}{k_{+T}}}{1+\frac{k_{+{AMP}^{'}}}{k_{-A{MP}^{'}}}+\frac{k_{-AMP}}{k_{+T}}}=0.070\;\mu M$$

and introduces an additional inequality that places a lower limit of 6.8 s^−1^ on *k*_−AMP_:

(6) $${0.070\;\mu M\;<\;k}_{-AMP}\frac{K_{T,c}}{k_{+T}}\;\to \;0.070\;\mu M\;<\;\frac{k_{-AMP}}{97\;{\mu M}^{-1}\;{\text{s}}^{-1}}\;\to \;6.8\;\text{s}-1\;<\;k_{-AMP}$$

where the value of $\frac{K_{T,c}}{k_{+T}}=\frac{1}{97\;{\mu M}^{-1}\;{\text{s}}^{-1}}$ is obtained from the second-order ATP association rate constant (Fig. 4). Introducing this lower limit of 6.8 s^−1^ for *k*_−AMP_ into Equation 3 yields an upper limit of 5.2 s^−1^ for *k*_+__AMP__′_.

*k*_cat,T_ introduces an additional inequality (Equations 7 and S43) that constrains the equilibrium constant for off-pathway isomerization of the E∙M′ state (*K*_AMP′_ = *k*_+__AMP′_/*k*_−AMP′_).

(7) $$k_{cat,T}=\frac{k_{+T}k_{-{AMP}^{'}}k_{-AMP}}{k_{+T}\left(k_{-{AMP}^{'}}+k_{+{AMP}^{'}}\right)+k_{-A{MP}^{'}}k_{-AMP}}=\frac{k_{-AMP}}{1+\frac{k_{+{AMP}^{'}}}{k_{-{AMP}^{'}}}+\frac{k_{-AMP}}{k_{+T}}}\approx \frac{k_{-AMP}}{1+\frac{k_{+{AMP}^{'}}}{k_{-{AMP}^{'}}}}=3.3\;{\text{s}}^{-1}$$

The condition *k*_−AMP_ << *k*_+T_ is applied in the above equation.

(8) $$k_{-AMP}=3.3\;\left(1+\frac{k_{+{AMP}^{'}}}{k_{-{AMP}^{'}}}\right)\;\to \;3.3\;\left(1+\frac{k_{+{AMP}^{'}}}{k_{-{AMP}^{'}}}\right)\;>\;6.8\;\to \;1+\frac{k_{+{AMP}^{'}}}{k_{-{AMP}^{'}}}\;>\;2.1$$

(9) $$K_{AMP^{\prime }}=\frac{k_{+{AMP}^{\prime }}}{k_{-{AMP}^{\prime }}}\approx 1$$

Equation 9 indicates *k*_+__AMP′_ is comparable to *k*_−AMP′_, constraining the values of both isomerization rate constants to 4 to 5.2 s^−1^ (*i*.*e*. 4 < *k*_−AMP′_ ∼ *k*_+__AMP′_ < 5.2). Satisfying Equations (3), (4), (5), (6), yields the most likely values of *k*_−AMP_ ≈ 8 s^−1^, *k*_−AMP’_ ≈ *k*_+__AMP’_ ≈ 4 s^−1^.

We note that Equation 7 predicts the maximum steady-state turnover rate (*k*_cat,T_) is governed by AMP product release and the off-pathway isomerization. This behavior arises because all other rate constants in the ENPP1 ATPase cycle are rapid (>100 s^−1^; Table 1; further discussed below). Accordingly, the ENPP1 ATPase cycle is limited by both AMP product release (*k*_−AMP_) and isomerization to the off-pathway product-bound state (*K*_AMP′_).

Nearly identical results are obtained when competing mant-AMP (2 μM) from a preequilibrated ENPP1∙mant-AMP ([ENPP1] = 0.2 μM) complex with excess (50 μM) AMP (Fig. 6*B*). Time courses fluorescence decrease corresponding to mant-AMP dissociation also fit well to double exponentials with an *λ*_obs,mAMPdiss, fast_ and *λ*_obs,mAMPdiss, slow_ of 9 ± 0.01 s^−1^ and 3 ± 0.004 s^−1^. The observed rate constants of mant-labeled and unlabeled nucleotide dissociation from 0.2 μM ENPP1 differ by < 2-fold, consistent with the mant fluorophore modestly interfering with nucleotide (ATP and AMP) binding and suggesting the rate-limiting step is identical for the mant-labeled substrate.

The overall (noncovalent) AMP binding affinity (*K*_AMP, overall_) reflects contributions from both AMP-bound ENPP1 states (E∙M + E∙M′) according to (Equations 10 and S99):

(10) $$\begin{array}{c} K_{AMP,overall}=\frac{\left[E\right]\left[M\right]}{\left[EM\right]+\left[EM^{\prime }\right]}=K_{M,c}\frac{K_{AMP}K_{AMP^{\prime }}}{K_{AMP^{\prime }}+1} \end{array}$$

Using the experimentally determined rate and equilibrium constants, we calculate an overall AMP binding affinity of ∼21 nM, tighter than the ∼100 nM value obtained by surface plasmon resonance (24).

Because AMP binds with an affinity that is slightly tighter than *K*_M,T_, ENPP1 is subject to product inhibition when [ATP] and [AMP] are comparable. Consistent with this prediction, the ENPP1 steady state ATPase rate decreases by a factor of two when the initial concentrations of ATP and AMP are equal (Fig. S4).

### Irreversible dissociation of mant-AMP from ENPP1 during ATPase cycling

ENPP1 (0.5 μM) was initially mixed with mant-ATP (15 μM) under multiturnover conditions ([mant-ATP] >>[ENPP1]) and the mant-AMP product was subsequently competed off at various time points during cycling (0.028–0.5 s) by mixing with excess AMP (1 mM). Time courses of mant fluorescence decay (corresponding to mant-AMP release) at all aging times follow nearly identical double exponentials with *λ*_obs,AMPdiss, fast_ values ranging from 14 to 18 s^−1^ with a mean of 16 ± 0.4 s^−1^, and *λ*_obs,AMPdiss, slow_ values ranging from 4.8 to 5.3 with a mean of 5.1 ± 0.02 s^−1^ (Fig. 6C).

These dissociation rate constants differ by < 2-fold from those observed when competing mant-AMP or AMP from a preequilibrated complex (Fig. 6, A and B), suggesting the two noncovalent (mant-)AMP-bound ENPP1 states (E∙M and E∙M′; Fig. 2) are populated almost immediately following (mant-)ATP binding (*λ*_obs,mT,fast_ ≈900 s^−1^ under these conditions). This observation implies all biochemical transitions between the (mant-)ATP-bound (E∙T; Fig. 2) and the (mant-)AMP-bound (E∙M; Fig. 2) ENPP1 states (*i*.*e*., formation of the covalent ENPP1-(mant-)AMP intermediate, pyrophosphate (PP_*i*_) release, and ENPP1-(mant-)AMP hydrolysis; *K*_internal_; Fig. 2) are complete within 30 milliseconds of mixing, and supports a mechanism in which ENPP1 predominantly populates the AMP-bound states (E∙M or E∙M′; Fig. 2) during steady-state cycling ([E∙T] ≈0).

### ATP single and multiturnover by ENPP1–direct measurement of chemical cleavage by quenched-flow

**Figure 7:**
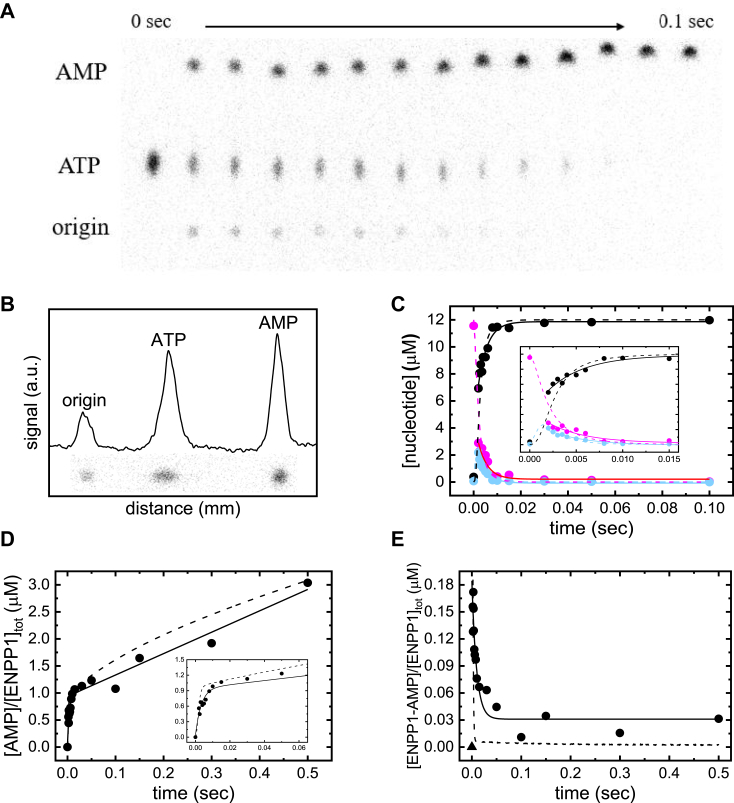
**Direct measurement of single and multiturnover ATP hydrolysis–quenched-flow**. *A*, TLC plate showing the separation of [α-^32^P]ATP, [α-^32^P]AMP and [α-^32^P]AMP−ENPP1 (stuck at the origin) in individual reaction samples quenched at time points ranging from 0 to 0.1 s (data shown in *panelC*). *B*, representative chromatogram generated from a single TLC plate lane (0.003 s time point). Integrated peak areas were used to quantify nucleotide species at each time point. *C*, time courses of ATP depletion (*magenta circles*), AMP production (*black circles*), and ENPP1-AMP (*light blue circles*) production and depletion after mixing ENPP1 (15 μM final) with ATP (12 μM final, with trace [α-^32^P]ATP). *Solid lines* through the data represent the best global fits to a single exponential function with a shared *λ*_obs_ of 254 ± 26 s^−1^. *Dashed lines* in panels *C*–*E* represent kinetic simulations of Figure 2 with rate and equilibrium constants defined in Table 2. *D*, time course of AMP accumulation (noncovalently bound and free) after mixing ENPP1 (4 μM final) with ATP (60 μM final, trace [α-^32^P]ATP). The *solid lines* represent the best fit of the data to an exponential plus a linear function, yielding an observed rate of 295 ± 76 s^−1^ for the initial burst phase of AMP production, and a steady state rate of 4.0 ± 0.3 s^−1^, corresponding to an equivalent turnover number (*k*_cat,T_) because ATP is saturating ([ATP]>>*K*_M,T_; Fig. 1, Table 1). *E*, time course of ENPP1−AMP covalent complex depletion after mixing ENPP1 (4 μM final) with ATP (60 μM final, trace α-^32^P). Formation of covalent intermediate is too rapid to capture. The *solid line* through the data represents the best fit to a single exponential function, yielding an observed decay rate of 105 ± 27 s^−1^, and a nonzero *y*_0_ value of 0.03 ± 0.01 ENPP1−AMP site^−1^ during steady state. ENPP1, ectonucleotide pyrophosphatase phosphodiesterase 1.

Time courses of ATP (*i*.*e*., T + E∙T; Fig. 2) depletion, AMP (*i*.*e*., E∙M + E∙M′ + M; Fig. 2) production, and ENPP1−AMP (*i*.*e*., E−M∙PP_*i*_ + E−M; Fig. 2) depletion were monitored by chemical quenched-flow and TLC with [α-^32^P]ATP under single-turnover conditions ([ENNP1] = 15 μM > [ATP] = 12 μM; Fig. 7, *A–C*). Globally fitting the time courses for all three populations to a single exponential with a shared rate constant (*λ*_obs_) yields an *λ*_obs_ of 254 ± 26 s^−1^ (Fig. 7*C*).

All ATP is converted to AMP (E∙M + E∙M′ + M) within 15 milliseconds (Fig. 7*C*), supporting our conclusion from (mant-) AMP dissociation during cycling (Fig. 6) that all biochemical transitions of the ENPP1 ATPase cycle preceding the E∙M state (Fig. 2) proceed rapidly, and that the ENPP1 ATPase cycle is limited by AMP product release (*k*_−AMP_) and isomerization (*K*_AMP′_). The nucleotidylated ENPP1−AMP intermediate is completely depleted, indicating that AMP product formation is heavily favored (*K*_hydrolysis_ >10; Fig. 2), and hydrolysis of this intermediate (step 4; Fig. 2) is essentially irreversible (*k*_−hydrolysis_ ≈ 0; Fig. 2). Previous studies demonstrate that ENPP1 does not form adenosine or ADP from ATP (10). The detection of only AMP product over this rapid time scale is consistent with a catalytic mechanism involving nucleophilic attack of the α-phosphate (2).

Time courses of total AMP production (*i*.*e*., E∙M + E∙M′ + M; Fig. 2) using the same experimental procedures above under multiturnover conditions ([ATP] = 60 μM >> [ENPP1] = 4 μM) exhibit a single exponential burst phase with an *λ*_obs_ of 295 ± 76 s^−1^ followed by a linear (steady-state) phase with a velocity (*v*_0_ = *k*_cat,T_) of 4.0 ± 0.3 s^−1^ per enzyme (Fig. 7*D*). The presence of a burst phase of AMP production before reaching steady state confirms the rate-limiting step of the ENPP1 ATPase occurs after chemical cleavage of the ENPP1−AMP covalent intermediate (25). The burst phase amplitude is nearly one (0.9 ± 0.1) AMP per enzyme, indicating that product formation is heavily favored. The near unity magnitude of the burst-phase amplitude also indicates that the forward rate constants for all biochemical transitions preceding the E∙M state are significantly faster than both AMP product release (*k*_−AMP_) and E∙M isomerization (*K*_AMP′_; Fig. 2), as concluded from irreversible dissociation of (mant-)AMP during steady-state cycling (Fig. 6*C*).

## Discussion

### ENPP1-catalyzed liberation of PP*_i_* from ATP

ENPP1 catalyzes the liberation of PP_*i*_ from ATP with a maximum turnover rate (*k*_cat,T_) of 3.3 ± 0.2 s^−1^ and a *K*_M,T_ of 70 ± 23 nM (Fig. 1), consistent with previous determinations obtained under similar solution conditions (9). Kato *et al*. (13) reported a *k*_cat,T_ of 16 s^−1^ and a *K*_M,T_ of 46 μM for the ENPP1 ATPase, but these were determined using a single-time point assay, and different solution conditions and temperature (37 °C in a buffer containing 100 mM Tris–HCl (pH 9.0), 500 mM NaCl, and 5 mM MgCl2) (26). These differences in temperature, pH, salt, and the lack of Zn^2+^, a critical component of the ENPP1 active site, may account for this difference. We also note that these studies were carried out with a soluble form of ENPP1, and membrane association of the full-length protein could potentially affect many of the enzymatic parameters determined with the isolated extracellular domain.

**Figure 8:**
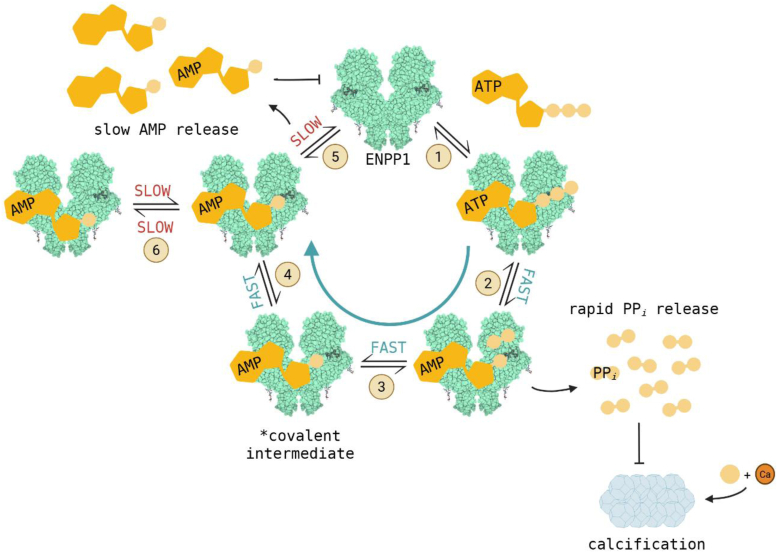
**Illustrative schematic of the ENPP1 ATPase cycle**. A simplified version of the ENPP1 ATPase scheme highlighting the key features of the cycle: rapid chemistry and PP_*i*_ product release with rate limiting AMP product release. Transition 1 is representative of formation of the stably bound E∙ATP state through the formation of an initial collision complex ((E∙ATP); Fig. 2), so that the observed forward rate constant is *k*_+__T_/*K*_T,c_. Transitions 2 to 4 represent rapid ATP cleavage & formation of the nucleotidylated E−AMP intermediate (*K*_cleavage_), PP_*i*_ release (*K*_PPi_), and E−AMP hydrolysis to form the E∙AMP state (*K*_hydrolysis_). Transition 5, like transition 1, represents formation of the E∙AMP collision complex and the stably bound E∙AMP state, so that the observed forward rate constant for AMP binding is *k*_+__AMP_/*K*_AMP,c_. Transition 6 represents isomerization to the off-pathway E∙AMP′ state (*K*_AMP′_). The solid green arrow highlights the fact that transitions 2 to 4 occur so rapidly that ATP is essentially instantaneously converted to AMP upon binding. Isomerization to the off-pathway E∙AMP′ state prolongs the lifetime of the AMP product-bound states, contributing to slow overall product release and limiting the rate of cycling. Created in BioRender. Michalchik, M. (2025) https://BioRender.com/e92o051. ENPP1, ectonucleotide pyrophosphatase phosphodiesterase 1.

Transient kinetic analysis indicates the maximum ATPase turnover rate of ENPP1 is limited by the slow release of AMP product following rapid ATP binding and cleavage, PP_*i*_ release, and hydrolysis of the nucleotidylated ENPP1−AMP intermediate (Fig. 2; Fig. 8). Chemical quenched-flow experiments under single-turnover conditions ([ENPP1] = 15 μM, [ATP] = 12 μM) reveal that the total conversion of ATP to AMP is complete within ∼15 milliseconds (*k*_+__cleavage_, *k*_−PP*i*_, *k*_+__hydrolysis_ > 1000 s^−^^1^). PP_*i*_ binds weakly (*K*_PP*i*_ >> 100 μM; Fig. S4), and its dissociation can be considered essentially irreversible. Rate-limiting AMP product release is evidenced by the existence of a burst phase in AMP product production under multiturnover conditions ([ATP] >> [ENPP1]; Fig. 7*D*). The hydrolysis of the nucleotidylated E−M state is irreversible, as evidenced by the complete depletion of this species under single-turnover conditions (Fig. 7). We note that although we have presented all transitions between the E∙T and E∙M states in a distinct order, this is by analogy to the mechanism of the related enzyme AP (10, 11, 13). Our data cannot conclusively distinguish between the order of events or if additional intermediates exist.

The low (*i*.*e*. tight) *K*_M,T_ value originates from rapid ATP cleavage, PP_*i*_ product release, and covalent intermediate (ENPP1−AMP) hydrolysis steps following ATP binding (Table 1). *K*_M,T_ is comparable to the physiological [ATP] in blood (27, 28), so ENPP1 is running at approximately half of its maximum velocity (Fig. 1*C*) under this nonsaturating [ATP]. Accordingly, the rate of ENPP1-catalyzed PP_*i*_ liberation is sensitive to small changes in [ATP], thus allowing ENPP1 to sense and respond to the local [ATP] and any transient fluctuations. The second-order association rate constant for ATP binding is extremely rapid (*k*_+__T_/*K*_T,c_ ≈ 100 μM^−1^ s^−1^; Table 1), but within the diffusion limit (29). The specificity constant for ATP (*k*_cat,T_/*K*_M,T_ ≈ 50 μM^−1^ s^−1^), often referred to as the apparent second-order substrate association rate constant, is within a factor of two of the ATP binding rate constant, indicating that approximately half of productive ATP binding events progress forward through the catalytic cycle. Although there is some uncertainty in these estimates, it is consistent with ENPP1 being an efficient enzyme that has adapted to rapidly bind and catalyze the hydrolysis of ATP, even at low concentrations such as those found in serum.

AMP release limits the ENPP1 ATPase. All other biochemical transitions are far more rapid, so the predominant intermediates populated during steady state cycling are those with AMP bound noncovalently. There is little structural difference between apo and AMP-bound ENPP1, so we assume the slow release of the AMP product originates from specific interactions with the nucleotide base (24, 26). We note that only one structural state of ENPP1 with noncovalently bound AMP has been observed (24, 26), despite there being two biochemical states. Since the signal identifying these two states is spectroscopic, structural differences could be very subtle and still yield a fluorescence change.

**Figure 9:**
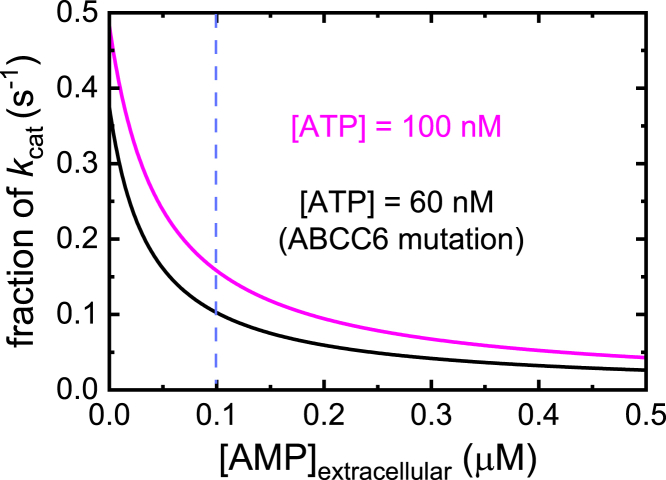
**ENPP1 enzymatic activity under physiological and pathophysiological conditions**. Fractional activity of ENPP1 ATPase at physiological (100 nM, *magenta solid line*) and pathophysiological (60 nM, *black solid line*) serum [ATP] as a function of competing [AMP]. The ENPP1 ATPase rate was determined using the competitive inhibition equation of steady-state enzyme kinetics (14), the indicated ATP and AMP concentrations, a *K*_M,T_ of 70 nM, and a *K*_AMP, overall_ of 21 nM. The *dashed vertical line* corresponds to the average physiological [AMP] concentration in serum. ENPP1, ectonucleotide pyrophosphatase phosphodiesterase 1.

AMP also binds rapidly (Table 1) and with a tight binding affinity (*K*_AMP__,overall_ ≈ 0.021 μM), rendering ENPP1 susceptible to competitive inhibition by AMP product (Figs. 8 and 9). Under single-turnover conditions, we see that AMP product remains bound to ENPP1 following hydrolysis and occupies one of the two noncovalent AMP-bound states (E∙M or E∙M′, Fig. 2; Fig. 3), suggesting the presence of ATP is required to outcompete tightly bound AMP product from the E∙M state for subsequent cycles of catalysis to occur. The magnitude of the rate constants for AMP dissociation from the E∙M state and internal conversion to the E∙M′ state are similar (*k*_−AMP_ ≈ 8 s^−1^; *k*_−AMP′_ ≈ 4 s^−1^), so that both biochemical transitions contribute to the rate-limiting dissociation of AMP product. Such off-pathway states have been identified in muscle (30) and nonmuscle myosins (15), and have been implicated in prolonging the lifetime of the product-bound states.

Because AMP binds ENPP1 so tightly, submicromolar concentrations can effectively compete with ATP substrate (Fig. S4), leading to inhibition of catalytic activity (Fig. S4). Under physiological nucleotide concentrations of ∼100 nM ATP and ∼100 nM AMP, ENPP1 is running at less than 20% of its maximum turnover rate (Fig. 9). AMP product inhibition can therefore serve as an intrinsic regulatory mechanism controlling overall PP_*i*_ liberation, allowing ENPP1 to tune its catalytic activity. AMP generated from rapid bursts of ENPP1 activity will feedback inhibit ENPP1, thereby modulating its activity and maintaining plasma [PP_*i*_] at relatively constant levels independent of [ENPP1] or [ATP].

### Implications for enzyme replacement therapy

ENPP1 is the only human enzyme responsible for the generation of systemic plasma PP_*i*_ as documented by the near absence of plasma PP_*i*_ in individuals with bi-allelic ENPP1 deficiency (4). Mammalian ENPP1 is therefore the primary enzyme responsible for systemic levels of PP_*i*_ and AMP, deficiencies of which induces arterial calcifications and vascular stenoses, respectively (31). Moreover, murine models of ENPP1 deficiency demonstrate that ENPP1 catalysis also suppresses the phosphaturic hormone FGF23 (32), which constitutes the pathogenic mechanism of ARHR2. Subcutaneous administration of the ENPP1-based biologic normalizes plasma PP_*i*_ levels in patients with both ENPP1 and ABCC6 deficiency, but with a dose dependence unique to the underlying genetic cause (https://investors.inozyme.com/events-presentations). In patients with ENPP1 deficiency, ENPP1 administration restores and maintains patient plasma PP_*i*_ levels over the range of administered doses, while in those with ABCC6 deficiency only high doses of ENPP1 biologic restore plasma [PP_*i*_] to physiological levels. The quantitative enzymatic analysis presented here provides a biochemical explanation for the observed dose dependence in each circumstance.

ENPP1 deficiency causes systemically low PP_*i*_ because the enzymatic activity required to liberate PP_*i*_ from ATP is compromised. Reintroduction of ENPP1 catalytic activity *via* ERT can therefore recover PP_*i*_ liberation and regulation of plasma [PP_*i*_]. The intrinsic regulatory mechanism of AMP feedback inhibition modulates ENPP1 activity, ensuring that even over a broad range of administered ENPP1 therapeutic in ERT, PP_*i*_ production will not exceed what is required to maintain normal plasma [PP_i_]. Although the intuitive prediction based on a textbook understanding of steady-state kinetics would suggest higher ERT doses should produce higher levels of PP_*i*_ (*i*.*e*. product formation scales linearly with [enzyme]; (14)), knowledge of the detailed biochemical reactions underlying the catalytic ENPP1 ATPase cycle favors a mechanism in which ENPP1 has adapted enzymatic properties, such as product inhibition, that allow it to serve as a physiological “PP_*i*_ buffer”.

ABCC6-deficient patients have functional copies of ENPP1, but a modest (<2-fold) reduction in circulating [ATP] (33). This systemic reduction in ATP is sufficient to lower [PP_*i*_] and cause GACI (34). This can be explained because at physiological [ATP] (∼0.1 μM) ENPP1 is operating at half its maximal speed (Fig. 1*C*), so even a small reduction in [ATP] can have a dramatic impact on the rate of PP_*i*_ production (Fig. 9). The rate of PP*i* liberation scales linearly with [ENPP1] according to Equation 12, so supplementing ENPP1 above basal levels with ERT increases the rate of PP_*i*_ production. The additional ENPP1 must compensate for the slower PP_*i*_ production rate arising from the lower [ATP]. Accordingly, the lowest ENPP1 doses evaluated in ERT were sufficient for treating ENPP1 deficiency but inadequate in treating ABCC6-deficient patients. Higher ERT doses are needed to counteract the lower substrate availability.

This detailed understanding of ENPP1 enzymology allows us to make plausible predictions for other PP_*i*_-linked calcification disorders. Because of the prominent AMP product inhibition (Figs. 8 and 9), compromised degradation and clearance of serum AMP is predicted to inhibit ENPP1, reduce systemic PP_*i*_ levels, and generate aberrant calcification. Consistent with this biochemical prediction, inactivating mutations to the nucleotidase CD73, which degrades serum AMP to maintain a steady state level of ∼100 nM, are associated with a peripheral arterial calcification disorder called arterial calcification due to deficiency of CD73 (ACDC; (35)).

## Experimental procedures

### Protein and reagents

All reagents were the highest purity commercially available. The fluorescent nucleotide analogues, mant-ATP and mant-AMP, were purchased from Jena Biosciences or BIOLOG Life Science Institute. Radiolabeled ATP (α-^32^P) was purchased from Revvity. Glycosylated human ENPP1-Fc ((9) and construct 770 in ref. (36), consisting of the human ENPP7 signal sequence for export, the somatomedin B 1&2, catalytic, and endonuclease domains of human ENPP1, followed by the human IgG1 Fc domain) was purified from CHO cells as described (36), dialyzed against PBS_plus_ buffer (1X PBS buffer pH 7.4, 11 μM ZnCl_2_, and 20 μM CaCl_2_) and stored at −80 ° C. All experimental measurements were carried out at 25 °C in ENPP1 assay buffer designed to mimic salt concentrations in blood (20 mM Tris–HCl (pH 7.4), 154 mM NaCl, 0.014 mM ZnCl_2_, 1 mM MgCl_2_, 1 mM CaCl_2_, and 4.5 mM KCl). ENPP1 and nucleotide concentrations stated are final after mixing, unless specified otherwise.

### Chemical cleavage of ATP

Chemical cleavage of ATP was measured by rapidly mixing ENPP1 and ATP (containing trace [α-^32^P]ATP) using a KinTek RQF-3 Chemical-Quench-Flow apparatus, quenching with 4.5 M formic acid at the indicated times and subsequently quantifying nucleotide species in solution. Quenched samples (1–3 μl) were spotted onto PEI-Cellulose TLC plates (MilliporeSigma) and resolved in 0.6 M K_2_HPO_4_ (pH 3.4) for 25 to 30 min. Plates were exposed to a storage phosphor screen and scanned by an FLA-5100 fluorescent image analyzer (Fujifilm). Multi Gauge V3.0 Image Analysis software (Fujifilm) was used to quantify substrate (ATP), covalent intermediate (ENPP1-AMP) and product (AMP) concentrations. Signal intensities of individual lanes were transformed into chromatograms. Integrated areas for each peak were used to quantify all nucleotide species. Reactions were carried out under multiturnover ([ENPP1] = 4 μM and [ATP] = 60 μM or [ENPP1] = 20 nM and [ATP] = 0.1 to 10 μM) and single-turnover ([ENPP1] = 15 μM, [ATP] = 12 μM) conditions. The quenching method utilized here enables the simultaneous quantification of both free and noncovalently bound nucleotide in solution, and therefore directly monitors hydrolysis of both ATP and the nucleotidylated ENPP1−AMP intermediate.

### Chemical cleavage of mant-ATP and α,β-methylene ATP

Chemical cleavage of mant-ATP or α,β-methylene ATP was monitored (nonreal-time) by HPLC separation of substrate (ATP) and product (AMP) in reaction samples taken at various time points. The reaction was initiated by manually mixing a small volume of concentrated enzyme stock with solutions of ATP and quenched at indicated time points by removing an aliquot and rapidly mixing with cold 3 M formic acid. The reverse phase HPLC program for nucleotide analysis consists of an initial 1 min of sample loading with Buffer A (15 mM ammonium acetate solution, pH 6.0) at a flow rate of 0.1 ml/minute, followed by 8 min of washing with Buffer A, 11 min of gradient with 0 to 100% Buffer B (15 mM ammonium acetate solution, pH 6.0, 20% (v/v) methanol), 10 min of column cleaning with Buffer B, and 10 min of column reequilibrating for the next sample with Buffer A at a flow rate of 1 ml/min. Nucleotides are detected by UV absorption at 259 nm. Peak identities in absorption chromatograms are determined by nucleotide standards in the same reaction buffer and quenching reagent, under the same running conditions as reaction samples. The nucleotide quantification is carried out by converting the integration of the corresponding UV absorption peak to nucleotide concentration according to a standard integrated peak area versus concentration curve.

### “Steady-state” kinetics

Time courses of ENPP1-catalyzed ATP hydrolysis were measured with 20 nM ENPP1 and a range (0.1–10 μM) of [ATP]. AMP or PP_*i*_ were included where indicated in the text. Time courses of AMP product ([*P*]) formation were fitted to:

(11) $$\left[P\right]=\frac{v_{0}}{\eta }\left(1-e^{-\eta t}\right)$$

where *v*_0_ is the initial velocity of AMP product production, *η* is the decay constant of the steady-state rate (*i*.*e*., the first derivative of the turnover velocity) from substrate depletion and/or product inhibition, and *t* is time (12). The Michaelis constant for ATP (*K*_M,ATP_) and the maximum ENPP1 turnover rate constant (*k*_cat_) were obtained by fitting the [ATP]-dependence of the initial velocity (*v*_0_) to a rectangular hyperbola in the form of the familiar Briggs-Haldane equation:

(12) $$v_{0}=\frac{k_{\text{cat},T}\left[ENPP1\right]\left[T\right]}{\left[T\right]+K_{M,T}}$$

Nucleotide binding kinetics–Time courses of fluorescence intensity change were recorded after rapidly mixing ENPP1 with nucleotide using an Applied Photophysics SX.20MV-R stopped-flow apparatus. Unlabeled nucleotide (ATP, ADP, and AMP) binding was monitored from changes in intrinsic ENPP1 tryptophan fluorescence (*λ*_ex_ = 280 nm, emission measured through a 299 nm or WG320 long pass colored glass filter, with similar results). mant-labeled nucleotide binding and dissociation were monitored by FRET from ENPP1 tryptophan residues to the mant moiety of the nucleotide analogues (*λ*_ex_ = 280 nm, emission measured through a 400 nm long pass colored glass filter). Inner filter effects are negligible at nucleotide concentrations used. Time courses presented are averages of at least three traces.

Time courses of fluorescence change were fitted to a sum of exponentials,

(13) $$F\left(t\right)=F_{\infty }+\sum_{i=1}^{n}A_{i}e^{-k_{i}t}$$

where *F*(*t*) is the fluorescence at time *t*, *F*_*∞*_ is the final fluorescence intensity, *A*_*i*_ is the amplitude, *k*_*i*_ is the *λ*_obs_ characterizing the *i*^*th*^ relaxation process, and *n* is the total number of observed relaxations (21). The value of *n* was either one (single exponential) or two (double exponential). The dead time of the instrument determined from the reduction of 2,6-dichlorophenolindophenol with ascorbic acid in absorbance mode was 0.002 s. Fitting was limited to data beyond 0.002 to 0.003 s to account for the instrument dead time and to exclude data acquired during the continuous flow phase of mixing as recommended by the manufacturer.

Uncertainties are reported as standard errors in the fits unless stated otherwise and were propagated using the general formula:

(14) $$da=\sqrt{{\left(\frac{\partial_{a}}{\partial x_{1}}dx_{1}\right)}^{2}+\cdots +{\left(\frac{\partial_{a}}{\partial x_{n}}dx_{n}\right)}^{2}}$$

where the experimental measurements *x*_1_, *x*_2_ … *x*_n_ have uncertainties *dx*_1_, *dx*_2_ … *dx*_n_ and *a* is a function of *x*_1_, *x*_2_ … *x*_n_. For long time courses (2–100 s), traces were background corrected for photobleaching by subtracting the time course of fluorescence intensity of ENPP1 alone.

### AMP and mant-AMP product release

mant-AMP dissociation from ENPP1 (*i*.*e*. ENPP1-mant-AMP) was measured in two different ways: (a) from a preequilibrated sample of mant-AMP and ENPP1 and (b) from ENPP1 undergoing a catalytic turnover with mant-ATP substrate. Dissociation of ENPP1-mant-AMP was measured by rapidly mixing a preequilibrated solution of ENPP1 and mant-AMP with a range of unlabeled AMP. Final concentrations after mixing were 0.5 μM ENPP1, 0.05 μM mant-AMP, and 0.25 μM-1 mM AMP. Irreversible dissociation of mant-AMP following a single catalytic event with mant-ATP as a substrate was measured in sequential mixing mode. ENPP1 (2 μM) was rapidly mixed with mant-ATP (120 μM) and allowed to equilibrate for approximately 30 milliseconds before rapidly mixing with excess unlabeled AMP (2 mM) to prevent mant-ATP and mant-AMP rebinding. Final concentrations were 0.5 μM ENPP1, 30 μM mant-AMP, and 1 mM AMP.

AMP release was measured by competition in a similar manner: preequilibrated ENPP1 and AMP samples were rapidly mixed with excess mant-AMP. mant-AMP binding is limited by AMP dissociation and therefore reports on the AMP release rate constant.

### Kinetic simulations, fitting, and modeling

Data analysis and fitting was done using Origin 2023 (OriginLab). Individual rate constants were determined using analytical solutions of differential equations (Supplementary Information). In cases where unique solutions were unattainable and rate constants could not be precisely determined, boundary limits were placed on their value using available data. Kinetic simulations were performed according to Figure 2 using KinTek Explorer Chemical Kinetics Software (KinTek Co).

## Data availability

All data are presented in the manuscript figures and/or Online Supplementary Material. Raw data and analysis done will be shared as an Origin software file upon request by emailing the corresponding author.

## Supporting information

This article contains supporting information.

## Conflict of interest

DTB is an inventor on patents owned by Yale University for therapeutics treating ENPP1 deficiency, and an equity holder who receives research and consulting support from Inozyme Pharma. EMDLC is an equity holder in Inozyme Pharma.

## References

[1] Borza R., Salgado-Polo F., Moolenaar W.H., Perrakis A. (2022). Structure and function of the ecto-nucleotide pyrophosphatase/phosphodiesterase (ENPP) family: tidying up diversity. J. Biol. Chem..

[2] Caswell A.M., Russell R.G.G. (1988). Evidence that ecto-nucleoside-triphosphate pyrophosphatase serves in the generation of extracellular inorganic pyrophosphate in human-bone and articular-cartilage. Biochim. Biophys. Acta..

[3] Rutsch F., Ruf N., Vaingankar S., Toliat M.R., Suk A., Höhne W. (2003). Mutations in are associated with 'idiopathic' infantile arterial calcification. Nat. Genet..

[4] Rutsch F., Vaingankar S., Johnson K., Goldfine I., Maddux B., Schauerte P. (2001). PC-1 nucleoside triphosphate pyrophosphohydrolase deficiency in idiopathic infantile arterial calcification. Am. J. Pathol..

[5] Ferreira C.R., Kintzinger K., Hackbarth M.E., Botschen U., Nitschke Y., Mughal M.Z. (2021). Ectopic calcification and hypophosphatemic rickets: natural history of ENPP1 and ABCC6 deficiencies. J. Bone. Miner. Res..

[6] Levy-Litan V., Hershkovitz E., Avizov L., Leventhal N., Bercovich D., Chalifa-Caspi V. (2010). Autosomal-recessive hypophosphatemic rickets is associated with an inactivation mutation in the ENPP1 gene. Am. J. Hum. Genet..

[7] Lorenz-Depiereux B., Schnabel D., Tiosano D., Hausler G., Strom T.M. (2010). Loss-of-function ENPP1 mutations cause both generalized arterial calcification of infancy and autosomal-recessive hypophosphatemic rickets. Am. J. Hum. Genet..

[8] O'Brien C., Khursigara G., Huertas P., Leiro B., Molloy L., Nester C. (2022). Lifelong impact of ENPP1 deficiency and the early onset form of ABCC6 deficiency from patient or caregiver perspective. PLoS One.

[9] Albright R.A., Stabach P., Cao W.X., Kavanagh D., Mullen I., Braddock A.A. (2015). ENPP1-Fc prevents mortality and vascular calcifications in rodent model of generalized arterial calcification of infancy. Nat. Commun..

[10] Albright R.A., Ornstein D.L., Cao W.X., Chang W.C., Robert D., Tehan M. (2014). Molecular basis of purinergic signal metabolism by ectonucleotide Pyrophosphatase/Phosphodiesterases 4 and 1 and implications in stroke. J. Biol. Chem..

[11] Zalatan J.G., Fenn T.D., Brunger A.T., Herschlag D. (2006). Structural and functional comparisons of nucleotide pyrophosphatase/phosphodiesterase and alkaline phosphatase: implications for mechanism and evolution. Biochemistry.

[12] Cao W.X., De La Cruz E.M. (2013). Quantitative full time course analysis of nonlinear enzyme cycling kinetics. Sci. Rep..

[13] Gijsbers R., Ceulemans H., Stalmans W., Bollen M. (2001). Structural and catalytic similarities between nucleotide pyrophosphatases/phosphodiesterases and alkaline phosphatases. J. Biol. Chem..

[14] Johnson K.A. (2019). Kinetic Analysis for the New Enzymology: Using Computer Simulation to Learn Kinetics and Solve Mechanisms.

[15] Hannemann D.E., Cao W.X., Olivares A.O., Robblee J.P., De La Cruz E.M. (2005). Magnesium, ADP, and actin binding linkage of myosin V: evidence for multiple myosin V-ADP and actomyosin V-ADP states. Biochemistry.

[16] Moore K.J.M., Lohman T.M. (1994). Kinetic mechanism of adenine-nucleotide binding to and hydrolysis by the Escherichia-Coli rep monomer .1. use of fluorescent nucleotide analogs. Biochemistry.

[17] Talavera M.A., De La Cruz E.M. (2005). Equilibrium and kinetic analysis of nucleotide binding to the DEAD-box RNA helicase DbpA. Biochemistry.

[18] Wong E.V., Cao W.X., Vörös J., Merchant M., Modis Y., Hackney D.D. (2016). Pi release limits the intrinsic and RNA-stimulated ATPase cycles of DEAD-box protein 5 (Dbp5). J. Mol. Biol..

[19] Bale J.R., Huang C.Y., Chock P.B. (1980). Transient kinetic-analysis of the catalytic cycle of alkaline-phosphatase. J. Biol. Chem..

[20] Robblee J.P., Olivares A.O., De la Cruz E.M. (2004). Mechanism of nucleotide binding to actomyosin VI - Evidence for allosteric head-head communication. J. Biol. Chem..

[21] De La Cruz E.M., Ostap E.M. (2009). Kinetic and equilibrium analysis of the myosin atpase. Methods. Enzymol..

[22] Pollard T.D., De La Cruz E.M. (2013). Take advantage of time in your experiments: a guide to simple, informative kinetics assays. Mol. Biol. Cell..

[23] Robblee J.P., Cao W.X., Henn A., Hannemann D.E., De La Cruz E.M. (2005). Thermodynamics of nucleotide binding to actomyosin V and VI: a positive heat capacity change accompanies strong ADP binding. Biochemistry.

[24] Dennis M.L., Newman J., Dolezal O., Hattarki M., Surjadi R.N., Nuttall S.D. (2020). Crystal structures of human ENPP1 in Apo and bound forms. Acta. Crystallogr. D..

[25] Cornish-Bowden A. (2012).

[26] Kato K., Nishimasu H., Okudaira S., Mihara E., Ishitani R., Takagi J. (2012). Crystal structure of Enpp1, an extracellular glycoprotein involved in bone mineralization and insulin signaling. Proc. Natl. Acad. Sci. U. S. A..

[27] Crecelius A.R., Kirby B.S., Dinenno F.A. (2015). Intravascular ATP and the regulation of blood flow and oxygen delivery in humans. Exerc. Sport. Sci. Rev..

[28] Ledderose C., Valsami E.A., Junger W.G. (2022). Optimized HPLC method to elucidate the complex purinergic signaling dynamics that regulate ATP, ADP, AMP, and adenosine levels in human blood. Purinerg. Signal..

[29] De La Cruz E.M., Pollard T.D. (1995). Nucleotide-free actin - Stabilization by sucrose and nucleotide-binding kinetics. Biochemistry.

[30] Conibear P.B. (1999). Kinetic studies on the effects of ADP and ionic strength on the interaction between myosin subfragment-1 and actin: implications for load-sensitivity and regulation of the crossbridge cycle. J. Muscle. Res. Cell. Motil..

[31] Nitschke Y., Yan Y., Buers I., Kintziger K., Askew K., Rutsch F. (2018). ENPP1-Fc prevents neointima formation in generalized arterial calcification of infancy through the generation of AMP. Exp. Mol. Med..

[32] Zimmerman K., Liu X., von Kroge S., Stabach P., Lester E.R., Chu E.Y. (2022). Catalysis-independent ENPP1 protein signaling regulates mammalian bone mass. J. Bone. Miner. Res..

[33] Jansen R.S., Duijst S., Mahakena S., Sommer D., Szeri F., Váradi A. (2014). ABCC6-Mediated ATP secretion by the liver is the main source of the mineralization inhibitor inorganic pyrophosphate in the systemic circulation-brief report. Arterioscl. Throm. Vas..

[34] Li Q.L., Brodsky J.L., Conlin L.K., Pawel B., Glatz A.C., Gafni R.I. (2014). Mutations in the gene as a cause of generalized arterial calcification of infancy: genotypic overlap with Pseudoxanthoma elasticum. J. Invest. Dermatol..

[35] Ichikawa N., Taniguchi A., Kaneko H., Kawamoto M., Sekita C., Nakajima A. (2015). Arterial calcification due to deficiency of CD73 (ACDC) as one of rheumatic diseases associated with periarticular calcification. J Clin. Rheumatol..

[36] Stabach P.R., Zimmerman K., Adame A., Kavanagh D., Saeui C.T., Agatemor C. (2021). Improving the pharmacodynamics and in vivo activity of ENPP1-Fc through protein and glycosylation engineering. Clin. Transl. Sci..

