# Supplementary material for: Kinetic mechanism of ENPP1 ATPase: Implications for aberrant calcification disorders and enzyme replacement therapy
Source: J Biol Chem. 2025 Aug 5;301(9):110558. doi: 10.1016/j.jbc.2025.110558 (PMC12446537; doi:10.1016/j.jbc.2025.110558)
Supplement: Supplementary Material [file mmc1.docx]

**Supplementary Information**

*Kinetic mechanism of ENPP1 ATPase: Implications for aberrant calcification disorders and enzyme replacement therapy.*

Marisa M. Michalchik^1^, Tony Potchernikov^1^, Ethan R. Lester^2^, Demetrios T. Braddock^2^, Wenxiang Cao^1^, and Enrique M. De La Cruz^1*^

^1^Department of Molecular Biophysics and Biochemistry, Yale University, New Haven, Connecticut, 06520, USA

^2^Department of Pathology, Yale University School of Medicine, New Haven, Connecticut, 06510, USA

*To whom correspondence should be addressed: enrique.delacruz@yale.edu

**Supplementary Figures**

**Supplementary Figure S1. Steady state mant-ATP hydrolysis by ENPP1. A)** Time courses of mant-AMP liberation after mixing ENPP1 (10 nM final) with a range of mant-ATP (3.3-165 μM final). The lines through the data points represent the best fit to Eq. 11 in the main text. **B)** [mant-ATP]-dependence of the initial ENPP1 catalytic turnover rate (*v*_0_). The solid line through the data represents the best fit to a linear function with zero slope, yielding an approximated *k*_cat,mT_ value of 3.0 ± 0.3 sec^−1^. The value of *K*_M,mT_ is too tight to measure using this assay, but is estimated as <1 μM given the weak concentration dependence over the range examined.

**Supplementary Figure S2. ENPP1 does not significantly catalyze the hydrolysis of α,β-methylene ATP.** HPLC chromatograms of α,β-methylene ATP and AMP elution detected by absorbance at 259 nm. ENPP1 (20 nM) was mixed with 10 µM α,β-methylene ATP and equilibrated at 25 °C for 1 minute (solid black line), 10 minutes (thin dashed black line), or overnight (dotted black line). No α,β-methylene ATP degradation or α,β-methylene AMP generation is observed. Elution of 10 µM AMP product alone (solid magenta line) is shown for comparison.

**Supplementary Figure S3. mant-nucleotide binding to ENPP1. A)** Representative time courses of mant-ATP fluorescence increase after mixing ENPP1 (0.1 μM) with mant-ATP (1-10 μM). Time courses shown are the average of at least three individual time courses. Solid lines through the data are best fits to a double exponential function. **B)** [mant-ATP]-dependence of *λ*_obs,mT,fast_ and *λ*_obs,mT,slow_ from fits in panel A. *λ*_obs,mT,fast_ depends linearly on [mant-ATP], yielding a slope ($\frac{k_{+mT}}{K_{mT,c}}$) of 58 ± 2 μM^−1^ sec^−1^ and an intercept (*k*_+AMP′_ + *k*_−AMP_) of 7 ± 3 μM sec^−1^. *λ*_obs,mT,slow_ is a constant with a value of 7 ± 0.5 sec^−1^. **C)** Representative time courses of mant-AMP fluorescence increase after mixing ENPP1 (0.15 μM) with mant-AMP (1-7 μM). Solid lines through the data are best fits to a double exponential function. **D)** [mant-AMP]-dependence of *λ*_obs,mAMP,fast_ and *λ*_obs,mAMP,slow_ from fits in panel C. *λ*_obs,mAMP,fast_ varies hyperbolically with [mant-AMP], yielding a maximum *λ*_obs,mAMP,fast_ ($k_{+mAMP}+k_{+mAMP'}+k_{-mAMP}$) of 595 ± 253 sec^−1^, a midpoint (*K*_mAMP,c_) of 5 ± 5 μM, and an intercept indistinguishable from the origin. Fitting the first three data points to a linear function yields a slope ($\frac{k_{+mAMP}}{K_{mAMP,c}}$) of 78 ± 8 sec^−1^ and an intercept indistinguishable from the origin. *λ*_obs,mAMP,slow_ is a constant with a value of 9 ± 0.2 sec^−1^.

**Supplementary Figure S4. Inhibition of ENPP1 steady state ATPase activity by AMP & PP*_i_*. A)** Time courses of AMP liberation after mixing ENPP1 (20 nM) with a solution of ATP (0.1 µM; with trace [α-^32^P]-ATP) alone (filled black dots) or with added AMP product (0.1 µM; open black dots). Solid lines through the data represent the best fit to Eq. 11, yielding *v*_0_ of 0.95 ± 0.09 and 0.55 ± 0.01 µM, respectively. **B)** Time courses of AMP liberation after mixing ENPP1 (20 nM) with a solution of ATP (10 µM; with trace [α-^32^P]-ATP) alone (filled black dots) or with added PP*_i_* product (100 µM; open black dots). Solid lines through the data represent the best fit to Eq. 12, yielding *v*_0_ of 1.4 ± 0.05 and 1.5 ± 0.2 µM, respectively.

**Derivations for kinetic analysis of the ENPP1 ATPase reaction scheme**

The minimum catalytic ENPP1 ATPase cycle determined in this study is defined by eight reversible biochemical transitions (Scheme 1):


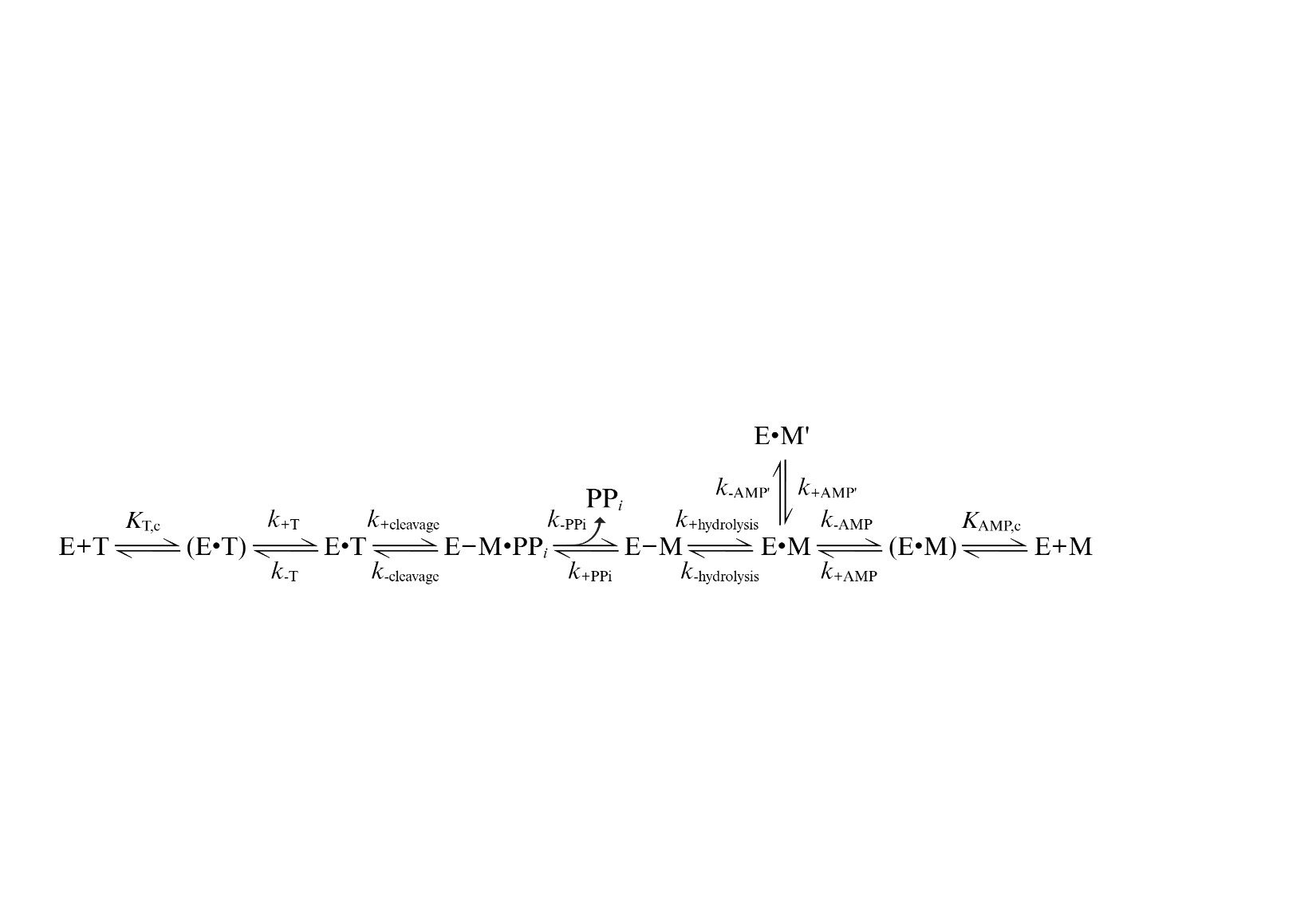
 Scheme 1

ENPP1 (E) and ATP (T) rapidly equilibrate (*K*_T,c_) to form a transient collision complex ((E∙T)), which isomerizes (*K*_T_) to the ENPP1-bound ATP state (E∙T). The initial cleavage of ATP (*K*_cleavage_) involves the formation of a nucleotidylated ENPP1-AMP intermediate with non-covalently bound PP*_i_* (E−M∙PP*_i_*). The PP*_i_* product is released (*K*_PP_*_i_*), and the nucleotidylated intermediate (E−M) is hydrolyzed to yield ENPP1 with AMP non-covalently bound (E∙M). Accordingly, PP*_i_* release and nucleotidylated intermediate hydrolysis are modeled as sequential, ordered steps, but they could occur simultaneously or in a different sequence with no change in the resulting derivation.

All biochemical transitions between the E∙T and E∙M states are rapid (complete within ~15 milliseconds; λ_obs_ >>250 sec^−1^, Fig. 6) and essentially irreversible, such that initial ATP cleavage (*K*_cleavage_), PP*_i_* release (*K*_PP_*_i_*) and covalent intermediate hydrolysis (*K*_hydrolysis_) can be modeled as a single transition (*K*_internal_, where the parentheses indicate rapid equilibria) and Scheme 1 can be reduced to Scheme 2:

$E\cdot M'$

$k_{-AMP'}⇃↾k_{+AMP'}$

$$\begin{aligned} E+T\underset{\rightleftharpoons}{K_{T,c}}\left( E\cdot T \right)\underset{k_{-T}}{\underset{\rightleftharpoons}{k_{+T}}}E\cdot T\left( \underset{k_{-internal}}{\underset{\rightleftharpoons}{k_{+internal}}} \right)E\cdot M\underset{k_{+AMP}}{\underset{\rightleftharpoons}{k_{-AMP}}}\left( E\cdot M \right)\underset{\rightleftharpoons}{K_{AMP,c}}E+M\#Scheme 2 \end{aligned}$$

The E∙M complex either isomerizes (*K*_M′_) to an off-pathway AMP-bound state (E∙M′), or AMP is reversibly released (*K*_AMP_*K*_AMP,c_) to regenerate free ENPP1 (E).

The following system of differential equations describes the transient population changes of all substrate- and product-bound biochemical intermediates as defined by Scheme 2:

$$\begin{aligned} \frac{d\left[ E\cdot T \right]}{dt}=k_{+T}\left[ (E\cdot T) \right]-\left( k_{-T}+k_{+internal} \right)\left[ E\cdot T \right]+k_{-internal}\left[ E\cdot M \right]\#S1 \end{aligned}$$

$$\begin{aligned} \frac{d\left[ E\cdot M \right]}{dt}=k_{+internal}\left[ E\cdot T \right]-\left( k_{-internal}+k_{+{AMP}^{'}}+k_{-AMP} \right)\left[ E\cdot M \right]+k_{-{AMP}^{'}}\left[ E{\cdot M}^{'} \right]+k_{+AMP}\left[ (E\cdot M) \right]\#S2 \end{aligned}$$

$$\begin{aligned} \frac{d\left[ E{\cdot M}^{'} \right]}{dt}=k_{+{AMP}^{'}}\left[ E\cdot M \right]-k_{-A{MP}^{'}}\left[ E\cdot M^{'} \right]\#S3 \end{aligned}$$

The following (dissociation) equilibrium constants represent the rapid equilibrium formation of collision complexes during ATP and AMP binding, respectively.

$$\begin{aligned} K_{T,c}=\frac{\left[ E \right]\left[ T \right]}{\left[ (E\cdot T) \right]}\#S4 \end{aligned}$$

$$\begin{aligned} K_{AMP,c}=\frac{\left[ E \right]\left[ M \right]}{\left[ (E\cdot M) \right]}\#S5 \end{aligned}$$

The following equation describes all possible biochemical states defined in Scheme 2 and constrained by mass conservation, where E_0_ is the total initial [ENPP1].

$$\begin{aligned} E_{0}=\left[ E \right]+\left[ (E\cdot T) \right]+\left[ E\cdot T \right]+\left[ E\cdot M \right]+\left[ E{\cdot M}^{'} \right]+\left[ (E\cdot M) \right]\#S6 \end{aligned}$$

1. ***ATP binding*** ***(Fig. 3)***

Since the observed fast and slow phases of ATP binding are well separated in time (i.e. λ_obs,fast_ >> λ_obs,slow_), we can treat the two phases independently.

***Fast phase – early time region***

In the initial time region, E∙M’ and (E∙M) are not yet significantly populated, such that [E∙M’]≈[(E∙M)]≈[M]≈0. Combining Eq. S1 and Eq. S2 and incorporating this assumption yields the following equation:

$$\frac{d\left( \left[ E\cdot T \right]+\left[ E\cdot M \right] \right)}{dt}=k_{+T}\left[ (E\cdot T) \right]-k_{-T}\left[ E\cdot T \right]-\left( k_{+A{MP}^{'}}+k_{-AMP} \right)\left[ E\cdot M \right]+k_{-{AMP}^{'}}\left[ E\cdot M^{'} \right]+k_{+AMP}\left[ (E\cdot M) \right]$$

$$\begin{aligned} \sim k_{+T}\left[ (E\cdot T) \right]-k_{-T}\left[ E\cdot T \right]-\left( k_{+A{MP}^{'}}+k_{-AMP} \right)\left[ E\cdot M \right]\#S7 \end{aligned}$$

Experimental data demonstrates that interconversion from [E∙T] to [E∙M] (*K*_internal_) is complete in ~20 msec (Fig. 6), such that this step can be treated as instantaneous and irreversible ([E∙T] ~0), and the following approximation can be made in Eq. S7:

$$\begin{aligned} \frac{d\left[ E\cdot M \right]}{dt}=k_{+T}\left[ (E\cdot T) \right]-\left( k_{+A{MP}^{'}}+k_{-AMP} \right)\left[ E\cdot M \right]\#S8 \end{aligned}$$

Using the above assumptions for the earlier time region, the mass conservation equation (Eq. S6) becomes:

$$E_{0}=\left[ E \right]+\left[ (E\cdot T) \right]+\left[ E\cdot T \right]+\left[ E\cdot M \right]+\left[ E{\cdot M}^{'} \right]+\left[ (E\cdot M) \right]$$

$$\sim\left[ E \right]+\left[ (E\cdot T) \right]+\left[ E\cdot M \right]=\frac{K_{T,c}\left[ (E\cdot T) \right]}{\left[ T \right]}+\left[ (E\cdot T) \right]+\left[ E\cdot M \right]=\frac{K_{T,c}+\left[ T \right]}{\left[ T \right]}\left[ (E\cdot T) \right]+\left[ E\cdot M \right]$$

i.e.,

$$\begin{aligned} \left[ (E\cdot T) \right]=\frac{E_{0}-\left[ E\cdot M \right]}{K_{T,c}+\left[ T \right]}\left[ T \right]\#S9 \end{aligned}$$

Assuming pseudo first-order reaction conditions ([T] >>[E]), substituting Eq. S9 in Eq. S8 yields:

$$\frac{d\left[ E\cdot M \right]}{dt}=k_{+T}\left[ (E\cdot T) \right]-\left( k_{+{AMP}^{'}}+k_{-AMP} \right)\left[ E\cdot M \right]=k_{+T}\frac{E_{0}-\left[ E\cdot M \right]}{K_{T,c}+\left[ T \right]}\left[ T \right]-\left( k_{+A{MP}^{'}}+k_{-AMP} \right)\left[ E\cdot M \right]$$

$$\begin{aligned} =\frac{{k_{+T}E}_{0}\left[ T \right]}{K_{T,c}+\left[ T \right]}-\left( \frac{k_{+T}\left[ T \right]}{K_{T,c}+\left[ T \right]}+k_{+A{MP}^{'}}+k_{-AMP} \right)\left[ E\cdot M \right]\#S10 \end{aligned}$$

Solving Eq. 10 yields the **fast phase** observed rate constant (*λ*_obs,T,fast_) for binding of ATP to ENPP1:

$$\begin{aligned} \lambda_{obs, T,fast}=\frac{k_{+T}\left[ T \right]}{K_{T,c}+\left[ T \right]}+k_{+A{MP}^{'}}+k_{-AMP}\#S11 \end{aligned}$$

*λ*_obs,T,fast_ depends hyperbolically on [T], with a y-intercept of $k_{+A{MP}^{'}}+k_{-AMP}$, a midpoint of *K*_T,c_ and a maximum value of $k_{+T}+k_{+{AMP}^{'}}+k_{-AMP}$ at saturating [T].

When [T] <<*K*_T,c_ and pseudo first-order conditions are fulfilled, [T] in the denominator can be ignored, as the [T]-dependence in this concentration range appears linear according to the below equation:

$$\begin{aligned} \lambda_{obs, T,f}=\frac{k_{+T}}{K_{T,c}}\left[ T \right]+k_{+{AMP}^{'}}+k_{-AMP}\#S11' \end{aligned}$$

The intercept is identical to the hyperbolic form, and the slope yields the second order association rate constant for ATP binding ($k_{+T}$/$K_{T,c}$).

***Slow phase – “late” time region***

In the later time region when the initial fast ATP binding phase is complete, the AMP bound species E∙M equilibrates and reaches a steady state ( $\frac{d\left( \left[ E\cdot M \right] \right)}{dt}=0$), and the off-pathway product-bound species E∙M′ begins to form. Accumulation of AMP product on short time scales (≤5 sec for all binding experiments) is insignificant in terms of product inhibition and can be ignored ([(E∙M)]≈[M]≈0).

Under these conditions, Eq. S7 becomes:

$$\frac{d\left( \left[ E\cdot M \right] \right)}{dt}\sim k_{+T}\left[ (E\cdot T) \right]-\left( k_{+A{MP}^{'}}+k_{-AMP} \right)\left[ E\cdot M \right]+k_{-{AMP}^{'}}\left[ E\cdot M^{'} \right]=0$$

After rearranging the terms, we have:

$$\begin{aligned} \left[ (E\cdot T) \right]=\frac{\left( k_{+{AMP}^{'}}+k_{-AMP} \right)\left[ E\cdot M \right]-k_{-A{MP}^{'}}\left[ E{\cdot M}^{'} \right]}{k_{+T}}\#S12 \end{aligned}$$

The mass conservation equation (Eq. S6) becomes:

$$E_{0}=\left[ E \right]+\left[ (E\cdot T) \right]+\left[ E\cdot T \right]+\left[ E\cdot M \right]+\left[ E\cdot M^{'} \right]+\left[ (E\cdot M) \right]\sim\left[ E \right]+\left[ (E\cdot T) \right]+\left[ E\cdot M \right]+\left[ E\cdot M^{'} \right]$$

$$=\frac{K_{T,c}+\left[ T \right]}{\left[ T \right]}\left[ (E\cdot T) \right]+\left[ E\cdot M \right]+\left[ E\cdot M^{'} \right]$$

$$=\frac{K_{T,c}+\left[ T \right]}{\left[ T \right]}\frac{\left( k_{+{AMP}^{'}}+k_{-AMP} \right)\left[ E\cdot M \right]-k_{-{AMP}^{'}}\left[ E{\cdot M}^{'} \right]}{k_{+T}}+\left[ E\cdot M \right]+\left[ E{\cdot M}^{'} \right]$$

$$=\frac{\left( \left( K_{T,c}+\left[ T \right] \right)\left( k_{+A{MP}^{'}}+k_{-AMP} \right)+k_{+T}\left[ T \right] \right)\left[ E\cdot M \right]+\left( k_{+T}\left[ T \right]-{\left( K_{T,c}+\left[ T \right] \right)k}_{-{AMP}^{'}} \right)\left[ E\cdot M^{'} \right]}{k_{+T}\left[ T \right]}$$

$$=\frac{\left( K_{T,c}\left( k_{+{AMP}^{'}}+k_{-AMP} \right)+\left( k_{+T}+k_{+{AMP}^{'}}+k_{-AMP} \right)\left[ T \right] \right)\left[ E\cdot M \right]+\left( \left( {k_{+T}-k}_{-A{MP}^{'}} \right)\left[ T \right]-K_{T,c}k_{-M^{'}} \right)\left[ E{\cdot M}^{'} \right]}{k_{+T}\left[ T \right]}$$

Rearranging terms yields:

$$\left[ E\cdot M \right]=\frac{k_{+T}\left[ T \right]E_{0}}{K_{T,c}\left( k_{+{AMP}^{'}}+k_{-AMP} \right)+\left( k_{+T}+k_{+A{MP}^{'}}+k_{-AMP} \right)\left[ T \right]}$$

$$\begin{aligned} -\frac{\left( {k_{+T}-k}_{-A{MP}^{'}} \right)\left[ T \right]-K_{T,c}k_{-A{MP}^{'}}}{K_{T,c}\left( k_{+{AMP}^{'}}+k_{-AMP} \right)+\left( k_{+T}+k_{+A{MP}^{'}}+k_{-AMP} \right)\left[ T \right]}\left[ E{\cdot M}^{'} \right]\#S13 \end{aligned}$$

Substituting Eq. S13 into Eq. S3 yields:

$$\frac{d\left[ E\cdot M^{'} \right]}{dt}=\frac{k_{+T}\left[ T \right]k_{+{AMP}^{'}}E_{0}}{K_{T,c}\left( k_{+{AMP}^{'}}+k_{-AMP} \right)+\left( k_{+T}+k_{+A{MP}^{'}}+k_{-AMP} \right)\left[ T \right]}$$

$$\begin{aligned} -\left( k_{+{AMP}^{'}}\frac{\left( {k_{+T}-k}_{-A{MP}^{'}} \right)\left[ T \right]-K_{T,c}k_{-A{MP}^{'}}}{K_{T,c}\left( k_{+A{MP}^{'}}+k_{-AMP} \right)+\left( k_{+T}+k_{+A{MP}^{'}}+k_{-AMP} \right)\left[ T \right]}+k_{-{AMP}^{'}} \right)\left[ E\cdot M^{'} \right]\#S14 \end{aligned}$$

The observed **slow phase** in ATP binding is associated with populating the [E∙M’] state and has an observed rate constant (*λ*_obs,T,slow_) with the following identity:

$$\lambda_{obs, T,slow}=k_{+{AMP}^{'}}\frac{\left( {k_{+T}-k}_{-{AMP}^{'}} \right)\left[ T \right]-K_{T,c}k_{-A{MP}^{'}}}{K_{T,c}\left( k_{+A{MP}^{'}}+k_{-AMP} \right)+\left( k_{+T}+k_{+{AMP}^{'}}+k_{-AMP} \right)\left[ T \right]}+k_{-{AMP}^{'}}$$

$$=k_{+A{MP}^{'}}\frac{\frac{-k_{-{AMP}^{'}}}{k_{+{AMP}^{'}}+k_{-AMP}}\left( K_{T,c}\left( k_{+A{MP}^{'}}+k_{-AMP} \right)+\left( k_{+T}+k_{+A{MP}^{'}}+k_{-AMP} \right)\left[ T \right]-\left( k_{+T}+k_{+{AMP}^{'}}+k_{-AMP} \right)\left[ T \right] \right)}{K_{T,c}\left( k_{+A{MP}^{'}}+k_{-AMP} \right)+\left( k_{+T}+k_{+{AMP}^{'}}+k_{-AMP} \right)\left[ T \right]}$$

$$+\frac{k_{+{AMP}^{'}}\left( {k_{+T}-k}_{-A{MP}^{'}} \right)\left[ T \right]}{K_{T,c}\left( k_{+{AMP}^{'}}+k_{-AMP} \right)+\left( k_{+T}+k_{+A{MP}^{'}}+k_{-AMP} \right)\left[ T \right]}+k_{-{AMP}^{'}}$$

$$=k_{-A{MP}^{'}}-\frac{k_{+A{MP}^{'}}k_{-A{MP}^{'}}}{k_{+A{MP}^{'}}+k_{-AMP}}+k_{+A{MP}^{'}}\frac{\left( {k_{+T}-k}_{-A{MP}^{'}} \right)+\frac{k_{-{AMP}^{'}}\left( k_{+T}+k_{+A{MP}^{'}}+k_{-AMP} \right)}{k_{+M^{'}}+k_{-M}}}{K_{T,c}\left( k_{+{AMP}^{'}}+k_{-AMP} \right)+\left( k_{+T}+k_{+A{MP}^{'}}+k_{-AMP} \right)\left[ T \right]}\left[ T \right]$$

$$=\frac{k_{-AMP}k_{-A{MP}^{'}}}{k_{+A{MP}^{'}}+k_{-AMP}}+k_{+A{MP}^{'}}\frac{\frac{k_{+T}\left( k_{+A{MP}^{'}}+k_{-A{MP}^{'}}+k_{-AMP} \right)}{k_{+{AMP}^{'}}+k_{-AMP}}}{K_{T,c}\left( k_{+A{MP}^{'}}+k_{-AMP} \right)+\left( k_{+T}+k_{+A{MP}^{'}}+k_{-AMP} \right)\left[ T \right]}\left[ T \right]$$

$$=\frac{k_{-AMP}k_{-A{MP}^{'}}}{k_{+A{MP}^{'}}+k_{-AMP}}+\frac{\frac{k_{+A{MP}^{'}}k_{+T}\left( k_{+{AMP}^{'}}+k_{-{AMP}^{'}}+k_{-AMP} \right)}{\left( k_{+A{MP}^{'}}+k_{-AMP} \right)\left( k_{+T}+k_{+A{MP}^{'}}+k_{-AMP} \right)}\left[ T \right]}{\frac{K_{T,c}\left( k_{+A{MP}^{'}}+k_{-AMP} \right)}{k_{+T}+k_{+{AMP}^{'}}+k_{-AMP}}+\left[ T \right]}$$

$$\begin{aligned} =\frac{k_{-A{MP}^{'}}}{1+\frac{k_{+{AMP}^{'}}}{k_{-AMP}}}+\frac{\frac{k_{+{AMP}^{'}}+k_{-A{MP}^{'}}+k_{-AMP}}{\left( 1+k_{-AMP}/k_{+{AMP}^{'}} \right)\left( 1+\left( k_{+A{MP}^{'}}+k_{-AMP} \right)/k_{+T} \right)}\left[ T \right]}{\frac{K_{T,c}}{\frac{k_{+T}}{k_{+A{MP}^{'}}+k_{-AMP}}+1}+\left[ T \right]}\#S15 \end{aligned}$$

*λ*_obs,T,slow_ depends hyperbolically on [T], with an intercept of $\frac{k_{-{AMP}^{'}}}{1+k_{+{AMP}^{'}}/k_{-AMP}}<k_{-A{MP}^{'}}$, a hyperbolic midpoint of $\frac{K_{T,c}}{\frac{k_{+T}}{k_{+AMP'}+k_{-AMP}}+1}<K_{T,c}$, and a maximum of $\frac{k_{-{AMP}^{'}}}{1+\frac{k_{+{AMP}^{'}}}{k_{-AMP}}}+\frac{k_{+A{MP}^{'}}+k_{-A{MP}^{'}}+k_{-AMP}}{\left( 1+k_{-AMP}/k_{+{AMP}^{'}} \right)\left( 1+\left( k_{+A{MP}^{'}}+k_{-AMP} \right)/k_{+T} \right)}<k_{+A{MP}^{'}}+2k_{-A{MP}^{'}}+k_{-AMP}$.

***Case 1: λ_obs,T,slow_ displays a linear [T]-dependence when*** $\frac{K_{T,c}}{\frac{k_{+T}}{k_{+A{MP}^{'}}+k_{-AMP}}+1}>[T]$

When ${k_{+T}<k}_{+A{MP}^{'}}+k_{-AMP}$, the midpoint ($\frac{K_{T,c}}{\frac{k_{+T}}{k_{+{AMP}^{'}}+k_{-AMP}}+1}$) is approximately equivalent to *K*_T,c_. With this approximation, *λ*_obs,T,slow_ is linear when *K*_T,c_ >>[ATP] used, i.e.:

$$\lambda_{obs, T,slow}\sim\frac{k_{-{AMP}^{'}}}{1+\frac{k_{+A{MP}^{'}}}{k_{-AMP}}}+\frac{\frac{k_{+{AMP}^{'}}k_{+T}\left( k_{+A{MP}^{'}}+k_{-A{MP}^{'}}+k_{-AMP} \right)}{\left( k_{+{AMP}^{'}}+k_{-AMP} \right)\left( k_{+T}+k_{+A{MP}^{'}}+k_{-AMP} \right)}\left[ T \right]}{\frac{K_{T,c}\left( k_{+A{MP}^{'}}+k_{-AMP} \right)}{k_{+T}+k_{+A{MP}^{'}}+k_{-AMP}}}$$

$$\begin{aligned} =\frac{k_{-{AMP}^{'}}}{1+\frac{k_{+A{MP}^{'}}}{k_{-AMP}}}+\frac{\left( 1+\frac{k_{-{AMP}^{'}}}{k_{+A{MP}^{'}}+k_{-AMP}} \right)}{\left( 1+\frac{k_{-AMP}}{k_{+A{MP}^{'}}} \right)}\frac{k_{+T}}{K_{T,c}}\left[ T \right]\#S \end{aligned}$$

***Case 2: λ_obs,T,slow_ is constant when*** $\frac{K_{T,c}}{\frac{k_{+T}}{k_{+A{MP}^{'}}+k_{-AMP}}+1}<<[T]$ ***(Fig .4)***

When ${k_{+T}\gg k}_{+A{MP}^{'}}+k_{-AMP}$ and the [ATP] used is much larger than the midpoint, ($\frac{K_{T,c}}{\frac{k_{+T}}{k_{+A{MP}^{'}}+k_{-AMP}}+1}<<[T]$), *λ*_obs,T,slow_ reaches a maximum and remains constant, i.e.;

$$\lambda_{obs, T,slow}\sim\frac{k_{-{AMP}^{'}}}{1+\frac{k_{+{AMP}^{'}}}{k_{-AMP}}}+\frac{\frac{k_{+A{MP}^{'}}+k_{-A{MP}^{'}}+k_{-AMP}}{\left( 1+k_{-AMP}/k_{+A{MP}^{'}} \right)\left( 1+\left( k_{+A{MP}^{'}}+k_{-AMP} \right)/k_{+T} \right)}\left[ T \right]}{[T]}=$$

$$\begin{aligned} =\frac{k_{-A{MP}^{'}}}{1+\frac{k_{+A{MP}^{'}}}{k_{-AMP}}}+\frac{k_{+{AMP}^{'}}+k_{-{AMP}^{'}}+k_{-AMP}}{\left( 1+\frac{k_{-AMP}}{k_{+{AMP}^{'}}} \right)\left( 1+\frac{k_{+A{MP}^{'}}+k_{-AMP}}{k_{+T}} \right)}<k_{+A{MP}^{'}}+2k_{-A{MP}^{'}}+k_{-AMP}\#S17 \end{aligned}$$

1. ***AMP binding (Fig. 4)***

Since the intermediate biochemical transitions from E∙T to E∙M in Scheme 1 are nearly irreversible, it is assumed that the nucleotidylated ENPP1 intermediate (E−AMP) does not reform during AMP binding experiments, so that only the E, E∙M, and E∙M′ states are populated. As seen in ATP binding, the observed fast and slow AMP binding phases (Fig. 4B) are well separated in time (i.e. λ_obs,fast_ >> λ_obs,slow_) and can be treated independently.

***Fast phase – early time region***

In the earlier time region when the E∙M′ state is not significantly populated ([E∙M′]≈0), Eq. S2 simplifies to

$$\begin{aligned} \frac{d\left[ E\cdot M \right]}{dt}\sim-\left( k_{+A{MP}^{'}}+k_{-AMP} \right)\left[ E\cdot M \right]+k_{+AMP}\left[ (E\cdot M) \right]\#S18 \end{aligned}$$

Using the mass conservation (Eq. S6) and equilibrium equations (Eq. S5), incorporating this assumption yields:

$$E_{0}\sim\left[ E\cdot M \right]+\left[ (E\cdot M) \right]+\left[ E \right]=\left[ E\cdot M \right]+\frac{K_{AMP,c}+[M]}{[M]}\left[ (E\cdot M) \right]$$

and

$$\begin{aligned} \left[ (E\cdot M) \right]=\frac{E_{0}-\left[ E\cdot M \right]}{K_{AMP,c}+[M]}\left[ M \right]\#S19 \end{aligned}$$

Substituting Eq. S19 into Eq. S18 yields:

$$\begin{aligned} \frac{d\left[ E\cdot M \right]}{dt}\sim\frac{k_{+AMP}\left[ M \right]E_{0}}{K_{AMP,c}+[M]}-\left( k_{+A{MP}^{'}}+k_{-AMP}+\frac{k_{+AMP}\left[ M \right]}{K_{{AMP,}_{c}}+[M]} \right)\left[ E\cdot M \right]\#S20 \end{aligned}$$

The **fast phase** observed rate constant for AMP binding to ENPP1 (*k*_obs,AMP,fast_) is a hyperbolic function of [*M*], i.e.:

$$\begin{aligned} \lambda_{obs, AMP,fast}=k_{+A{MP}^{'}}+k_{-AMP}+\frac{k_{+AMP}\left[ M \right]}{K_{{AMP,}_{c}}+\left[ M \right]}\#S21 \end{aligned}$$

with an intercept of $k_{+{AMP}^{'}}+k_{-AMP}$, a midpoint of *K*_AMP,c_, and a maximum value of $k_{+AMP}+k_{+A{MP}^{'}}+k_{-AMP}$ at saturating [*M*]. Eq. S21 is very similar to that for ATP binding (Eq. S11), with [*T*] ↔ [*M*], *k*_+T_ ↔ *k*_+AMP_, and an identical intercept.

As seen for ATP binding, if [*M*] <<*K*_AMP,c_ the AMP concentration in the denominator can be ignored, and thus the hyperbolic equation (Eq. S21) becomes linear:

$$\begin{aligned} \lambda_{obs,AMP,fast}=\frac{k_{+AMP}}{K_{AMP,c}}\left[ M \right]+k_{+{AMP}^{'}}+k_{-AMP}\#S21' \end{aligned}$$

The intercept is identical to the hyperbolic form, and the slope yields the second order association rate constant $k_{+AMP}$/$K_{AMP,c}$.

***Slow phase – “late” time region***

In the later time region when the initial fast AMP binding phase is complete, the AMP bound species E∙M reaches a steady state ( $\frac{d\left( \left[ E\cdot M \right] \right)}{dt}=0$), and off-pathway product-bound species E∙M′ begins to form, and Eq. S2 becomes:

$$\frac{d\left[ E\cdot M \right]}{dt}\sim-\left( k_{+{AMP}^{'}}+k_{-AMP} \right)\left[ E\cdot M \right]+k_{-A{MP}^{'}}\left[ E\cdot M^{'} \right]+k_{+AMP}\left[ (E\cdot M) \right]=0$$

i.e.

$$\begin{aligned} \left[ (E\cdot M) \right]=\frac{\left( k_{+A{MP}^{'}}+k_{-AMP} \right)\left[ E\cdot M \right]-k_{-A{MP}^{'}}\left[ E\cdot M^{'} \right]}{k_{+AMP}}\#S22 \end{aligned}$$

Combining mass conservation (Eq. S6), the equilibrium binding constant equation for the ENPP1∙AMP collision complex (Eq. S5), and Eq. S22 yields:

$$E_{0}=\left[ E\cdot M \right]+\left[ E\cdot M^{'} \right]+\left[ (E\cdot M) \right]+\left[ E \right]=\left[ E\cdot M \right]+\left[ E\cdot M^{'} \right]+\frac{K_{{AMP,}_{c}}+[M]}{[M]}\left[ (E\cdot M) \right]$$

$$=\left[ E\cdot M \right]+\left[ E\cdot M^{'} \right]+\frac{K_{{AMP,}_{c}}+[M]}{[M]}\frac{\left( k_{+A{MP}^{'}}+k_{-AMP} \right)\left[ E\cdot M \right]-k_{-A{MP}^{'}}\left[ E\cdot M^{'} \right]}{k_{+AMP}}$$

$$=\frac{k_{+AMP}\left[ M \right]+\left( K_{{AMP,}_{c}}+[M] \right)\left( k_{+{AMP}^{'}}+k_{-AMP} \right)}{k_{+AMP}[M]}\left[ E\cdot M \right]+\frac{k_{+AMP}[M]-\left( K_{A{MP,}_{c}}+[M] \right)k_{-A{MP}^{'}}}{k_{+AMP}[M]}\left[ E\cdot M^{'} \right]$$

$$=\frac{K_{{AMP,}_{c}}\left( k_{+A{MP}^{'}}+k_{-AMP} \right)+\left( k_{+AMP}+k_{+A{MP}^{'}}+k_{-AMP} \right)[M]}{k_{+AMP}[M]}\left[ E\cdot M \right]+\frac{-K_{{AMP,}_{c}}k_{-A{MP}^{'}}+\left( k_{+AMP}-k_{-{AMP}^{'}} \right)[M]}{k_{+AMP}[M]}\left[ E{\cdot M}^{'} \right]$$

i.e.,

$$\left[ E\cdot M \right]=\frac{k_{+AMP}\left[ M \right]E_{0}}{K_{{AMP,}_{c}}\left( k_{+{AMP}^{'}}+k_{-AMP} \right)+\left( k_{+AMP}+k_{+{AMP}^{'}}+k_{-AMP} \right)\left[ M \right]}$$

$$\begin{aligned} -\frac{-K_{{AMP,}_{c}}k_{-{AMP}^{'}}+\left( k_{+AMP}-k_{-{AMP}^{'}} \right)\left[ M \right]}{K_{A{MP,}_{c}}\left( k_{+{AMP}^{'}}+k_{-AMP} \right)+\left( k_{+AMP}+k_{+{AMP}^{'}}+k_{-AMP} \right)\left[ M \right]}\left[ E\cdot M^{'} \right]\#S23 \end{aligned}$$

Substituting this equation into Eq. S3 yields:

$$\frac{d\left[ E\cdot M^{'} \right]}{dt}=\frac{k_{+AMP}\left[ M \right]k_{+{AMP}^{'}}E_{0}}{K_{{AMP,}_{c}}\left( k_{+{AMP}^{'}}+k_{-AMP} \right)+\left( k_{+AMP}+k_{+A{MP}^{'}}+k_{-AMP} \right)\left[ M \right]}$$

$$\begin{aligned} -\left( k_{-A{MP}^{'}}+k_{+A{MP}^{'}}\frac{-K_{{AMP,}_{c}}k_{-A{MP}^{'}}+\left( k_{+AMP}-k_{-{AMP}^{'}} \right)\left[ M \right]}{K_{{AMP,}_{c}}\left( k_{+A{MP}^{'}}+k_{-AMP} \right)+\left( k_{+AMP}+k_{+A{MP}^{'}}+k_{-AMP} \right)\left[ M \right]} \right)\left[ E{\cdot M}^{'} \right]\#S24 \end{aligned}$$

The observed **slow phase** rate constant for populating the E∙M′ state during AMP binding is:

$$\lambda_{obs,AMP,slow}=k_{-{AMP}^{'}}+k_{+{AMP}^{'}}\frac{-K_{{AMP,}_{c}}k_{-{AMP}^{'}}+\left( k_{+AMP}-k_{-{AMP}^{'}} \right)\left[ M \right]}{K_{A{MP,}_{c}}\left( k_{+A{MP}^{'}}+k_{-AMP} \right)+\left( k_{+AMP}+k_{+A{MP}^{'}}+k_{-AMP} \right)\left[ M \right]}$$

$$=k_{-A{MP}^{'}}+k_{+{AMP}^{'}}\frac{\left( k_{+AMP}-k_{-A{MP}^{'}} \right)\left[ M \right]}{K_{A{MP,}_{c}}\left( k_{+{AMP}^{'}}+k_{-AMP} \right)+\left( k_{+AMP}+k_{+A{MP}^{'}}+k_{-AMP} \right)\left[ M \right]}$$

$$+k_{+{AMP}^{'}}\frac{-\frac{k_{-{AMP}^{'}}}{k_{+A{MP}^{'}}+k_{-AMP}}\left( K_{{AMP,}_{c}}\left( k_{+A{MP}^{'}}+k_{-AMP} \right)+\left( k_{+AMP}+k_{+{AMP}^{'}}+k_{-AMP} \right)\left[ M \right]-\left( k_{+AMP}+k_{+A{MP}^{'}}+k_{-AMP} \right)\left[ M \right] \right)}{K_{{AMP,}_{c}}\left( k_{+{AMP}^{'}}+k_{-AMP} \right)+\left( k_{+AMP}+k_{+A{MP}^{'}}+k_{-AMP} \right)\left[ M \right]}$$

$$=k_{-{AMP}^{'}}-\frac{k_{+A{MP}^{'}}k_{-{AMP}^{'}}}{k_{+A{MP}^{'}}+k_{-AMP}}+k_{+A{MP}^{'}}\frac{\left( k_{+AMP}-k_{-A{MP}^{'}} \right)+\frac{k_{-{AMP}^{'}}}{k_{+{AMP}^{'}}+k_{-AMP}}\left( k_{+AMP}+k_{+A{MP}^{'}}+k_{-AMP} \right)}{K_{A{MP,}_{c}}\left( k_{+{AMP}^{'}}+k_{-AMP} \right)+\left( k_{+AMP}+k_{+A{MP}^{'}}+k_{-AMP} \right)\left[ M \right]}\left[ M \right]$$

$$=\frac{k_{-AMP}k_{-A{MP}^{'}}}{k_{+{AMP}^{'}}+k_{-AMP}}+k_{+{AMP}^{'}}\frac{\frac{k_{+AMP}-k_{-A{MP}^{'}}}{k_{+AMP}+k_{+A{MP}^{'}}+k_{-AMP}}+\frac{k_{-{AMP}^{'}}}{k_{+A{MP}^{'}}+k_{-AMP}}}{\frac{K_{{AMP,}_{c}}\left( k_{+A{MP}^{'}}+k_{-AMP} \right)}{k_{+AMP}+k_{+{AMP}^{'}}+k_{-AMP}}+\left[ M \right]}\left[ M \right]$$

$$=\frac{k_{-AMP}k_{-{AMP}^{'}}}{k_{+A{MP}^{'}}+k_{-AMP}}+k_{+AMP}k_{+A{MP}^{'}}\frac{\frac{k_{+A{MP}^{'}}+k_{-{AMP}^{'}}+k_{-AMP}}{\left( k_{+AMP}+k_{+A{MP}^{'}}+k_{-AMP} \right)\left( k_{+A{MP}^{'}}+k_{-AMP} \right)}}{\frac{K_{{AMP,}_{c}}\left( k_{+{AMP}^{'}}+k_{-AMP} \right)}{k_{+AMP}+k_{+{AMP}^{'}}+k_{-AMP}}+\left[ M \right]}\left[ M \right]$$

$$\begin{aligned} =\frac{k_{-{AMP}^{'}}}{1+\frac{k_{+{AMP}^{'}}}{k_{-AMP}}}+\frac{\frac{k_{+{AMP}^{'}}+k_{-A{MP}^{'}}+k_{-AMP}}{\left( 1+\left( k_{+{AMP}^{'}}+k_{-AMP} \right)/k_{+AMP} \right)\left( 1+k_{-AMP}/k_{+A{MP}^{'}} \right)}}{\frac{K_{{AMP,}_{c}}}{1+\frac{k_{+AMP}}{k_{+{AMP}^{'}}+k_{-AMP}}}+\left[ M \right]}\left[ M \right]\#S25 \end{aligned}$$

Eqs. S25 and Eq. S15 (*λ*_obs,ATP,slow_) are symmetric. *λ*_obs,AMP,slow_ depends hyperbolically on the [AMP], with an intercept of $\frac{k_{-{AMP}^{'}}}{1+k_{+{AMP}^{'}}/k_{-AMP}}<k_{-{AMP}^{'}}$, a midpoint of $\frac{K_{{AMP,}_{c}}}{1+k_{+AMP}/\left( k_{+{AMP}^{'}}+k_{-AMP} \right)}<K_{{AMP,}_{c}}$, and a maximum of $\frac{k_{-{AMP}^{'}}}{1+\frac{k_{+{AMP}^{'}}}{k_{-AMP}}}+\frac{k_{+A{MP}^{'}}+k_{-AMP}+k_{-A{MP}^{'}}}{\left( 1+\left( k_{+{AMP}^{'}}+k_{-AMP} \right)/k_{+AMP} \right)\left( 1+k_{-AMP}/k_{+A{MP}^{'}} \right)}<k_{+A{MP}^{'}}+2k_{-A{MP}^{'}}+k_{-AMP}$ at saturating [M].

***Case 1 –*** ${\boldsymbol{k}_{\boldsymbol{+AMP}}\boldsymbol{<k}}_{\boldsymbol{+A}\boldsymbol{MP}^{\boldsymbol{'}}}\boldsymbol{+}\boldsymbol{k}_{\boldsymbol{-AMP}}$ ***– linear [M]-dependence of λ_obs,AMP,slow_***

When ${k_{+AMP}<k}_{+A{MP}^{'}}+k_{-AMP}$, the midpoint ($\frac{K_{AMP,c}}{\frac{k_{+AMP}}{k_{+{AMP}^{'}}+k_{-AMP}}+1}$) is approximately equivalent to *K*_AMP,c_. With this approximation, *λ*_obs,AMP,slow_ becomes linear if *K*_AMP,c_ >>[M], i.e.:

$$\lambda_{obs, AMP,slow}\sim\frac{k_{-A{MP}^{'}}}{1+\frac{k_{+A{MP}^{'}}}{k_{-AMP}}}+k_{+AMP}k_{+A{MP}^{'}}\frac{\frac{k_{+{AMP}^{'}}+k_{-AMP}+k_{-A{MP}^{'}}}{k_{+{AMP}^{'}}+k_{-AMP}}}{K_{{AMP,}_{c}}\left( k_{+{AMP}^{'}}+k_{-AMP} \right)}\left[ M \right]$$

$$\begin{aligned} =\frac{k_{-{AMP}^{'}}}{1+\frac{k_{+A{MP}^{'}}}{k_{-AMP}}}+\frac{1+\frac{k_{-A{MP}^{'}}}{k_{+A{MP}^{'}}+k_{-AMP}}}{1+\frac{k_{-AMP}}{k_{+A{MP}^{'}}}}\frac{k_{+AMP}}{K_{A{MP,}_{c}}}\left[ M \right]\#S26 \end{aligned}$$

***Case 2 –*** ${\boldsymbol{k}_{\boldsymbol{+AMP}}\boldsymbol{\gg k}}_{\boldsymbol{+}\boldsymbol{AMP}^{\boldsymbol{'}}}\boldsymbol{+}\boldsymbol{k}_{\boldsymbol{-AMP}}$ ***– constant λ_obs,AMP,slow_***

When ${k_{+AMP}\gg k}_{+A{MP}^{'}}+k_{-AMP}$ and the [AMP] used is much larger than the midpoint, (i.e. $\frac{K_{{AMP,}_{c}}}{1+\frac{k_{+AMP}}{k_{+{AMP}^{'}}+k_{-AMP}}}\ll\left[ M \right]$), *λ*_obs,AMP,slow_ reaches a maximum and remains constant, i.e.;

$$\lambda_{obs, AMP,slow}\sim\frac{k_{-A{MP}^{'}}}{1+\frac{k_{+A{MP}^{'}}}{k_{-AMP}}}+k_{+AMP}k_{+A{MP}^{'}}\frac{k_{+{AMP}^{'}}+k_{-AMP}+k_{-{AMP}^{'}}}{\left( k_{+AMP}+k_{+{AMP}^{'}}+k_{-AMP} \right)\left( k_{+A{MP}^{'}}+k_{-AMP} \right)}$$

$$\begin{aligned} =\frac{k_{-{AMP}^{'}}}{1+\frac{k_{+{AMP}^{'}}}{k_{-AMP}}}+\frac{k_{+A{MP}^{'}}+k_{-A{MP}^{'}}+k_{-AMP}}{\left( 1+\frac{k_{-AMP}}{k_{+A{MP}^{'}}} \right)\left( 1+\frac{k_{+A{MP}^{'}}+k_{-AMP}}{k_{+AMP}} \right)}<k_{+A{MP}^{'}}+2k_{-A{MP}^{'}}+k_{-AMP}\#S27 \end{aligned}$$

1. **AMP irreversible dissociation (Fig. 5)**

In AMP irreversible dissociation experiments, excess competing nucleotide saturates any free ENPP1 ([E]=0) ensuring no rebinding of released AMP, so that *k*_+AMP_ approximates zero and Eq. S2 becomes:

$$\begin{aligned} \frac{d\left[ E\cdot M \right]}{dt}=-\left( k_{+{AMP}^{'}}+k_{-AMP} \right)\left[ E\cdot M \right]+k_{-{AMP}^{'}}\left[ E{\cdot M}^{'} \right]\#S28 \end{aligned}$$

Differential Eq. S3 ($\frac{d[E\cdot M']}{dt}$) is unchanged under these circumstances. The eigenfunction for the system of differential equations comprised of Eqs. S28 and S3 is:

$$\left| \begin{matrix} -\lambda+k_{+{AMP}^{'}}+k_{-AMP} & -k_{-{AMP}^{'}} \\ -k_{+{AMP}^{'}} & -\lambda+k_{-{AMP}^{'}} \end{matrix} \right|=\left( -\lambda+k_{+{AMP}^{'}}+k_{-AMP} \right)\left( -\lambda+k_{-A{MP}^{'}} \right)-k_{+A{MP}^{'}}k_{-A{MP}^{'}}$$

$$=\lambda^{2}-\left( k_{+A{MP}^{'}}+k_{-A{MP}^{'}}+k_{-AMP} \right)\lambda+\left( k_{+A{MP}^{'}}+k_{-AMP} \right)k_{-A{MP}^{'}}-k_{+{AMP}^{'}}k_{-{AMP}^{'}}$$

$$\begin{aligned} =\lambda^{2}-\left( k_{+A{MP}^{'}}+k_{-{AMP}^{'}}+k_{-AMP} \right)\lambda+k_{-A{MP}^{'}}k_{-AMP}=0\#S29 \end{aligned}$$

The two eigenvalues of Eq. S29 (*λ*_obs,AMPdiss,fast_ and *λ*_obs,AMPdiss,slow_) are:

$$\begin{aligned} \lambda_{obs, AMPdiss,fast},\lambda_{obs, AMPdiss,slow}=\frac{k_{+{AMP}^{'}}+k_{-A{MP}^{'}}+k_{-AMP}\pm\sqrt{\left( k_{+{AMP}^{'}}+k_{-{AMP}^{'}}+k_{-AMP} \right)^{2}-4k_{-A{MP}^{'}}k_{-AMP}}}{2}\#S30 \end{aligned}$$

or

$$\begin{aligned} =\frac{k_{+{AMP}^{'}}+k_{-A{MP}^{'}}+k_{-AMP}\pm\sqrt{\left( k_{+A{MP}^{'}}-k_{-{AMP}^{'}}+k_{-AMP} \right)^{2}+4k_{+{AMP}^{'}}k_{-{AMP}^{'}}}}{2}\#S31 \end{aligned}$$

or

$$\begin{aligned} =\frac{k_{+A{MP}^{'}}+k_{-A{MP}^{'}}+k_{-AMP}\pm\sqrt{\left( k_{+A{MP}^{'}}+k_{-A{MP}^{'}}-k_{-AMP} \right)^{2}+4k_{+A{MP}^{'}}k_{-AMP}}}{2}\#S32 \end{aligned}$$

According to Eqs. S30-S32, the **fast** observed rate constant for AMP release is

$$\frac{k_{+A{MP}^{'}}+k_{-A{MP}^{'}}+k_{-AMP}+\sqrt{\left( k_{+{AMP}^{'}}+k_{-{AMP}^{'}}+k_{-AMP} \right)^{2}}}{2}>\lambda_{obs, AMPdiss,fast}>\left\{ \begin{aligned} \frac{k_{+{AMP}^{'}}+k_{-A{MP}^{'}}+k_{-AMP}\pm\sqrt{\left( k_{+{AMP}^{'}}-k_{-A{MP}^{'}}+k_{-AMP} \right)^{2}}}{2}, \mathrm{if}k_{-AMP}>k_{-{AMP}^{'}} \\ \frac{k_{+{AMP}^{'}}+k_{-A{MP}^{'}}+k_{-AMP}\pm\sqrt{\left( k_{+A{MP}^{'}}+k_{-A{MP}^{'}}-k_{-AMP} \right)^{2}}}{2}, \mathrm{if}k_{-AMP}<k_{-{AMP}^{'}} \end{aligned} \right.$$

i.e.

$$\begin{aligned} k_{+A{MP}^{'}}+k_{-A{MP}^{'}}+k_{-AMP}>\lambda_{obs, AMPdiss,fast}>\left\{ \begin{aligned} k_{+{AMP}^{'}}+k_{-AMP}, \mathrm{if}k_{-AMP}>k_{-A{MP}^{'}} \\ k_{+A{MP}^{'}}+k_{-{AMP}^{'}}, \mathrm{if}k_{-AMP}<k_{-A{MP}^{'}} \end{aligned} \right.\#S33 \end{aligned}$$

In assigning a lower limit for *λ*_obs,AMPdiss,fast_, (*k*_+AMP′_ + (*k*_−AMP_ or *k*_−AMP′_)) the second term is whichever value, *k*_−AMP_ or *k*_−AMP′_, is larger in order to narrow the range of possible *λ*_obs,AMPdiss,fast_ values.

According to Eq. S30, the **slow** observed rate constant is

$$\begin{aligned} \lambda_{obs, AMPdiss,slow}=\frac{\lambda_{obs, AMPdiss,fast}\lambda_{obs, AMPdiss,slow}}{\lambda_{obs, AMPdiss,fast}}=\frac{k_{-{AMP}^{'}}k_{-AMP}}{\lambda_{obs, AMPdiss,fast}}\#S34 \end{aligned}$$

Applying the approximations of *λ*_obs,AMPdiss,fast_ from Eq. S33 to Eq. S34 yields:

$$\begin{aligned} \left\{ \begin{aligned} \frac{k_{-A{MP}^{'}}k_{-AMP}}{k_{+{AMP}^{'}}+k_{-AMP'}}, \mathrm{if}k_{-AMP}<k_{-{AMP}^{'}} \\ \frac{k_{-A{MP}^{'}}k_{-AMP}}{k_{+A{MP}^{'}}+k_{-AMP}}, \mathrm{if}k_{-AMP}>k_{-A{MP}^{'}} \end{aligned} \right.>\lambda_{obs, AMPdiss,slow}>\frac{k_{-A{MP}^{'}}k_{-AMP}}{k_{+{AMP}^{'}}+k_{-A{MP}^{'}}+k_{-AMP}}\#S35 \end{aligned}$$

In assigning an upper limit for *λ*_obs,AMPdiss,slow_, ($\frac{k_{-A{MP}^{'}}k_{-AMP}}{k_{+{AMP}^{'}}+\left( k_{-AMP'} or k_{-AMP} \right)}$) the second term in the denominator is whichever value, *k*_−AMP′_ or *k*_−AMP_, is larger in order to narrow the range of possible *λ*_obs,AMPdiss,slow_ values.

1. ***Steady state AMP production*** ***(Fig. 1)***

When ENPP1 ATPase cycling reaches steady state, the concentrations of all intermediate species [E], [E∙M] and [E∙M’] remain constant for as long as product formation remains linear. During the initial linear phase of AMP accumulation, [M] ≈0 and the rate of product inhibition is negligible. Consequently, we can assume [(E∙M)] ≈ 0. Substituting Eq. S4 into Eq. S7 using these assumptions yields:

$$0=\frac{d\left( \left[ E\cdot T \right]+\left[ E\cdot M \right] \right)}{dt}=k_{+T}\left[ (E\cdot T) \right]-\left( k_{+{AMP}^{'}}+k_{-AMP} \right)\left[ E\cdot M \right]+k_{-A{MP}^{'}}\left[ E\cdot M^{'} \right]+k_{+AMP}\left[ (E\cdot M) \right]$$

$$\begin{aligned} \sim k_{+T}\frac{\left[ E \right]\left[ T \right]}{K_{T,c}}-\left( k_{+A{MP}^{'}}+k_{-AMP} \right)\left[ E\cdot M \right]+k_{-{AMP}^{'}}\left[ E\cdot M^{'} \right]\#S36 \end{aligned}$$

Eq. S6 (mass conservation) becomes:

$$E_{0}=\left[ E \right]+\frac{\left[ E \right][T]}{K_{T,c}}+\left[ E\cdot M \right]+\left[ E{\cdot M}^{'} \right]$$

$$=\left[ E \right]\left( 1+\frac{[T]}{K_{T,c}} \right)+\left[ E\cdot M \right]+\left[ E{\cdot M}^{'} \right]$$

i.e.,

$$\begin{aligned} \left[ E \right]=\frac{K_{T,c}\left( E_{0}-\left[ E\cdot M \right]-\left[ E\cdot M^{'} \right] \right)}{K_{T,c}+\left[ T \right]}\#S37 \end{aligned}$$

Substituting Eq. S37 into Eq. S36 and rearranging terms yields:

$$\frac{k_{+T}\left[ T \right]\left( E_{0}-\left[ E\cdot M \right]-\left[ E{\cdot M}^{'} \right] \right)}{K_{T,c}+\left[ T \right]}-\left( k_{+A{MP}^{'}}+k_{-AMP} \right)\left[ E\cdot M \right]+k_{-{AMP}^{'}}\left[ E{\cdot M}^{'} \right]$$

$$=\frac{k_{+T}\left[ T \right]E_{0}}{K_{T,c}+\left[ T \right]}-\left( \frac{k_{+T}\left[ T \right]}{K_{T,c}+\left[ T \right]}+k_{+{AMP}^{'}}+k_{-AMP} \right)\left[ E\cdot M \right]-\left( \frac{k_{+T}\left[ T \right]}{K_{T,c}+\left[ T \right]}-k_{-A{MP}^{'}} \right)\left[ E\cdot M^{'} \right]=0$$

i.e.,

$$\left( k_{+T}\left[ T \right]+\left( k_{+{AMP}^{'}}+k_{-AMP} \right)\left( K_{T,c}+\left[ T \right] \right) \right)\left[ E\cdot M \right]+\left( k_{+T}\left[ T \right]-k_{-{AMP}^{'}}\left( K_{T,c}+\left[ T \right] \right) \right)\left[ E{\cdot M}^{'} \right]$$

$$=\left( \left( k_{+A{MP}^{'}}+k_{-AMP} \right)K_{T,c}+\left( k_{+T}+k_{+A{MP}^{'}}+k_{-AMP} \right)\left[ T \right] \right)\left[ E\cdot M \right]$$

$$\begin{aligned} \begin{aligned} +\left( -k_{-{AMP}^{'}}K_{T,c}+\left( k_{+T}-k_{-{AMP}^{'}} \right)\left[ T \right] \right)\left[ E{\cdot M}^{'} \right]=k_{+T}\left[ T \right]E_{0} \end{aligned}\#S38 \end{aligned}$$

During steady state cycling, Eq. S3 is:

$$0=\frac{d\left[ E{\cdot M}^{'} \right]}{dt}=k_{+A{MP}^{'}}\left[ E\cdot M \right]-k_{-{AMP}^{'}}\left[ E\cdot M^{'} \right]$$

i.e.,

$$\begin{aligned} \left[ E\cdot M^{'} \right]=\frac{k_{+{AMP}^{'}}}{k_{-{AMP}^{'}}}\left[ E\cdot M \right]\#S39 \end{aligned}$$

Substituting Eq. S39 into Eq. S38 and solving for [*E*$\cdot$*M*] yields:

$$\begin{aligned} \left[ E\cdot M \right]=\frac{k_{+T}k_{-A{MP}^{'}}E_{0}\left[ T \right]}{{k_{-{AMP}^{'}}k_{-AMP}K}_{T,c}+\left( {k_{-A{MP}^{'}}k}_{+T}+k_{-A{MP}^{'}}k_{-AMP}+k_{+T}k_{+{AMP}^{'}} \right)\left[ T \right]}\#S40 \end{aligned}$$

According to Scheme 2 and Eq. S40, the steady state rate of AMP product formation (*v*_0_) is:

$$\frac{d[M]}{dt}=k_{-AMP}\left[ E\cdot M \right]=\frac{k_{+T}k_{-A{MP}^{'}}k_{-AMP}E_{0}\left[ T \right]}{{k_{-{AMP}^{'}}k_{-AMP}K}_{T,c}+\left( {k_{-{AMP}^{'}}k}_{+T}+k_{-{AMP}^{'}}k_{-AMP}+k_{+T}k_{+A{MP}^{'}} \right)\left[ T \right]}$$

$$\begin{aligned} =\frac{\frac{k_{+T}k_{-A{MP}^{'}}k_{-AMP}}{{k_{-{AMP}^{'}}k}_{+T}+k_{-A{MP}^{'}}k_{-AMP}+k_{+T}k_{+{AMP}^{'}}}E_{0}\left[ T \right]}{\frac{{k_{-A{MP}^{'}}k_{-AMP}K}_{Tc}}{{k_{-{AMP}^{'}}k}_{+T}+k_{-{AMP}^{'}}k_{-AMP}+k_{+T}k_{+{AMP}^{'}}}+\left[ T \right]}\#S41 \end{aligned}$$

The initial velocity (*v*_0_) depends hyperbolically on [*T*] with an apparent *K*_M,T_ of:

$$\begin{aligned} K_{M,T}=\frac{k_{-A{MP}^{'}}k_{-AMP}K_{T,c}}{k_{+T}\left( k_{-A{MP}^{'}}+k_{+{AMP}^{'}} \right)+k_{-{AMP}^{'}}k_{-AMP}}\#S42 \end{aligned}$$

and a *k*_cat,T_ of:

$$\begin{aligned} k_{cat,T}=\frac{k_{+T}k_{-{AMP}^{'}}k_{-AMP}}{k_{+T}\left( k_{-{AMP}^{'}}+k_{+{AMP}^{'}} \right)+k_{-A{MP}^{'}}k_{-AMP}}=\frac{k_{-AMP}}{1+\frac{k_{+{AMP}^{'}}}{k_{-{AMP}^{'}}}+\frac{k_{-AMP}}{k_{+T}}}<k_{-AMP}\#S43 \end{aligned}$$

$$\begin{aligned} \frac{k_{cat,T}}{K_{M,T}}=\frac{k_{+T}}{K_{T,c}}\#S44 \end{aligned}$$

The specificity constant (*k*_cat,T_/*K*_M,T_) for ATP cleavage by ENPP1 is equivalent to the second order association rate constant for ATP binding, which we get from the slope of the linear approximation of the λ_obs,T,fast_ [T]-dependence (Eq. S11). The above derivation leading to Eq. S44 holds when progression through the cycle is more rapid than ATP dissociation (*k*_internal_ >> *k*_−T_). In cases where *k*_internal_ ≈ *k*_−T_, the value of *k*_cat,T_/*K*_M,T_ is equal to the second order ATP association rate constant (*k*_+T_/*K*_T,c_) multiplied by the probability of continuing through the cycle and thus, *k*_cat,T_/*K*_M,T_ ≤ *k*_+T_/*K*_T,c_ (15).

1. ***ENPP1 ATPase single turnover – quenched-flow experiment*** ***(Fig. 6A-C)***

The quenched-flow experiments simultaneously monitor time courses of substrate (ATP) depletion, product (AMP) formation, and nucleotidylated ENPP1−AMP intermediate formation and subsequent depletion. The measured ATP concentration includes both free and enzyme-bound ATP (i.e. [T]+[E∙T]). Similarly, the measured AMP concentration includes both free AMP and (non-covalently) enzyme-bound AMP (i.e. [E]+[E∙M]+[E∙M’]). The measured concentration of the nucleotidylated ENPP1−AMP intermediate includes both states before and after PP*_i_* release (i.e. [E−M∙PP_i_] + [E−M]).

From ATP single turnover experiments measured by stopped-flow (Fig. 2) and the AMP binding affinity (*K*_AMP,overall_; Table 1), we know that AMP product does not dissociate from ENPP1 under these conditions (E_0_ > T_0_; i.e. [M]≈0 during the reaction).

Under these conditions, the mass conservation equations for ATP and ENPP1 are given by:

$$T_{0}=\left[ T \right]+\left[ E\cdot T \right]+ \left[ E-MPP_{i} \right]+ \left[ E-M \right]+\left[ E\cdot M \right]+\left[ E\cdot M^{'} \right]+\left[ M \right]$$

$$\begin{aligned} \approx\left[ T \right]+\left[ E\cdot T \right]+ \left[ E-MPP_{i} \right]+ \left[ E-M \right]+\left[ E\cdot M \right]+\left[ E\cdot M^{'} \right]\#S45 \end{aligned}$$

$$E_{0}=\left[ E \right]+\left[ E\cdot T \right]+ \left[ E-MPP_{i} \right]+ \left[ E-M \right]+\left[ E\cdot M \right]+\left[ E\cdot M^{'} \right]$$

$$\begin{aligned} \approx\left[ E \right]+T_{0}-\left[ T \right]\#S46 \end{aligned}$$

The quenched-flow ATP single turnover experiments show ATP is rapidly and completely converted to AMP in less than 15 milliseconds (λ_obs_ >>250 sec^−1^, Fig. 6). This indicates that all biochemical transitions between the E∙T and E∙M states – initial ATP cleavage (*K*_cleavage_), PP*_i_* release (*K*_PP_*_i_*) and covalent intermediate hydrolysis (*K*_hydrolysis_) – are rapid and essentially irreversible, i.e.:

$$\begin{aligned} k_{-cleavage}, k_{+PP_{i}}, k_{-hydrolysis}\approx0 \left( {\mu M}^{-1} \right) s^{-1}\#S47 \end{aligned}$$

$$\begin{aligned} k_{+cleavage}, k_{-PP_{i}}, k_{+hydrolysis}\approx1000 s^{-1}\#S48 \end{aligned}$$

The experimental conditions and assumptions in Eq. S45-S48 are used throughout the following derivation.

The differential equation for ATP depletion is:

$$\begin{aligned} \frac{d\left[ T \right]}{dt}\sim-\frac{k_{+T}}{K_{T,c}}\left[ E \right]\left[ T \right]=-\frac{k_{+T}}{K_{T,c}}\left( E_{0}-T_{0}+\left[ T \right] \right)\left[ T \right]\#S49 \end{aligned}$$

Using ENPP1 mass conservation, the equation can be re-arranged to:

$$\frac{d\left[ T \right]}{\left( E_{0}-T_{0}+\left[ T \right] \right)\left[ T \right]}=\frac{1}{E_{0}-T_{0}}\left( -\frac{d\left[ T \right]}{E_{0}-T_{0}+\left[ T \right]}+\frac{d\left[ T \right]}{\left[ T \right]} \right)$$

$$=\frac{1}{E_{0}-T_{0}}\left( -d(ln(E_{0}-T_{0}+\left[ T \right])+d(ln\left[ T \right] \right)$$

$$\begin{aligned} =\frac{1}{E_{0}-T_{0}}d\ln\left( \frac{\left[ T \right]}{E_{0}-T_{0}+\left[ T \right]} \right)=-\frac{k_{+T}}{K_{T,c}}dt\#S50 \end{aligned}$$

Integrating both sides of the equation and incorporating the initial condition ([*T*] = *T*_0_ at *t* = 0), yields the following solution:

$$\frac{\left[ T \right]}{E_{0}-T_{0}+\left[ T \right]}=\frac{T_{0}}{E_{0}}e^{-\left( E_{0}-T_{0} \right)\frac{k_{+T}}{K_{T,c}}t}$$

i.e.,

$$\left[ T \right]=\frac{\left( E_{0}-T_{0} \right)T_{0}e^{-\left( E_{0}-T_{0} \right)\frac{k_{+T}}{K_{T,c}}t}}{E_{0}-T_{0}e^{-\left( E_{0}-T_{0} \right)\frac{k_{+T}}{K_{T,c}}t}}=-\frac{\left( E_{0}-T_{0} \right)\left( E_{0}-T_{0}e^{-\left( E_{0}-T_{0} \right)\frac{k_{+T}}{K_{T,c}}t}-E_{0} \right)}{E_{0}-T_{0}e^{-\left( E_{0}-T_{0} \right)\frac{k_{+T}}{K_{T,c}}t}}$$

$$=-\left( E_{0}-T_{0} \right)+\frac{E_{0}\left( E_{0}-T_{0} \right)}{E_{0}-T_{0}e^{-\left( E_{0}-T_{0} \right)\frac{k_{+T}}{K_{T,c}}t}}$$

$$=-\left( E_{0}-T_{0} \right)+\left( E_{0}-T_{0} \right)\left( 1+\frac{T_{0}}{E_{0}}e^{-\left( E_{0}-T_{0} \right)\frac{k_{+T}}{K_{T,c}}t}+\left( \frac{T_{0}}{E_{0}} \right)^{2}e^{-2\left( E_{0}-T_{0} \right)\frac{k_{+T}}{K_{T,c}}t}+\left( \frac{T_{0}}{E_{0}} \right)^{3}e^{-3\left( E_{0}-T_{0} \right)\frac{k_{+T}}{K_{T,c}}t}+O\left( \left( \frac{T_{0}}{E_{0}} \right)^{4}e^{-4\left( E_{0}-T_{0} \right)\frac{k_{+T}}{K_{T,c}}t} \right) \right)$$

$$=\left( E_{0}-T_{0} \right)\left( \frac{T_{0}}{E_{0}}e^{-\left( E_{0}-T_{0} \right)\frac{k_{+T}}{K_{T,c}}t}+\left( \frac{T_{0}}{E_{0}} \right)^{2}e^{-2\left( E_{0}-T_{0} \right)\frac{k_{+T}}{K_{T,c}}t}+\left( \frac{T_{0}}{E_{0}} \right)^{3}e^{-3\left( E_{0}-T_{0} \right)\frac{k_{+T}}{K_{T,c}}t}+O\left( \left( \frac{T_{0}}{E_{0}} \right)^{4}e^{-4\left( E_{0}-T_{0} \right)\frac{k_{+T}}{K_{T,c}}t} \right) \right)$$

$$\begin{aligned} =C_{1}e^{-\left( E_{0}-T_{0} \right)\frac{k_{+T}}{K_{T,c}}t}+C_{2}e^{-2\left( E_{0}-T_{0} \right)\frac{k_{+T}}{K_{T,c}}t}+C_{3}e^{-3\left( E_{0}-T_{0} \right)\frac{k_{+T}}{K_{T,c}}t}+O\left( e^{-4\left( E_{0}-T_{0} \right)\frac{k_{+T}}{K_{T,c}}t} \right)\#S51 \end{aligned}$$

Taylor expansion is used to expand $\frac{1}{1-\frac{T_{0}}{E_{0}}e^{-\left( E_{0}-T_{0} \right)\frac{k_{+T}}{K_{T,c}}t}}$ into a series of different power functions of $\frac{T_{0}}{E_{0}}e^{-\left( E_{0}-T_{0} \right)\frac{k_{+T}}{K_{T,c}}t}$ with the power order ranging from 0 to infinity. $\frac{T_{0}}{E_{0}}e^{-\left( E_{0}-T_{0} \right)\frac{k_{+T}}{K_{T,c}}t}<1$ guarantees the Taylor expansion converges, and we list only first 4 terms to the third order of power. In the expression, the coefficients *C_j_* are

$$\begin{aligned} C_{j}=\left( E_{0}-T_{0} \right)\left( \frac{T_{0}}{E_{0}} \right)^{j}, j=1, 2, 3\#S52 \end{aligned}$$

Since all coefficients are positive, Eq. S51 indicates [T] is the sum of exponential decays. With free ATP concentration expressed in Eq. S51, Eq. S49 can be re-written as:

$$-\frac{k_{+T}}{K_{T,c}}\left[ E \right]\left[ T \right]=\frac{d\left[ T \right]}{dt}=-\left( E_{0}-T_{0} \right)\frac{k_{+T}}{K_{T,c}}C_{1}e^{-\left( E_{0}-T_{0} \right)\frac{k_{+T}}{K_{T,c}}t}-2\left( E_{0}-T_{0} \right)\frac{k_{+T}}{K_{T,c}}C_{2}e^{-2\left( E_{0}-T_{0} \right)\frac{k_{+T}}{K_{T,c}}t}$$

$$\begin{aligned} -3\left( E_{0}-T_{0} \right)\frac{k_{+T}}{K_{T,c}}C_{3}e^{-3\left( E_{0}-T_{0} \right)\frac{k_{+T}}{K_{T,c}}t}+O\left( e^{-4\left( E_{0}-T_{0} \right)\frac{k_{+T}}{K_{T,c}}t} \right)\#S53 \end{aligned}$$

ATP cleavage is extremely fast (~1000 s^−1^) and essentially irreversible. The differential equation for E∙T formation and subsequent ATP cleavage is:

$$\frac{d\left[ E\cdot T \right]}{dt}=\frac{k_{+T}}{K_{T,c}}\left[ E \right]\left[ T \right]-\left( k_{+cleavage}+k_{-T} \right)\left[ E\cdot T \right]+k_{-cleavage}\left[ E-M\cdot PP_{i} \right]$$

$$\begin{aligned} \sim\frac{k_{+T}}{K_{T,c}}\left[ E \right]\left[ T \right]-\left( k_{+cleavage}+k_{-T} \right)\left[ E\cdot T \right]\#S54 \end{aligned}$$

i.e.,

$$\frac{d\left[ E\cdot T \right]}{dt}+\left( k_{+cleavage}+k_{-T} \right)\left[ E\cdot T \right]=\frac{k_{+T}}{K_{T,c}}\left[ E \right]\left[ T \right]$$

$$=\left( E_{0}-T_{0} \right)\frac{k_{+T}}{K_{T,c}}C_{1}e^{-\left( E_{0}-T_{0} \right)\frac{k_{+T}}{K_{T,c}}t}+2\left( E_{0}-T_{0} \right)\frac{k_{+T}}{K_{T,c}}C_{2}e^{-2\left( E_{0}-T_{0} \right)\frac{k_{+T}}{K_{T,c}}t}$$

$$\begin{aligned} +3\left( E_{0}-T_{0} \right)\frac{k_{+T}}{K_{T,c}}C_{3}e^{-3\left( E_{0}-T_{0} \right)\frac{k_{+T}}{K_{T,c}}t}+O\left( e^{-4\left( E_{0}-T_{0} \right)\frac{k_{+T}}{K_{T,c}}t} \right)\#S55 \end{aligned}$$

To solve differential equation Eq. S55, the method of variation of parameters is employed, and the solution is

$$\begin{aligned} \left[ E\cdot T \right]=P\left( t \right)e^{-\left( k_{+cleavage}+k_{-T} \right)t}\#S56 \end{aligned}$$

and the derivative of the unknown time function *P*(t) has to be

$$\frac{dP\left( t \right)}{dt}=\left( E_{0}-T_{0} \right)\frac{k_{+T}}{K_{T,c}}\left( C_{1}e^{-\left( E_{0}-T_{0} \right)\frac{k_{+T}}{K_{T,c}}t}+2C_{2}e^{-2\left( E_{0}-T_{0} \right)\frac{k_{+T}}{K_{T,c}}t} \right.$$

$$\begin{aligned} \left. +3C_{3}e^{-3\left( E_{0}-T_{0} \right)\frac{k_{+T}}{K_{T,c}}t}+O\left( e^{-4\left( E_{0}-T_{0} \right)\frac{k_{+T}}{K_{T,c}}t} \right) \right)e^{\left( k_{+cleavage}+k_{-T} \right)t}\#S57 \end{aligned}$$

Integrating Eq. S57 yields the general expression of the function *P*(t):

$$P\left( t \right)=A_{1}+\left( E_{0}-T_{0} \right)\frac{k_{+T}}{K_{T,c}}\frac{C_{1}}{k_{+cleavage}+k_{-T}-\left( E_{0}-T_{0} \right)\frac{k_{+T}}{K_{T,c}}}e^{-\left( E_{0}-T_{0} \right)\frac{k_{+T}}{K_{T,c}}t}$$

$$+\frac{2C_{2}}{k_{+cleavage}+k_{-T}-2\left( E_{0}-T_{0} \right)\frac{k_{+T}}{K_{T,c}}}e^{-2\left( E_{0}-T_{0} \right)\frac{k_{+T}}{K_{T,c}}t}$$

$$+\frac{{3C}_{3}}{k_{+cleavage}+k_{-T}-3\left( E_{0}-T_{0} \right)\frac{k_{+T}}{K_{T,c}}}e^{-3\left( E_{0}-T_{0} \right)\frac{k_{+T}}{K_{T,c}}t}\left. +O\left( e^{-4\left( E_{0}-T_{0} \right)\frac{k_{+T}}{K_{T,c}}t} \right) \right)e^{\left( k_{+cleavage}+k_{-T} \right)t}$$

$${=A}_{1}+\left( D_{1}e^{-\left( E_{0}-T_{0} \right)\frac{k_{+T}}{K_{T,c}}t}+D_{2}e^{-2\left( E_{0}-T_{0} \right)\frac{k_{+T}}{K_{T,c}}t}+D_{3}e^{-3\left( E_{0}-T_{0} \right)\frac{k_{+T}}{K_{T,c}}t} \right.$$

$$\begin{aligned} \left. +O\left( e^{-4\left( E_{0}-T_{0} \right)\frac{k_{+T}}{K_{T,c}}t} \right) \right)e^{\left( k_{+cleavage}+k_{-T} \right)t}\#S58 \end{aligned}$$

where the coefficients are

$$\begin{aligned} D_{j}=\frac{j\left( E_{0}-T_{0} \right)\frac{k_{+T}}{K_{T,c}}C_{j}}{k_{+cleavage}+k_{-T}-j\left( E_{0}-T_{0} \right)\frac{k_{+T}}{K_{T,c}}}, j=1, 2, 3\#S59 \end{aligned}.$$

The general solution of [E∙T] is:

$$\left[ E\cdot T \right]=P\left( t \right)e^{-\left( k_{+cleavage}+k_{-T} \right)t}$$

$$=A_{1}e^{-\left( k_{+cleavage}+k_{-T} \right)t}+D_{1}e^{-\left( E_{0}-T_{0} \right)\frac{k_{+T}}{K_{T,c}}t}+D_{2}e^{-2\left( E_{0}-T_{0} \right)\frac{k_{+T}}{K_{T,c}}t}$$

$$\begin{aligned} +D_{3}e^{-3\left( E_{0}-T_{0} \right)\frac{k_{+T}}{K_{T,c}}t}+O\left( e^{-4\left( E_{0}-T_{0} \right)\frac{k_{+T}}{K_{T,c}}t} \right)\#S60 \end{aligned}$$

The decay of [ATP] as monitored by quenched-flow (the sum of both free ATP and the ENPP1-bound state) can be expressed as:

$$\left[ T \right]+\left[ E\cdot T \right]= A_{1}e^{-\left( k_{+cleavage}+k_{-T} \right)t}+\left( C_{1}+D_{1} \right)e^{-\left( E_{0}-T_{0} \right)\frac{k_{+T}}{K_{T,c}}t}+\left( C_{2}+D_{2} \right)e^{-2\left( E_{0}-T_{0} \right)\frac{k_{+T}}{K_{T,c}}t}$$

$$\begin{aligned} +\left( C_{3}+D_{3} \right)e^{-3\left( E_{0}-T_{0} \right)\frac{k_{+T}}{K_{T,c}}t}+O\left( e^{-4\left( E_{0}-T_{0} \right)\frac{k_{+T}}{K_{T,c}}t} \right)\#S61 \end{aligned}$$

The coefficients *C_j_* (Eq. S52) are positive, and since $k_{+cleavage}+k_{-T}\gg\left( E_{0}-T_{0} \right)\frac{k_{+T}}{K_{T,c}}$, for at least the first few *j* values, the denominator of *D_j_* >0 in Eq. S59. Therefore, the expressed exponential terms in Eq. S61 are exponential decays, indicating ATP is depleted monotonically.

Similarly, the differential equation for pyrophosphate release is approximated to:

$$\begin{aligned} \frac{d\left[ E-M\cdot PP_{i} \right]}{dt}\sim k_{+cleavage}\left[ E\cdot T \right]-k_{-PP_{i}} \left[ E-M\cdot PP_{i} \right]\#S62 \end{aligned}$$

Using the method of variation of parameters (also known as variation of constants), the solution of Eq. S62 has the following form:

$$\begin{aligned} \left[ E-M\cdot PP_{i} \right]={P^{'}(t)}^{e^{-k_{-PP_{i}}t}}\#S63 \end{aligned}$$

and the unknown time function *P′*(*t*) satisfies the following differential equation:

$$\frac{dP^{'}\left( t \right)}{dt}=\sim k_{+cleavage}\left[ E\cdot T \right]e^{k_{-PP_{i}}t}$$

$$=k_{+cleavage}\left( A_{1}e^{-\left( k_{+cleavage}+k_{-T} \right)t}+D_{1}e^{-\left( E_{0}-T_{0} \right)\frac{k_{+T}}{K_{T,c}}t}+D_{2}e^{-2\left( E_{0}-T_{0} \right)\frac{k_{+T}}{K_{T,c}}t} \right.$$

$$\begin{aligned} \left. +D_{3}e^{-3\left( E_{0}-T_{0} \right)\frac{k_{+T}}{K_{T,c}}t}+O\left( e^{-4\left( E_{0}-T_{0} \right)\frac{k_{+T}}{K_{T,c}}t} \right) \right)e^{k_{-PP_{i}}t}\#S64 \end{aligned}$$

Integrating the equation and substituting into Eq. S63 yields:

$$\left[ E-M\cdot PP_{i} \right]=A_{2}e^{-k_{-PP_{i}}t}+k_{+cleavage}\left( \frac{A_{1}}{k_{-PP_{i}}-\left( k_{+cleavage}+k_{-T} \right)}e^{-\left( k_{+cleavage}+k_{-T} \right)t} \right.$$

$$+\frac{D_{1}}{k_{-PP_{i}}-\left( E_{0}-T_{0} \right)\frac{k_{+T}}{K_{T,c}}}e^{-\left( E_{0}-T_{0} \right)\frac{k_{+T}}{K_{T,c}}t}+\frac{D_{2}}{k_{-PP_{i}}-2\left( E_{0}-T_{0} \right)\frac{k_{+T}}{K_{T,c}}}e^{-2\left( E_{0}-T_{0} \right)\frac{k_{+T}}{K_{T,c}}t}$$

$$\left. +\frac{D_{3}}{k_{-PP_{i}}-3\left( E_{0}-T_{0} \right)\frac{k_{+T}}{K_{T,c}}}e^{-3\left( E_{0}-T_{0} \right)\frac{k_{+T}}{K_{T,c}}t}+O\left( e^{-4\left( E_{0}-T_{0} \right)\frac{k_{+T}}{K_{T,c}}t} \right) \right)$$

$$=A_{1}^{'}e^{-\left( k_{+cleavage}+k_{-T} \right)t}+A_{2}e^{-k_{-PP_{i}}t}+E_{1}e^{-\left( E_{0}-T_{0} \right)\frac{k_{+T}}{K_{T,c}}t}+E_{2}e^{-2\left( E_{0}-T_{0} \right)\frac{k_{+T}}{K_{T,c}}t}$$

$$\begin{aligned} +E_{3}e^{-3\left( E_{0}-T_{0} \right)\frac{k_{+T}}{K_{T,c}}t}+O\left( e^{-4\left( E_{0}-T_{0} \right)\frac{k_{+T}}{K_{T,c}}t} \right)\#S65 \end{aligned}$$

where the coefficients are

$$A_{1}^{'}=\frac{k_{+cleavage}A_{1}}{k_{-PP_{i}}-\left( k_{+cleavage}+k_{-T} \right)}$$

$$\begin{aligned} E_{j}=\frac{k_{+cleavage}D_{j}}{k_{-PP_{i}}-j\left( E_{0}-T_{0} \right)\frac{k_{+T}}{K_{T,c}}}, j=1, 2, 3\#S66 \end{aligned}$$

When the nucleotidylated E−M covalent bond is hydrolyzed, the differential equation is approximated to:

$$\begin{aligned} \frac{d\left[ E-M \right]}{dt}\sim k_{-PP_{i}} \left[ E-M\cdot PP_{i} \right]-k_{+hydrolysis}\left[ E-M \right]\#S67 \end{aligned}$$

The solution of Eq. S67 using the method of variation of parameters is

$$\begin{aligned} \left[ E-M \right]=P^{''\left( t \right)e^{-k_{+hydrolysis}t}}\#S68 \end{aligned}$$

and the unknown time function is

$$\frac{dP^{''}(t)}{dt}=\sim k_{-PP_{i}} \left[ E-M\cdot PP_{i} \right]e^{k_{+hydrolysis}t}$$

$$=k_{-PP_{i}}\left( A_{1}^{'}e^{-\left( k_{+cleavage}+k_{-T} \right)t}+A_{2}e^{-k_{-PP_{i}}t}+E_{1}e^{-\left( E_{0}-T_{0} \right)\frac{k_{+T}}{K_{T,c}}t}+E_{2}e^{-2\left( E_{0}-T_{0} \right)\frac{k_{+T}}{K_{T,c}}t} \right.$$

$$\begin{aligned} \left. +E_{3}e^{-3\left( E_{0}-T_{0} \right)\frac{k_{+T}}{K_{T,c}}t}+O\left( e^{-4\left( E_{0}-T_{0} \right)\frac{k_{+T}}{K_{T,c}}t} \right) \right)e^{k_{+hydrolysis}t}\#S69 \end{aligned}$$

Substituting the integration of Eq. S69 into Eq. S68 yields:

$$\left[ E-M \right]=A_{3}e^{-k_{+hydrolysis}t}+k_{-PP_{i}}\left( \frac{A_{1}^{'}}{k_{+hydrolysis}-\left( k_{+cleavage}+k_{-T} \right)}e^{-\left( k_{+cleavage}+k_{-T} \right)t}+\frac{A_{2}}{k_{+hydrolysis}-k_{-PP_{i}}}e^{-k_{-PP_{i}}t} \right.$$

$$+\frac{E_{1}}{k_{+hydrolysis}-\left( E_{0}-T_{0} \right)\frac{k_{+T}}{K_{T,c}}}e^{-\left( E_{0}-T_{0} \right)\frac{k_{+T}}{K_{T,c}}t}+\frac{E_{2}}{k_{+hydrolysis}-2\left( E_{0}-T_{0} \right)\frac{k_{+T}}{K_{T,c}}}e^{-2\left( E_{0}-T_{0} \right)\frac{k_{+T}}{K_{T,c}}t}$$

$$\left. +\frac{E_{3}}{k_{+hydrolysis}-3\left( E_{0}-T_{0} \right)\frac{k_{+T}}{K_{T,c}}}e^{-3\left( E_{0}-T_{0} \right)\frac{k_{+T}}{K_{T,c}}t}+O\left( e^{-4\left( E_{0}-T_{0} \right)\frac{k_{+T}}{K_{T,c}}t} \right) \right)$$

$$=A_{1}^{''}e^{-\left( k_{+cleavage}+k_{-T} \right)t}+A_{2}^{'}e^{-k_{-PP_{i}}t}+A_{3}e^{-k_{+hydrolysis}t}$$

$$\begin{aligned} +F_{1}e^{-\left( E_{0}-T_{0} \right)\frac{k_{+T}}{K_{T,c}}t}+F_{2}e^{-2\left( E_{0}-T_{0} \right)\frac{k_{+T}}{K_{T,c}}t}+F_{3}e^{-3\left( E_{0}-T_{0} \right)\frac{k_{+T}}{K_{T,c}}t}+O\left( e^{-4\left( E_{0}-T_{0} \right)\frac{k_{+T}}{K_{T,c}}t} \right)\#S70 \end{aligned}$$

where the coefficients are

$$A_{1}^{''}=\frac{k_{-PP_{i}}A_{1}^{'}}{k_{+hydrolysis}-\left( k_{+cleavage}+k_{-T} \right)}$$

$$A_{2}^{'}=\frac{k_{-PP_{i}}A_{2}}{k_{+hydrolysis}-k_{-PP_{i}}}$$

$$\begin{aligned} F_{j}=\frac{k_{-PP_{i}}E_{j}}{k_{+hydrolysis}-j\left( E_{0}-T_{0} \right)\frac{k_{+T}}{K_{T,c}}}, j=1, 2, 3\#S71 \end{aligned}$$

The total concentration of the nucleotidylated E−M species monitored by quenched-flow is the sum of both nucleotidylated species, with and without bound PP*_i_* (Eq. 65 and 70):

$$\left[ E-M\cdot PP_{i} \right]+\left[ E-M \right]=\left( {A_{1}^{'}+A}_{1}^{''} \right)e^{-\left( k_{+cleavage}+k_{-T} \right)t}+\left( A_{2}+A_{2}^{'} \right)e^{-k_{-PP_{i}}t}+A_{3}e^{-k_{+hydrolysis}t}$$

$$+\left( E_{1}+F_{1} \right)e^{-\left( E_{0}-T_{0} \right)\frac{k_{+T}}{K_{T,c}}t}+\left( E_{2}+F_{2} \right)e^{-2\left( E_{0}-T_{0} \right)\frac{k_{+T}}{K_{T,c}}t}+\left( E_{3}+F_{3} \right)e^{-3\left( E_{0}-T_{0} \right)\frac{k_{+T}}{K_{T,c}}t}$$

$$\begin{aligned} +O\left( e^{-4\left( E_{0}-T_{0} \right)\frac{k_{+T}}{K_{T,c}}t} \right)\#S72 \end{aligned}$$

The initial coefficients *A*_1_ (Eq. S58), *A*_3_ (Eq. S65) and *A*_3_ (Eq. S70) have to be determined by the initial conditions. However, the first 3 exponential terms in Eq. S72 describe the depletion of a previous state to populate the [E∙T], [E−M∙PP*_i_*], and [E−M] states and therefore the coefficients for these three terms here should be negative so the exponential increases with time. Since $k_{-PP_{i}}\gg\left( E_{0}-T_{0} \right)\frac{k_{+T}}{K_{T,c}}$ and $k_{+hydrolysis}\gg\left( E_{0}-T_{0} \right)\frac{k_{+T}}{K_{T,c}}$, for smaller *j* values (≤3), the denominators of the coefficients *E_j_* (Eq. S66) and *F_j_* (Eq. S71) are positive, so that *E_j_* and *F_j_* in Eq. S72 are positive and yield an exponential decay of total E−M.

In a quenched-flow assay, the measured total [AMP] at any given time point consists of all free and non-covalently bound AMP (i.e. [M] + [E∙M] + [E∙M′]). The differential equation describing the time dependent change in this population is

$$\begin{aligned} \frac{d\left( \left[ M \right]+\left[ E\cdot M \right]+[E\cdot M^{'}] \right)}{dt}\sim k_{+hydrolysis}\left[ E\cdot M \right] \end{aligned}$$

$$=k_{+hydrolysis}\left( A_{1}^{''}e^{-\left( k_{+cleavage}+k_{-T} \right)t}+A_{2}^{'}e^{-k_{-PP_{i}}t}+A_{3}e^{-k_{+hydrolysis}t} \right.$$

$$\begin{aligned} \left. +F_{1}e^{-\left( E_{0}-T_{0} \right)\frac{k_{+T}}{K_{T,c}}t}+F_{2}e^{-2\left( E_{0}-T_{0} \right)\frac{k_{+T}}{K_{T,c}}t}+F_{3}e^{-3\left( E_{0}-T_{0} \right)\frac{k_{+T}}{K_{T,c}}t}+O\left( e^{-4\left( E_{0}-T_{0} \right)\frac{k_{+T}}{K_{T,c}}t} \right) \right)\#S73 \end{aligned}$$

Integrating the equation yields

$$\left[ M \right]+\left[ E\cdot M \right]+\left[ E\cdot M^{'} \right]$$

$$=constant-k_{+hydrolysis}\left( \frac{A_{1}^{''}}{k_{+cleavage}+k_{-T}}e^{-\left( k_{+cleavage}+k_{-T} \right)t}+\frac{A_{2}^{'}}{k_{-PP_{i}}}e^{-k_{-PP_{i}}t}+\frac{A_{3}}{k_{+hydrolysis}}e^{-k_{+hydrolysis}t} \right.$$

$$+\frac{F_{1}}{\left( E_{0}-T_{0} \right)\frac{k_{+T}}{K_{T,c}}}e^{-\left( E_{0}-T_{0} \right)\frac{k_{+T}}{K_{T,c}}t}+\frac{F_{2}}{2\left( E_{0}-T_{0} \right)\frac{k_{+T}}{K_{T,c}}}e^{-2\left( E_{0}-T_{0} \right)\frac{k_{+T}}{K_{T,c}}t}$$

$$\left. +\frac{F_{3}}{3\left( E_{0}-T_{0} \right)\frac{k_{+T}}{K_{T,c}}}e^{-3\left( E_{0}-T_{0} \right)\frac{k_{+T}}{K_{T,c}}t}+O\left( e^{-4\left( E_{0}-T_{0} \right)\frac{k_{+T}}{K_{T,c}}t} \right) \right)$$

$$={T_{0}-A}_{1}^{'''}e^{-\left( k_{+cleavage}+k_{-T} \right)t}-A_{2}^{''}e^{-k_{-PP_{i}}t}-A_{3}^{'}e^{-k_{+hydrolysis}t}-G_{1}e^{-\left( E_{0}-T_{0} \right)\frac{k_{+T}}{K_{T,c}}t}$$

$$\begin{aligned} -G_{2}e^{-2\left( E_{0}-T_{0} \right)\frac{k_{+T}}{K_{T,c}}t}-G_{3}e^{-3\left( E_{0}-T_{0} \right)\frac{k_{+T}}{K_{T,c}}t}+O\left( e^{-4\left( E_{0}-T_{0} \right)\frac{k_{+T}}{K_{T,c}}t} \right)\#S74 \end{aligned}$$

where the coefficients are

$$constant= T_{0}$$

$$A_{1}^{'''}=\frac{k_{+hydrolysis}A_{1}^{''}}{k_{+cleavage}+k_{-T}}$$

$$A_{2}^{''}=\frac{k_{+hydrolysis}A_{2}^{'}}{k_{-PP_{i}}}$$

$$A_{3}^{'}=\frac{k_{+hydrolysis}A_{3}}{k_{+hydrolysis}}=A_{3}$$

$$\begin{aligned} G_{j}=\frac{k_{+hydrolysis}F_{j}}{j\left( E_{0}-T_{0} \right)\frac{k_{+T}}{K_{T,c}}}, j=1, 2, 3\#S75 \end{aligned}$$

The integration constant (=*T_0_*) in Eq. S74 is determined by the condition at $\left. \left[ M \right]+\left[ E\cdot M \right]+\left[ E\cdot M^{'} \right] \right|_{t=\infty}=T_{0}$. Because *G_j_* values are proportional to *F_j_* in Eq. S71 and are positive for smaller *j* values, the exponential terms including *G_j_* in Eq. S75 increase with time.

The rate constants *k*_+cleavage_, *k*_−PP_*_i_*, and *k*_+hydrolysis_ are rapid (≈1000 sec^−1^), such that these transitions are completed within a few milliseconds and the exponential terms associated with these rate constants in Eq. S61, S72, and S74 can be ignored. The equations representing time-dependent concentration of the three monitored populations (ATP, ENPP1−AMP, and AMP) are therefore dominated by the same exponential terms with *λ*_obs_ of $\left( E_{0}-T_{0} \right)\frac{k_{+T}}{K_{T,c}}$, $2\left( E_{0}-T_{0} \right)\frac{k_{+T}}{K_{T,c}}$, and $3\left( E_{0}-T_{0} \right)\frac{k_{+T}}{K_{T,c}}$, and can be globally fit to a sum of exponentials with shared rate constants and individual amplitudes. Taking experimental conditions into account, the only exponential term observable on the relevant time scale would be the term with the smallest rate constant (*λ*_obs_ = $\left( E_{0}-T_{0} \right)\frac{k_{+T}}{K_{T,c}}$) as this value is already ≈300 sec^−1^ (E_0_ – T_0_ = 3 µM, $\frac{k_{+T}}{K_{T,c}}$ ≈ 100 µM^−1^ sec^−1^). This is in agreement with our experimental observations, as all three time traces fit well to a single exponential function with a shared *λ*_obs_ of 254 ± 26 sec^−1^.

1. ***ENPP1 ATPase multi-turnover – quenched-flow experiment*** ***(Fig.6D-E)***

In a multi-turnover experiment, the initial [ATP] is much greater than [ENPP1] (T_0_ >> E_0_) such that [ATP] can be treated as a constant ([T] ≈ T_0_ − E_0_) during the reaction. In our multi-turnover experiment, T_0_ = 60 μM and E_0_ = 4 μM. The pseudo first-order rate constant for ATP binding ($\frac{k_{+T}T_{0}}{K_{T,c}}$) is therefore ≈ 100×60 = 6000 sec^−1^. Since this value is significantly larger than *k*_+internal_ (≈1000 sec^−1^), it can be assumed that the ATP-bound state (E∙T) reaches a steady state value instantaneously ($\frac{d[E\cdot T]}{dt}=0$). Using mass conservation (Eq. S4) and the equilibrium equation for *K*_T,c_ (Eq. S6) to solve for [(E∙T)],

$$E_{0}=\left[ E \right]+\left[ (E\cdot T) \right]+\left[ E\cdot T \right]+\left[ E\cdot M \right]+\left[ E\cdot M^{'} \right]+\left[ (E\cdot M) \right]$$

$$\sim\frac{K_{T,c}\left[ (E\cdot T) \right]}{\left[ T \right]}+\left[ (E\cdot T) \right]+\left[ E\cdot T \right]+\left[ E\cdot M \right]+\left[ E{\cdot M}^{'} \right]$$

$$=\left[ (E\cdot T) \right]\frac{K_{T,c}+\left[ T \right]}{[T]}+\left[ E\cdot T \right]+\left[ E\cdot M \right]+\left[ E\cdot M^{'} \right]$$

i.e.,

$$\begin{aligned} \left[ (E\cdot T) \right]=\frac{E_{0}-\left[ E\cdot T \right]-\left[ E\cdot M \right]-\left[ E{\cdot M}^{'} \right]}{K_{T,c}+\left[ T \right]}[T]\#S76 \end{aligned}.$$

Eq. S1 becomes:

$$\frac{d\left[ E\cdot T \right]}{dt}=k_{+T}\frac{E_{0}-\left[ E\cdot T \right]-\left[ E\cdot M \right]-\left[ E\cdot M^{'} \right]}{K_{T,c}+\left[ T \right]}[T]-\left( k_{-T}+k_{+internal} \right)\left[ E\cdot T \right]+k_{-internal}\left[ E\cdot M \right]$$

$$\sim\frac{k_{+T}\left[ T \right]E_{0}}{K_{T,c}+\left[ T \right]}-\left( \frac{k_{+T}\left[ T \right]}{K_{T,c}+\left[ T \right]}+k_{-T}+k_{+internal} \right)\left[ E\cdot T \right]-\frac{k_{+T}\left[ T \right]}{K_{T,c}+\left[ T \right]}\left[ E\cdot M \right]$$

$$\begin{aligned} -\frac{k_{+T}\left[ T \right]}{K_{T,c}+\left[ T \right]}\left[ E\cdot M^{'} \right]=0\#S77 \end{aligned}$$

Rearranging terms yields

$$\begin{aligned} \left[ E\cdot T \right]=\frac{E_{0}-\left[ E\cdot M \right]-\left[ E\cdot M^{'} \right]}{\frac{k_{+T}\left[ T \right]}{K_{T,c}+\left[ T \right]}+k_{-T}+k_{+internal}}\frac{k_{+T}\left[ T \right]}{K_{T,c}+\left[ T \right]}\#S78 \end{aligned}$$

Substituting this equation into Eq. S2 yields

$$\frac{d\left[ E\cdot M \right]}{dt}=k_{+internal}\left[ E\cdot T \right]-\left( k_{-internal}+k_{+A{MP}^{'}}+k_{-AMP} \right)\left[ E\cdot M \right]+k_{-A{MP}^{'}}\left[ E{\cdot M}^{'} \right]+k_{+AMP}\left[ (E\cdot M) \right]$$

$$\sim k_{+internal}\frac{E_{0}-\left[ E\cdot M \right]-\left[ E{\cdot M}^{'} \right]}{\frac{k_{+T}\left[ T \right]}{K_{T,c}+\left[ T \right]}+k_{-T}+k_{+internal}}\frac{k_{+T}\left[ T \right]}{K_{T,c}+\left[ T \right]}-\left( k_{+A{MP}^{'}}+k_{-AMP} \right)\left[ E\cdot M \right]+k_{-{AMP}^{'}}\left[ E{\cdot M}^{'} \right]$$

$$=\frac{k_{+internal}\frac{k_{+T}\left[ T \right]E_{0}}{K_{T,c}+\left[ T \right]}}{\frac{k_{+T}\left[ T \right]}{K_{T,c}+\left[ T \right]}+k_{-T}+k_{+internal}}-\left( \frac{k_{+internal}\frac{k_{+T}\left[ T \right]}{K_{T,c}+\left[ T \right]}}{\frac{k_{+T}\left[ T \right]}{K_{T,c}+\left[ T \right]}+k_{-T}+k_{+internal}}+k_{+A{MP}^{'}}+k_{-AMP} \right)\left[ E\cdot M \right]$$

$$\begin{aligned} -\left( \frac{k_{+internal}\frac{k_{+T}\left[ T \right]}{K_{T,c}+\left[ T \right]}}{\frac{k_{+T}\left[ T \right]}{K_{T,c}+\left[ T \right]}+k_{-T}+k_{+internal}}-k_{-{AMP}^{'}} \right)\left[ E\cdot M^{'} \right]\#S79 \end{aligned}$$

To solve the system of differential equations comprised of Eq. S79 and S3, we calculate their eigenfunction

$$\left| \begin{matrix} -\lambda+\frac{k_{+internal}\frac{k_{+T}\left[ T \right]}{K_{T,c}+\left[ T \right]}}{\frac{k_{+T}\left[ T \right]}{K_{T,c}+\left[ T \right]}+k_{-T}+k_{+internal}}+k_{+M^{'}}+k_{-M} & \frac{k_{+internal}\frac{k_{+T}\left[ T \right]}{K_{T,c}+\left[ T \right]}}{\frac{k_{+T}\left[ T \right]}{K_{T,c}+\left[ T \right]}+k_{-T}+k_{+internal}}-k_{-A{MP}^{'}} \\ -k_{+{AMP}^{'}} & -\lambda+k_{-A{MP}^{'}} \end{matrix} \right|$$

$$=\left( -\lambda+\frac{k_{+internal}\frac{k_{+T}\left[ T \right]}{K_{T,c}+\left[ T \right]}}{\frac{k_{+T}\left[ T \right]}{K_{T,c}+\left[ T \right]}+k_{-T}+k_{+internal}}+k_{+{AMP}^{'}}+k_{-AMP} \right)\left( -\lambda+k_{-{AMP}^{'}} \right)$$

$$+k_{+A{MP}^{'}}\left( \frac{k_{+internal}\frac{k_{+T}\left[ T \right]}{K_{T,c}+\left[ T \right]}}{\frac{k_{+T}\left[ T \right]}{K_{T,c}+\left[ T \right]}+k_{-T}+k_{+internal}}-k_{-{AMP}^{'}} \right)$$

$$=\lambda^{2}-\left( \frac{k_{+internal}\frac{k_{+T}\left[ T \right]}{K_{T,c}+\left[ T \right]}}{\frac{k_{+T}\left[ T \right]}{K_{T,c}+\left[ T \right]}+k_{-T}+k_{+internal}}+k_{+A{MP}^{'}}+k_{-{AMP}^{'}}+k_{-AMP} \right)\lambda$$

$$+k_{-{AMP}^{'}}\left( \frac{k_{+internal}\frac{k_{+T}\left[ T \right]}{K_{Tc}+\left[ T \right]}}{\frac{k_{+T}\left[ T \right]}{K_{Tc}+\left[ T \right]}+k_{-T}+k_{+internal}}+k_{+A{MP}^{'}}+k_{-AMP} \right)$$

$$+k_{+{AMP}^{'}}\left( \frac{k_{+internal}\frac{k_{+T}\left[ T \right]}{K_{T,c}+\left[ T \right]}}{\frac{k_{+T}\left[ T \right]}{K_{T,c}+\left[ T \right]}+k_{-T}+k_{+internal}}-k_{-{AMP}^{'}} \right)$$

$$=\lambda^{2}-\left( \frac{k_{+internal}\frac{k_{+T}\left[ T \right]}{K_{T,c}+\left[ T \right]}}{\frac{k_{+T}\left[ T \right]}{K_{T,c}+\left[ T \right]}+k_{-T}+k_{+internal}}+k_{+{AMP}^{'}}+k_{-{AMP}^{'}}+k_{-AMP} \right)\lambda$$

$$\begin{aligned} +\left( \frac{\left( k_{+{AMP}^{'}}+k_{-A{MP}^{'}} \right)k_{+internal}\frac{k_{+T}\left[ T \right]}{K_{T,c}+\left[ T \right]}}{\frac{k_{+T}\left[ T \right]}{K_{T,c}+\left[ T \right]}+k_{-T}+k_{+internal}}+k_{-A{MP}^{'}}k_{-AMP} \right)=0\#S80 \end{aligned}$$

The two roots of the quadratic equation are the rate constants of the two observed exponential phases:

$$\lambda_{\pm}=\frac{1}{2}\left( \frac{k_{+internal}\frac{k_{+T}\left[ T \right]}{K_{T,c}+\left[ T \right]}}{\frac{k_{+T}\left[ T \right]}{K_{T,c}+\left[ T \right]}+k_{-T}+k_{+internal}}+k_{+{AMP}^{'}}+k_{-A{MP}^{'}}+k_{-AMP} \right.$$

$$\pm\sqrt{\left( \frac{k_{+internal}\frac{k_{+T}\left[ T \right]}{K_{T,c}+\left[ T \right]}}{\frac{k_{+T}\left[ T \right]}{K_{T,c}+\left[ T \right]}+k_{-T}+k_{+internal}}+k_{+A{MP}^{'}}+k_{-{AMP}^{'}}+k_{-AMP} \right)^{2}-4\left( \frac{\left( k_{+{AMP}^{'}}+k_{-{AMP}^{'}} \right)k_{+internal}\frac{k_{+T}\left[ T \right]}{K_{T,c}+\left[ T \right]}}{\frac{k_{+T}\left[ T \right]}{K_{T,c}+\left[ T \right]}+k_{-T}+k_{+internal}}+k_{-{AMP}^{'}}k_{-AMP} \right)}$$

$$=\frac{1}{2}\left( \frac{k_{+internal}\frac{k_{+T}\left[ T \right]}{K_{T,c}+\left[ T \right]}}{\frac{k_{+T}\left[ T \right]}{K_{T,c}+\left[ T \right]}+k_{-T}+k_{+internal}}+k_{+{AMP}^{'}}+k_{-A{MP}^{'}}+k_{-AMP} \right.$$

$$\pm\sqrt{\left( \frac{k_{+internal}\frac{k_{+T}\left[ T \right]}{K_{T,c}+\left[ T \right]}}{\frac{k_{+T}\left[ T \right]}{K_{T,c}+\left[ T \right]}+k_{-T}+k_{+internal}}-k_{+A{MP}^{'}}-k_{-{AMP}^{'}}+k_{-AMP} \right)^{2}+4\left( k_{+A{MP}^{'}}k_{-AMP} \right)}$$

$$\begin{aligned} \#S81 \end{aligned}$$

The observed rate constant for the fast phase can be approximated as

$$\lambda_{+}=\frac{1}{2}\left( \frac{k_{+internal}\frac{k_{+T}\left[ T \right]}{K_{T,c}+\left[ T \right]}}{\frac{k_{+T}\left[ T \right]}{K_{T,c}+\left[ T \right]}+k_{-T}+k_{+internal}}+k_{+{AMP}^{'}}+k_{-{AMP}^{'}}+k_{-AMP} \right.$$

$$\pm\sqrt{\left( \frac{k_{+internal}\frac{k_{+T}\left[ T \right]}{K_{T,c}+\left[ T \right]}}{\frac{k_{+T}\left[ T \right]}{K_{T,c}+\left[ T \right]}+k_{-T}+k_{+internal}}-k_{+A{MP}^{'}}-k_{-A{MP}^{'}}+k_{-AMP} \right)^{2}+4\left( k_{+{AMP}^{'}}k_{-AMP} \right)}$$

$$\gtrsim\frac{1}{2}\left( \frac{k_{+internal}\frac{k_{+T}\left[ T \right]}{K_{T,c}+\left[ T \right]}}{\frac{k_{+T}\left[ T \right]}{K_{T,c}+\left[ T \right]}+k_{-T}+k_{+internal}}+k_{+{AMP}^{'}}+k_{-{AMP}^{'}}+k_{-AMP} \right.$$

$$+\sqrt{\left( \frac{k_{+internal}\frac{k_{+T}\left[ T \right]}{K_{T,c}+\left[ T \right]}}{\frac{k_{+T}\left[ T \right]}{K_{T,c}+\left[ T \right]}+k_{-T}+k_{+internal}}-k_{+A{MP}^{'}}-k_{-A{MP}^{'}}+k_{-AMP} \right)^{2}}$$

$$=\frac{k_{+internal}\frac{k_{+T}\left[ T \right]}{K_{T,c}+\left[ T \right]}}{\frac{k_{+T}\left[ T \right]}{K_{T,c}+\left[ T \right]}+k_{-T}+k_{+internal}}+k_{-AMP}=\frac{k_{+internal}}{1+\frac{k_{-T}+k_{+internal}}{\frac{k_{+T}\left[ T \right]}{K_{T,c}+\left[ T \right]}}}+k_{-AMP}$$

$$\begin{aligned} \sim k_{+internal}+k_{-AMP}\#S82 \end{aligned}$$

According to Eq. S81, if we plug in this value for *λ*_obs,fast_, *λ*_obs,slow_ is:

$$\begin{aligned} \lambda_{-}=\frac{\lambda_{-}\lambda_{+}}{\lambda_{+}}\sim\frac{\frac{\left( k_{+{AMP}^{'}}+k_{-{AMP}^{'}} \right)k_{+internal}\frac{k_{+T}\left[ T \right]}{K_{T,c}+\left[ T \right]}}{\frac{k_{+T}\left[ T \right]}{K_{T,c}+\left[ T \right]}+k_{-T}+k_{+internal}}+k_{-{AMP}^{'}}k_{-AMP}}{\frac{k_{+internal}\frac{k_{+T}\left[ T \right]}{K_{T,c}+\left[ T \right]}}{\frac{k_{+T}\left[ T \right]}{K_{T,c}+\left[ T \right]}+k_{-T}+k_{+internal}}+k_{-AMP}} \end{aligned}$$

$$=\frac{\frac{\left( k_{+{AMP}^{'}}+k_{-{AMP}^{'}} \right)k_{+internal}}{1+\frac{k_{-T}+k_{+internal}}{\frac{k_{+T}\left[ T \right]}{K_{T,c}+\left[ T \right]}}}+k_{-{AMP}^{'}}k_{-AMP}}{\frac{k_{+internal}}{1+\frac{k_{-T}+k_{+internal}}{\frac{k_{+T}\left[ T \right]}{K_{T,c}+\left[ T \right]}}}+k_{-AMP}}\sim\frac{\left( k_{+A{MP}^{'}}+k_{-{AMP}^{'}} \right)k_{+internal}+k_{-A{MP}^{'}}k_{-AMP}}{k_{+internal}+k_{-AMP}}$$

$$\begin{aligned} \sim k_{+A{MP}^{'}}+k_{-A{MP}^{'}}\#S83 \end{aligned}$$

With the approximated fast and slow observed rate constants in Eq. S82 and S83, the general solution for the system of differential equations comprised of Eq. S79 and S3 is

$$\begin{aligned} \left( \begin{matrix} \left[ E\cdot M \right] \\ \left[ E\cdot M^{'} \right] \end{matrix} \right)=C_{+}\left( \begin{matrix} 1 \\ \frac{k_{+{AMP}^{'}}}{-\lambda_{+}+k_{-{AMP}^{'}}} \end{matrix} \right)e^{-\lambda_{+}t}+C_{-}\left( \begin{matrix} 1 \\ \frac{k_{+{AMP}^{'}}}{-\lambda_{-}+k_{-A{MP}^{'}}} \end{matrix} \right)e^{-\lambda_{-}t}+\left( \begin{matrix} X_{1} \\ X_{2} \end{matrix} \right)\#S84 \end{aligned}$$

The coefficients *C*_+_, *C*_−_, *X*_1_, and *X*_2_ are constants and *X*_1_, and *X*_2_ must satisfy

$$\left( \begin{matrix} \frac{k_{+internal}\frac{k_{+T}\left[ T \right]}{K_{T,c}+\left[ T \right]}}{\frac{k_{+T}\left[ T \right]}{K_{T,c}+\left[ T \right]}+k_{-T}+k_{+internal}}+k_{+A{MP}^{'}}+k_{-AMP} & \frac{k_{+internal}\frac{k_{+T}\left[ T \right]}{K_{T,c}+\left[ T \right]}}{\frac{k_{+T}\left[ T \right]}{K_{T,c}+\left[ T \right]}+k_{-T}+k_{+internal}}-k_{-A{MP}^{'}} \\ -k_{+A{MP}^{'}} & k_{-{AMP}^{'}} \end{matrix} \right)\left( \begin{matrix} X_{1} \\ X_{2} \end{matrix} \right)$$

$$\begin{aligned} =\left( \begin{matrix} \frac{k_{+internal}\frac{k_{+T}\left[ T \right]E_{0}}{K_{T,c}+\left[ T \right]}}{\frac{k_{+T}\left[ T \right]}{K_{T,c}+\left[ T \right]}+k_{-T}+k_{+internal}} \\ 0 \end{matrix} \right)\#S85 \end{aligned}$$

Solving Eq. S85 for *X*_1_, and *X*_2_, we obtain

$$X_{1}=\frac{\left| \begin{matrix} \frac{k_{+internal}\frac{k_{+T}\left[ T \right]E_{0}}{K_{T,c}+\left[ T \right]}}{\frac{k_{+T}\left[ T \right]}{K_{T,c}+\left[ T \right]}+k_{-T}+k_{+internal}} & \frac{k_{+internal}\frac{k_{+T}\left[ T \right]}{K_{T,c}+\left[ T \right]}}{\frac{k_{+T}\left[ T \right]}{K_{T,c}+\left[ T \right]}+k_{-T}+k_{+internal}}-k_{-{AMP}^{'}} \\ 0 & k_{-{AMP}^{'}} \end{matrix} \right|}{\left| \begin{matrix} \frac{k_{+internal}\frac{k_{+T}\left[ T \right]}{K_{T,c}+\left[ T \right]}}{\frac{k_{+T}\left[ T \right]}{K_{T,c}+\left[ T \right]}+k_{-T}+k_{+internal}}+k_{+A{MP}^{'}}+k_{-AMP} & \frac{k_{+internal}\frac{k_{+T}\left[ T \right]}{K_{T,c}+\left[ T \right]}}{\frac{k_{+T}\left[ T \right]}{K_{T,c}+\left[ T \right]}+k_{-T}+k_{+internal}}-k_{-{AMP}^{'}} \\ -k_{+A{MP}^{'}} & k_{-{AMP}^{'}} \end{matrix} \right|}$$

$$=\frac{\frac{k_{-{AMP}^{'}}k_{+internal}\frac{k_{+T}\left[ T \right]E_{0}}{K_{T,c}+\left[ T \right]}}{\frac{k_{+T}\left[ T \right]}{K_{T,c}+\left[ T \right]}+k_{-T}+k_{+internal}}}{k_{-M^{'}}\left( \frac{k_{+internal}\frac{k_{+T}\left[ T \right]}{K_{T,c}+\left[ T \right]}}{\frac{k_{+T}\left[ T \right]}{K_{T,c}+\left[ T \right]}+k_{-T}+k_{+internal}}+k_{+A{MP}^{'}}+k_{-AMP} \right)+k_{+{AMP}^{'}}\left( \frac{k_{+internal}\frac{k_{+T}\left[ T \right]}{K_{T,c}+\left[ T \right]}}{\frac{k_{+T}\left[ T \right]}{K_{T,c}+\left[ T \right]}+k_{-T}+k_{+internal}}-k_{-{AMP}^{'}} \right)}$$

$$=\frac{\frac{k_{-{AMP}^{'}}k_{+internal}\frac{k_{+T}\left[ T \right]E_{0}}{K_{T,c}+\left[ T \right]}}{\frac{k_{+T}\left[ T \right]}{K_{T,c}+\left[ T \right]}+k_{-T}+k_{+internal}}}{\frac{{\left( k_{+A{MP}^{'}}+k_{-{AMP}^{'}} \right)k}_{+internal}\frac{k_{+T}\left[ T \right]}{K_{T,c}+\left[ T \right]}}{\frac{k_{+T}\left[ T \right]}{K_{T,c}+\left[ T \right]}+k_{-T}+k_{+internal}}+k_{-A{MP}^{'}}k_{-AMP}}\sim\frac{k_{-A{MP}^{'}}k_{+internal}E_{0}}{{\left( k_{+{AMP}^{'}}+k_{-{AMP}^{'}} \right)k}_{+internal}+k_{-{AMP}^{'}}k_{-AMP}}$$

$$\begin{aligned} \sim\frac{k_{-{AMP}^{'}}E_{0}}{k_{+A{MP}^{'}}+k_{-A{MP}^{'}}}\#S86 \end{aligned}$$

$$X_{2}=\frac{\left| \begin{matrix} \frac{k_{+internal}\frac{k_{+T}\left[ T \right]}{K_{T,c}+\left[ T \right]}}{\frac{k_{+T}\left[ T \right]}{K_{T,c}+\left[ T \right]}+k_{-T}+k_{+internal}}+k_{+A{MP}^{'}}+k_{-AMP} & \frac{k_{+internal}\frac{k_{+T}\left[ T \right]E_{0}}{K_{T,c}+\left[ T \right]}}{\frac{k_{+T}\left[ T \right]}{K_{T,c}+\left[ T \right]}+k_{-T}+k_{+internal}}k_{-A{MP}^{'}} \\ -k_{+{AM}^{'}} & 0 \end{matrix} \right|}{\left| \begin{matrix} \frac{k_{+internal}\frac{k_{+T}\left[ T \right]}{K_{T,c}+\left[ T \right]}}{\frac{k_{+T}\left[ T \right]}{K_{T,c}+\left[ T \right]}+k_{-T}+k_{+internal}}+k_{+A{MP}^{'}}+k_{-AMP} & \frac{k_{+internal}\frac{k_{+T}\left[ T \right]}{K_{T,c}+\left[ T \right]}}{\frac{k_{+T}\left[ T \right]}{K_{T,c}+\left[ T \right]}+k_{-T}+k_{+internal}}-k_{-{AMP}^{'}} \\ -k_{+{AMP}^{'}} & k_{-{AMP}^{'}} \end{matrix} \right|}$$

$$\begin{aligned} =\frac{\frac{k_{+A{MP}^{'}}k_{+internal}\frac{k_{+T}\left[ T \right]E_{0}}{K_{T,c}+\left[ T \right]}}{\frac{k_{+T}\left[ T \right]}{K_{T,c}+\left[ T \right]}+k_{-T}+k_{+internal}}}{\frac{{\left( k_{+{AMP}^{'}}+k_{-{AMP}^{'}} \right)k}_{+internal}\frac{k_{+T}\left[ T \right]}{K_{T,c}+\left[ T \right]}}{\frac{k_{+T}\left[ T \right]}{K_{T,c}+\left[ T \right]}+k_{-T}+k_{+internal}}+k_{-{AMP}^{'}}k_{-AMP}}\sim\frac{k_{+{AMP}^{'}}E_{0}}{k_{+A{MP}^{'}}+k_{-A{MP}^{'}}}\#S87 \end{aligned}$$

The constants *C*_+_ and *C*_−_ are determined from the initial values of the two states and they should both be 0 at *t* = 0. Using the general expression of the solution (Eq. S84):

$$\left. \left( \begin{matrix} \left[ E\cdot M \right] \\ \left[ E{\cdot M}^{'} \right] \end{matrix} \right) \right|_{t=0} =\left( \begin{matrix} 0 \\ 0 \end{matrix} \right)=C_{+}\left( \begin{matrix} 1 \\ \frac{k_{+{AMP}^{'}}}{-\lambda_{+}+k_{-{AMP}^{'}}} \end{matrix} \right)e^{-\lambda_{+}t}+C_{-}\left( \begin{matrix} 1 \\ \frac{k_{+A{MP}^{'}}}{-\lambda_{-}+k_{-A{MP}^{'}}} \end{matrix} \right)e^{-\lambda_{-}t}+\left( \begin{matrix} X_{1} \\ X_{2} \end{matrix} \right)$$

i.e.,

$$\begin{aligned} C_{+}+C_{-}=-X_{1}\#S88 \end{aligned}$$

$$\begin{aligned} C_{+}\frac{k_{+{AMP}^{'}}}{-\lambda_{+}+k_{-{AMP}^{'}}}+C_{-}\frac{k_{+{AMP}^{'}}}{-\lambda_{-}+k_{-A{MP}^{'}}}=-X_{2}\#S89 \end{aligned}$$

$$C_{+}=\frac{\left| \begin{matrix} -X_{1} & 1 \\ -X_{2} & \frac{k_{+{AMP}^{'}}}{-\lambda_{-}+k_{-A{MP}^{'}}} \end{matrix} \right|}{\left| \begin{matrix} 1 & 1 \\ \frac{k_{+{AMP}^{'}}}{-\lambda_{+}+k_{-A{MP}^{'}}} & \frac{k_{+A{MP}^{'}}}{-\lambda_{-}+k_{-{AMP}^{'}}} \end{matrix} \right|}=\frac{-X_{1}\frac{k_{+A{MP}^{'}}}{-\lambda_{-}+k_{-{AMP}^{'}}}+X_{2}}{\frac{k_{+{AMP}^{'}}}{-\lambda_{-}+k_{-A{MP}^{'}}}-\frac{k_{+{AMP}^{'}}}{-\lambda_{+}+k_{-A{MP}^{'}}}}$$

$$=\frac{1}{k_{+{AMP}^{'}}}\frac{-k_{-{AMP}^{'}}\left( -\lambda_{+}+k_{-A{MP}^{'}} \right)+\left( -\lambda_{+}+k_{-A{MP}^{'}} \right)\left( -\lambda_{-}+k_{-A{MP}^{'}} \right)}{-\lambda_{+}+k_{-A{MP}^{'}}+\lambda_{-}-k_{-A{MP}^{'}}}X_{2}$$

$$\begin{aligned} =\frac{X_{2}}{k_{+{AMP}^{'}}}\frac{\left( \lambda_{+}-k_{-A{MP}^{'}} \right)\lambda_{-}}{-\lambda_{+}+\lambda_{-}}\#S90 \end{aligned}$$

$$C_{-}=\frac{\left| \begin{matrix} 1 & -X_{1} \\ \frac{k_{+A{MP}^{'}}}{-\lambda_{+}+k_{-A{MP}^{'}}} & -X_{2} \end{matrix} \right|}{\left| \begin{matrix} 1 & 1 \\ \frac{k_{+{AMP}^{'}}}{-\lambda_{+}+k_{-{AMP}^{'}}} & \frac{k_{+A{MP}^{'}}}{-\lambda_{-}+k_{-{AMP}^{'}}} \end{matrix} \right|}=\frac{-X_{2}+X_{1}\frac{k_{+{AMP}^{'}}}{-\lambda_{+}+k_{-{AMP}^{'}}}}{\frac{k_{+A{MP}^{'}}}{-\lambda_{-}+k_{-A{MP}^{'}}}-\frac{k_{+A{MP}^{'}}}{-\lambda_{+}+k_{-A{MP}^{'}}}}$$

$$=\frac{-1+\frac{k_{-{AMP}^{'}}}{-\lambda_{+}+k_{-A{MP}^{'}}}}{\frac{k_{+A{MP}^{'}}}{-\lambda_{-}+k_{-{AMP}^{'}}}-\frac{k_{+A{MP}^{'}}}{-\lambda_{+}+k_{-{AMP}^{'}}}}X_{2}$$

$$=\frac{1}{k_{+A{MP}^{'}}}\frac{-\left( -\lambda_{+}+k_{-A{MP}^{'}} \right)\left( -\lambda_{-}+k_{-{AMP}^{'}} \right)+k_{-{AMP}^{'}}\left( -\lambda_{-}+k_{-A{MP}^{'}} \right)}{-\lambda_{+}+\lambda_{-}}X_{2}$$

$$\begin{aligned} =-\frac{X_{2}}{k_{+{AMP}^{'}}}\frac{\lambda_{+}\left( \lambda_{-}-k_{-{AMP}^{'}} \right)}{-\lambda_{+}+\lambda_{-}}\#S91 \end{aligned}$$

Finally, according to Eq. S84, S90 and S91 the solution is

$$\left( \begin{matrix} \left[ E\cdot M \right] \\ \left[ E\cdot M^{'} \right] \end{matrix} \right)=\frac{X_{2}}{k_{+{AMP}^{'}}}\frac{\left( \lambda_{+}-k_{-A{MP}^{'}} \right)\lambda_{-}}{-\lambda_{+}+\lambda_{-}}\left( \begin{matrix} 1 \\ \frac{k_{+A{MP}^{'}}}{-\lambda_{+}+k_{-{AMP}^{'}}} \end{matrix} \right)e^{-\lambda_{+}t}$$

$$-\frac{X_{2}}{k_{+{AMP}^{'}}}\frac{\lambda_{+}\left( \lambda_{-}-k_{-A{MP}^{'}} \right)}{-\lambda_{+}+\lambda_{-}}\left( \begin{matrix} 1 \\ \frac{k_{+A{MP}^{'}}}{-\lambda_{-}+k_{-A{MP}^{'}}} \end{matrix} \right)e^{-\lambda_{-}t}+\left( \begin{matrix} k_{-A{MP}^{'}} \\ k_{+{AMP}^{'}} \end{matrix} \right)\frac{X_{2}}{k_{+A{MP}^{'}}}$$

$$=\frac{X_{2}}{k_{+{AMP}^{'}}}\left( \frac{\left( \lambda_{+}-k_{-A{MP}^{'}} \right)\lambda_{-}}{-\lambda_{+}+\lambda_{-}}\left( \begin{matrix} 1 \\ \frac{k_{+{AMP}^{'}}}{-\lambda_{+}+k_{-{AMP}^{'}}} \end{matrix} \right)e^{-\lambda_{+}t} \right.-\frac{\lambda_{+}\left( \lambda_{-}-k_{-{AMP}^{'}} \right)}{-\lambda_{+}+\lambda_{-}}\left( \begin{matrix} 1 \\ \frac{k_{+A{MP}^{'}}}{-\lambda_{-}+k_{-{AMP}^{'}}} \end{matrix} \right)e^{-\lambda_{-}t}$$

$$\begin{aligned} \left. +\left( \begin{matrix} k_{-{AMP}^{'}} \\ k_{+A{MP}^{'}} \end{matrix} \right) \right)\#S92 \end{aligned}$$

According to the Scheme 1, the initial AMP release rate (not considering product inhibition) is

$$\frac{d\left[ M \right]}{dt}=k_{-AMP}\left[ E\cdot M \right]$$

$$\begin{aligned} =\frac{{k_{-AMP}X}_{2}}{k_{+{AMP}^{'}}}\left( \frac{\left( \lambda_{+}-k_{-{AMP}^{'}} \right)\lambda_{-}}{-\lambda_{+}+\lambda_{-}}e^{-\lambda_{+}t}-\frac{\lambda_{+}\left( \lambda_{-}-k_{-{AMP}^{'}} \right)}{-\lambda_{+}+\lambda_{-}}e^{-\lambda_{-}t}+k_{-A{MP}^{'}} \right)\#S93 \end{aligned}$$

Integrating Eq. S93 yields

$$\begin{aligned} \left[ M \right]=\frac{{k_{-AMP}X}_{2}}{k_{+{AMP}^{'}}}\left( -\frac{\left( \lambda_{+}-k_{-A{MP}^{'}} \right)\lambda_{-}}{\lambda_{+}\left( \lambda_{+}-\lambda_{-} \right)}\left( 1-e^{-\lambda_{+}t} \right)+\frac{\lambda_{+}\left( \lambda_{-}-k_{-A{MP}^{'}} \right)}{\lambda_{-}\left( \lambda_{+}-\lambda_{-} \right)}\left( {1-e}^{-\lambda_{-}t} \right)+k_{-A{MP}^{'}}t \right)\#S94 \end{aligned}$$

The initial condition of [*M*] = 0 has been incorporated into Eq. S94. The measured [AMP] at any given time point in multi-turnover quenched-flow experiments is the sum of both the free and enzyme-bound AMP states (M, E∙M, and E∙M′). This sum can be expressed (according to Eq. S92 and S94) as

$$\left[ E\cdot M \right]+\left[ E\cdot M^{'} \right]+\left[ M \right]=\frac{X_{2}}{k_{+{AMP}^{'}}}\left( -\frac{\left( \lambda_{+}-k_{-A{MP}^{'}} \right)\lambda_{-}}{\lambda_{+}-\lambda_{-}}\left( 1-\frac{k_{+{AMP}^{'}}}{\lambda_{+}-k_{-{AMP}^{'}}} \right)e^{-\lambda_{+}t} \right.$$

$$+k_{-AMP}\frac{\left( \lambda_{+}-k_{-A{MP}^{'}} \right)\lambda_{-}}{\lambda_{+}\left( \lambda_{+}-\lambda_{-} \right)}e^{-\lambda_{+}t}-k_{-AMP}\frac{\left( \lambda_{+}-k_{-A{MP}^{'}} \right)\lambda_{-}}{\lambda_{+}\left( \lambda_{+}-\lambda_{-} \right)}$$

$$+\frac{\lambda_{+}\left( \lambda_{-}-k_{-A{MP}^{'}} \right)}{\lambda_{+}-\lambda_{-}}\left( 1-\frac{k_{+A{MP}^{'}}}{\lambda_{-}-k_{-A{MP}^{'}}} \right)e^{-\lambda_{-}t}-k_{-AMP}\frac{\lambda_{+}\left( \lambda_{-}-k_{-{AMP}^{'}} \right)}{\lambda_{-}\left( \lambda_{+}-\lambda_{-} \right)}e^{-\lambda_{-}t}$$

$$\left. +k_{-AMP}\frac{\lambda_{+}\left( \lambda_{-}-k_{-{AMP}^{'}} \right)}{\lambda_{-}\left( \lambda_{+}-\lambda_{-} \right)}+k_{-{AMP}^{'}}+k_{+{AMP}^{'}}+k_{-A{MP}^{'}}k_{-AMP}t \right)$$

$$=\frac{X_{2}}{k_{+{AMP}^{'}}}\left( -\frac{\lambda_{-}}{\lambda_{+}-\lambda_{-}}\left( \lambda_{+}-k_{+A{MP}^{'}}-k_{-A{MP}^{'}}-k_{-AMP}\frac{\left( \lambda_{+}-k_{-{AMP}^{'}} \right)}{\lambda_{+}} \right)e^{-\lambda_{+}t} \right.$$

$$+\frac{\lambda_{+}}{\lambda_{+}-\lambda_{-}}\left( \lambda_{-}-k_{+A{MP}^{'}}-k_{-A{MP}^{'}}-k_{-AMP}\frac{\left( \lambda_{-}-k_{-{AMP}^{'}} \right)}{\lambda_{-}} \right)e^{-\lambda_{-}t}$$

$$+k_{-A{MP}^{'}}k_{-AMP}t-k_{-AMP}\frac{\left( \lambda_{+}-k_{-A{MP}^{'}} \right)\lambda_{-}}{\lambda_{+}\left( \lambda_{+}-\lambda_{-} \right)}\left. +k_{-AMP}\frac{\lambda_{+}\left( \lambda_{-}-k_{-{AMP}^{'}} \right)}{\lambda_{-}\left( \lambda_{+}-\lambda_{-} \right)}+k_{-A{MP}^{'}}+k_{+A{MP}^{'}} \right)$$

$$\sim\frac{E_{0}}{k_{+{AMP}^{'}}+k_{-A{MP}^{'}}}\left( -\frac{\lambda_{-}}{\lambda_{+}-\lambda_{-}}\left( \lambda_{+}-k_{+{AMP}^{'}}-k_{-A{MP}^{'}}-k_{-AMP}\frac{\left( \lambda_{+}-k_{-{AMP}^{'}} \right)}{\lambda_{+}} \right)e^{-\lambda_{+}t} \right.$$

$$+\frac{\lambda_{+}}{\lambda_{+}-\lambda_{-}}\left( \lambda_{-}-k_{+A{MP}^{'}}-k_{-{AMP}^{'}}-k_{-AMP}\frac{\left( \lambda_{-}-k_{-A{MP}^{'}} \right)}{\lambda_{-}} \right)e^{-\lambda_{-}t}$$

$$\begin{aligned} +k_{-A{MP}^{'}}k_{-AMP}t-k_{-AMP}\frac{\left( \lambda_{+}-k_{-{AMP}^{'}} \right)\lambda_{-}}{\lambda_{+}\left( \lambda_{+}-\lambda_{-} \right)}\left. +k_{-AMP}\frac{\lambda_{+}\left( \lambda_{-}-k_{-{AMP}^{'}} \right)}{\lambda_{-}\left( \lambda_{+}-\lambda_{-} \right)}+k_{-{AMP}^{'}}+k_{+A{MP}^{'}} \right)\#S95 \end{aligned}$$

*X*_2_ in Eq. S87 is used in the above derivation. The coefficient $-\frac{\lambda_{-}}{\lambda_{+}-\lambda_{-}}\left( \lambda_{+}-k_{+A{MP}^{'}}-k_{-{AMP}^{'}}-k_{-AMP}\frac{\left( \lambda_{+}-k_{-{AMP}^{'}} \right)}{\lambda_{+}} \right)$ associated with the fast phase exponential *λ*_+_ is negative, indicating the exponential phase increases with time. According to Eq. S82, $\lambda_{+}\sim k_{+internal}+k_{-AMP}\gg k_{+{AMP}^{'}}+k_{-{AMP}^{'}}+k_{-AMP}\frac{\left( \lambda_{+}-k_{-{AMP}^{'}} \right)}{\lambda_{+}}$, such that $\frac{\lambda_{-}}{\lambda_{+}-\lambda_{-}}\left( \lambda_{+}-k_{+A{MP}^{'}}-k_{-A{MP}^{'}}-k_{-AMP}\frac{\left( \lambda_{+}-k_{-{AMP}^{'}} \right)}{\lambda_{+}} \right)$ is not trivial in comparison. The coefficient $\frac{\lambda_{+}}{\lambda_{+}-\lambda_{-}}\left( \lambda_{-}-k_{+A{MP}^{'}}-k_{-{AMP}^{'}}-k_{-AMP}\frac{\left( \lambda_{-}-k_{-A{MP}^{'}} \right)}{\lambda_{-}} \right)$ associated with the slow phase exponential *λ*_−_ is positive, indicating the exponential phase is a decay. According to Eq. S83, $\lambda_{-}\sim k_{+{AMP}^{'}}+k_{-A{MP}^{'}}$, such that $\frac{\lambda_{+}}{\lambda_{+}-\lambda_{-}}\left( \lambda_{-}-k_{+A{MP}^{'}}-k_{-{AMP}^{'}}-k_{-AMP}\frac{\left( \lambda_{-}-k_{-{AMP}^{'}} \right)}{\lambda_{-}} \right)\sim0$ is a trivial value. In short, the slow phase associated exponential decay has a very small amplitude and may not observable under experimental conditions. The increasing fast phase exponential dominates during the burst phase before the system reaches steady state. Steady state AMP release is associated with the term linearly increasing with time. According to Eq. S95, the steady state AMP production rate is $\frac{k_{-{AMP}^{'}}k_{-AMP}E_{0}}{k_{+{AMP}^{'}}+k_{-A{MP}^{'}}}=\frac{k_{-AMP}E_{0}}{1+\frac{k_{+{AMP}^{'}}}{k_{-{AMP}^{'}}}}<k_{-AMP}E_{0}$, which, under our specific conditions, is identical to *k*_cat,ATP_ (Eq. S43; $k_{cat}=\frac{k_{-AMP}}{1+\frac{k_{+{AMP}^{'}}}{k_{-{AMP}^{'}}}+\frac{k_{-AMP}}{k_{+T}}}\approx\frac{k_{-AMP}}{1+\frac{k_{+{AMP}^{'}}}{k_{-{AMP}^{'}}}}$) multiplied by the initial [ENPP1].

1. ***Overall AMP binding affinit******y***

The overall (non-covalent) AMP binding affinity reflects the sum of both AMP-bound ENPP1 states (E∙M + E∙M′).

$$\begin{aligned} E+M\underset{\rightleftharpoons}{K_{AMP,c}}\left( E\cdot M \right)\underset{k_{-AMP}}{\underset{\rightleftharpoons}{k_{+AMP}}}E\cdot M\underset{k_{-AMP}'}{\underset{\rightleftharpoons}{k_{+AMP^{'}}}}E\cdot M^{'}\# Scheme 3 \\ \end{aligned}$$

The collision complex ((E∙M)) has an AMP affinity of 3 µM (Fig. 4, Table 1), and is given by:

$$\begin{aligned} K_{AMP,c}=\frac{\left[ E \right]\left[ M \right]}{\left[ \left( E\cdot M \right) \right]}\sim3 \mu M\#S96 \end{aligned}$$

The two non-covalent AMP-bound state (E∙M and E∙M) equilibrium constants are related to the microscopic rate constants (Table 1) as follows:

$$\begin{aligned} K_{AMP}=\frac{\left[ \left( E\cdot M \right) \right]}{\left[ E\cdot M \right]}=\frac{k_{-AMP}}{k_{+AMP}}=\frac{8 {sec}^{-1}}{565 {sec}^{-1}}=0.014\#S97 \end{aligned}$$

$$\begin{aligned} K_{AMP^{'}}=\frac{\left[ E\cdot M \right]}{\left[ E\cdot M^{'} \right]}=\frac{k_{-AMP^{'}}}{k_{+AMP^{'}}} \sim1\#S98 \end{aligned}$$

Combining, rearranging and summing these relationships yields an overall AMP binding affinity of ~21 nM as follows:

$$K_{AMP, overall}=\frac{\left[ E \right]\left[ M \right]}{\left[ EM \right]+\left[ EM^{'} \right]}=\frac{\left[ E \right]\left[ M \right]}{\frac{\left[ \left( E\cdot M \right) \right]}{K_{AMP}}+\frac{\left[ EM \right]}{K_{AMP^{'}}}}$$

$$\begin{aligned} =\frac{\left[ E \right]\left[ M \right]}{\frac{\left[ \left( E\cdot M \right) \right]}{K_{AMP}}+\frac{1}{K_{AMP^{'}}}\frac{\left[ \left( E\cdot M \right) \right]}{K_{AMP}}}=\frac{K_{AMP}K_{AMP^{'}}}{K_{AMP^{'}}+1}\frac{\left[ E \right]\left[ M \right]}{\left[ \left( E\cdot M \right) \right]}=K_{M,c}\frac{K_{AMP}K_{AMP^{'}}}{K_{AMP^{'}}+1}\#S99 \end{aligned}$$

$$=\frac{3 \mu M\times1\times0.014}{1+1}=0.021 \mu M$$
